# Asymmetric Primaquine and Halogenaniline Fumardiamides as Novel Biologically Active Michael Acceptors

**DOI:** 10.3390/molecules23071724

**Published:** 2018-07-14

**Authors:** Zrinka Rajić, Maja Beus, Hana Michnová, Josipa Vlainić, Leentje Persoons, Ivan Kosalec, Josef Jampílek, Dominique Schols, Toma Keser, Branka Zorc

**Affiliations:** 1 Faculty of Pharmacy and Biochemistry, University of Zagreb, A. Kovačića 1, 10000 Zagreb, Croatia; mbeus@pharma.hr (M.B.); ikosalec@pharma.hr (I.K.); tkeser@pharma.hr (T.K.); 2 Department of Pharmaceutical Chemistry, Faculty of Pharmacy, Comenius University, Odbojárov 10, 83232 Bratislava, Slovakia; michnova.hana@gmail.com (H.M.), josef.jampilek@gmail.com (J.J.); 3 Laboratory for Advanced Genomics, Division of Molecular Medicine, Rudjer Bošković Institute, Bijenička cesta 54, 10000 Zagreb, Croatia; josipa.vlainic@irb.hr; 4 Laboratory of Virology and Chemotherapy, Rega Institute for Medical Research, KU Leuven, Herestraat 49, 3000 Leuven, Belgium; leentje.persoons@rega.kuleuven.be (L.P.); dominique.schols@rega.kuleuven.be (D.S.)

**Keywords:** fumardiamide, primaquine, succindiamide, Michael acceptor, biofilm eradication, antibacterial screening, antiviral activity, cytostatic activity

## Abstract

Novel primaquine (PQ) and halogenaniline asymmetric fumardiamides **4a**–**f**, potential Michael acceptors, and their reduced analogues succindiamides **5a**–**f** were prepared by simple three-step reactions: coupling reaction between PQ and mono-ethyl fumarate (**1a**) or mono-methyl succinate (**1b**), hydrolysis of PQ-dicarboxylic acid mono-ester conjugates **2a**,**b** to corresponding acids **3a**,**b**, and a coupling reaction with halogenanilines. 1-[bis(Dimethylamino)methylene]-1*H*-1,2,3-triazolo[4,5-*b*]pyridinium 3-oxide hexafluorophosphate (HATU) was used as a coupling reagent along with Hünig′s base. Compounds **4** and **5** were evaluated against a panel of bacteria, several *Mycobacterium* strains, fungi, a set of viruses, and nine different human tumor cell lines. *p*-Chlorofumardiamide **4d** showed significant activity against *Staphylococcus aureus,* *Streptococcus pneumoniae* and *Acinetobacter baumannii*, but also against *Candida albicans* (minimum inhibitory concentration (MIC) 6.1–12.5 µg/mL). Together with *p*-fluoro and *p*-CF_3_ fumardiamides **4b**,**f**, compound **4d** showed activity against *Mycobacterium marinum* and **4b**,**f** against *M. tuberculosis*. In biofilm eradication assay, most of the bacteria, particularly *S. aureus*, showed susceptibility to fumardiamides. *m*-CF_3_ and *m*-chloroaniline fumardiamides **4e** and **4c** showed significant antiviral activity against reovirus-1, sindbis virus and Punta Toro virus (EC_50_ = 3.1–5.5 µM), while **4e** was active against coxsackie virus B4 (EC_50_ = 3.1 µM). *m*-Fluoro derivative **4a** exerted significant cytostatic activity (IC_50_ = 5.7–31.2 μM). Acute lymphoblastic leukemia cells were highly susceptible towards *m*-substituted derivatives **4a**,**c**,**e** (IC_50_ = 6.7–8.9 μM). Biological evaluations revealed that fumardiamides **4** were more active than succindiamides **5** indicating importance of Michael conjugated system.

## 1. Introduction

**Figure 1:**
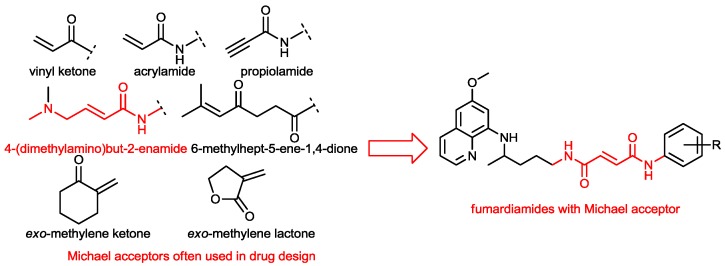
Design of novel fumardiamides.

Compounds bearing α,β-unsaturated carbonyl groups are Michael acceptors capable of conjugate addition, also known as Michael addition. The simplest and the best Michael acceptors are α,β-unsaturated carbonyl compounds with exposed unsaturated β-carbon atoms, such as exomethylene ketones and lactones or vinyl ketones [1]. These fragments are often used in the design of new anticancer drugs, together with others (6-methylhept-5-ene-1,4-dione, propiolamide, 4-(dimethylamino)but-2-enamide) (Figure 1). They assure the irreversible covalent binding to a cysteine residue of a specific protein and may modulate selectivity and potency of the drug candidate. The targeted covalent modification has emerged as a validated approach to drug discovery with the drug candidate canertinib [2], approved drugs afatinib, neratinib and osimertinib (inhibitors of human epidermal growth factor receptors) and ibrutinib (Bruton′s tyrosine kinase inhibitor) [3,4]. A comprehensive review published by Jackson et al. gives an overview of biological activity and applicability of various Michael acceptors [5].

Michael acceptors have been explored in a prodrug strategy for cancer cell-specific targeting. In the review published by Zhang et al., two doxorubicin prodrugs with maleimide moieties have been described [6]. The maleimide component is responsible for the binding to human serum albumin. Once the drug carrier arrives at the targeted cancer tissue, doxorubicin is released from the carrier by the cleavage of hydrazone or glycosidic bond in the acidic environment of cancer cells. The prodrugs demonstrate superior anticancer efficacy than the parent drug.

Michael acceptors are present in other classes of drugs. Examples of such drugs are entacapone (antiparkinsonic) [7], dimethyl fumarate (antipsoriatic; since 2013 used in treatment of multiple sclerosis) [5], rupintivir (experimental antiviral drug against human rhinoviruses) [8], exemestane (cytostatic) [9], and ethacrynic acid (diuretic) [10].

α,β-Unsaturated carbonyl group is also a motif found in plant and microbial metabolites and their semisynthetic/synthetic derivatives. Many of them are used in clinical practice or are still under the evaluation in clinical trials (vernolepin, helenalin, curcumin, pyrrocidine, fumaric and angelic acid derivatives), but some are classified as toxins [11]. Vernolepin and helenalin are sesquiterpenes with exomethylene lactones responsible for irreversible DNA polymerase inhibition. Pyrrocidine A is a 13-membered macrocyclic alkaloid produced by endophytic fungi, which directly binds to *N*-acetyl-l-cysteine methyl ester through the Michael-type addition and exerts both antimicrobial and cytostatic effect on leukemia HL60 cells [12]. Angelic acid ester ingenol mebutate has been identified as the most active component of *Euphorbia peplus* L. latex sap, effective against human nonmelanoma skin cancer [13] and actinic keratosis [14]. A gel formulation of ingenol mebutate has been recently approved for the treatment of actinic keratosis [15] and fumaric acid esters are used for the management of psoriasis [16,17]. Curcumin is a symmetric α,β-unsaturated β-diketone extracted from *Curcuma longa* L., a tropical Southeast Asian plant used as a spice and in traditional Indian medicine [18]. Currently, there are 17 open clinical trials involving curcumin, of which mainly evaluate the combination of curcumin with other substances used in anticancer therapy [19].

In this paper, we report design and preparation of novel Michael acceptors, fumaric acid diamides **4a**–**f**. We have chosen α,β-unsaturated amides because they are less electrophilic than analogous esters and better Michael acceptors [1]. Based on our previous findings [20,21], one of the amide bonds was achieved with a terminal amino group of primaquine (PQ), while the other with halogenanilines (Figure 1). Sharing the same conjugated C=C-CO system and a benzene ring, our compounds are similar to cinnamic acid (*trans*-3-phenyl-2-propenoic acid) derivatives as well. Taking literature data into account [22,23,24] and our previous experience with PQ derivatives [20,21,25,26,27,28], we have assumed that the designed compounds have a high pharmacological potential. Here we report their synthesis, evaluation of antimicrobial activity on a wide spectrum of bacteria, fungi and viruses, their biofilm eradication ability, and finally, cytostatic activity against several human tumor cell lines.

## 2. Results and Discussion

### 2.1. Chemistry

**Scheme 1:**
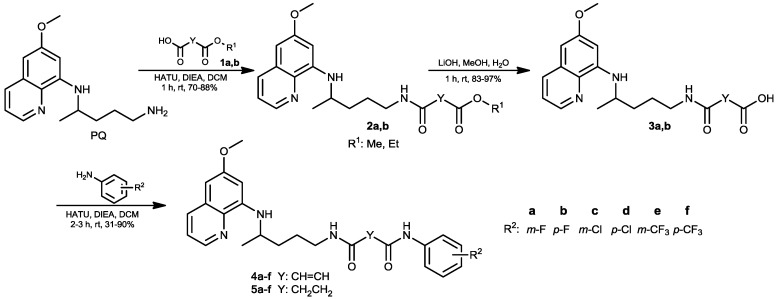
Synthesis of fumardiamides **4a**–**f** and succindiamides **5a**–**f**.

In this paper, we aimed to prepare and biologically evaluate new Michael acceptors **4a**–**f**, asymmetric diamides of fumaric acid. To prove the significance of the conjugated Michael system for activity, we have also prepared a series of analogous compounds without a double bond, e.g., a series of succindiamides **5a**–**f** (compound **5a** is a reduced derivative of **4a**, **5b** of **4b** etc.). In both series of compounds, one of the amide bonds was achieved with the primary amino group of PQ and the other one with a selected halogenaniline. In the first reaction step, mono-ethyl fumarate (**1a**) and mono-methyl succinate (**1b**) were coupled with PQ to give derivatives **2a**,**b** using 1-[bis(dimethylamino)methylene]-1*H*-1,2,3-triazolo[4,5-*b*]pyridinium 3-oxide hexafluorophosphate (HATU) as a coupling reagent, along with *N*,*N*-diisopropylethylamine (DIEA) [29]. However, the transformation of **1a** to carboxylic acid chloride and amidation with PQ gave better yields of product **2a**. Hydrolysis of **2a**,**b** by lithium hydroxide afforded the corresponding acids **3a**,**b**, which were again coupled with halogenanilines in the presence of HATU/DIEA. The following anilines were used: 3-fluoroaniline, 4-fluoroaniline, 3-chloroaniline, 4-chloroaniline, 3-trifluoromethylaniline and 4-trifluoromethylaniline. Scheme 1 shows the synthetic pathway leading to compounds **4** and **5**.

New compounds are fully characterized by MS, IR, ^1^H and ^13^C NMR spectroscopic methods and elemental analyses. Spectral data are consistent with the proposed structures and are given in short in the Materials and Methods and in detail in the Supplementary Material. The presence of carbonyl functional groups in compounds **4** and **5** was indicated by the appearance of strong stretching vibration bands in IR spectra between *ν* 1677 and 1628 (amide I) and 1555 and 1515 cm^–1^ (amide II). PQ residue showed characteristic signals in ^1^H NMR spectra: hydrogen atom CH-15 occurred between *δ* 8.52 and 8.55, methoxy group at *δ* 3.82, the hydrogen attached to chiral carbon (CH-10) as a multiplet at *δ* 3.57–3.65, a methyl group at *δ* 1.19–1.22. NH-1 appeared as a singlet between *δ* 9.98 and 10.75 ppm, NH-6 as a triplet at *δ* 7.86–8.55, while NH-12 as a doublet at *δ* 6.11–6.15 ppm. ^13^C spectra showed characteristic PQ signals at *δ* 54.92–54.98 (methoxy group), 46.19–47.02 (C-10), 38.46–39.23 (C-7), 33.37–33.45 (C-9), 25.70–25.99 (C-8), 20.14–20.21 (C-11) ppm and corresponding signals in aromatic region. Two signals of carbonyl groups in fumardiamides **4a**–**f** appeared between 161.17 and 163.34 ppm, while in succindiamides **5a**–**f** between 170.35 and 171.18 ppm. The other two succinic acid carbons (C-3 and C-4) appeared in the aliphatic region between 30.10 and 31.75, and fumaric acid carbons connected by double bond were located very high between 132.11 and 134.66 ppm. CF_3_ groups in compounds **4e**, **4f**, **5e** and **5f** showed characteristic quartets at 121.32–127.05 with average *J* = 272 Hz, and the closest C-atom to trifluoromethyl quartets at 124–129 ppm with *J* = 31 Hz. *m*-Fluorophenyl derivatives **4a** and **5a** showed doublets at 162 ppm and *p*-fluorophenyl derivatives **4b** and **5b** doublets at 158 ppm (C-atom bearing fluorine) with a similar *J*-coupling constant of 240 Hz, while two neighbouring C-atoms appeared at 105–110 ppm (average *J* = 21 Hz). C-atom substituted with chloro atom appeared at 133 ppm (*m*-Cl) and 127 ppm (*p*-Cl). Chemical structures of new compounds were also supported by mass spectroscopy. Molecular ion peaks corresponding to expected relative molecular masses were obtained for all compounds.

Diamides **4** and **5** were subjected to in silico analysis to evaluate the diversity of the set of compounds against topological polar surface area (TPSA) calculations and relevant drug-like properties: number of atoms, molecular weight (MW), partition coefficient (log *P*), H-bond donor (HBD), H-bond acceptor (HBA) and molecular refractivity (MR). The parameters are calculated with the Chemicalize.org program [30] and presented in Table S1. All compounds are fully in agreement with the Lipinski and Gelovani rules for prospective small molecular drugs (MW ≤ 500, log *P* ≤ 5, number of H-bond donors ≤ 5, number of H-bond acceptors ≤ 10, TPSA < 140 Å^2^, MR within the range of 40 and 130 cm^3^/mol, the number of atoms 20–70), although **4e**,**f** and **5e**,**f** have the highest permitted relative molecular masses.

### 2.2. Biological Evaluation

#### 2.2.1. Antibacterial and Antifungal Activity

The antimicrobial screening was assessed against a panel of Gram-positive bacteria (*S. pneumoniae* MFBF 10373, *S. aureus* ATCC 6538, methicillin-resistant *S. aureus* MRSA 63718, SA 630 and SA 3202, *E. faecalis* ATCC 29212 and three vancomycin-resistant *Enterococci* VRE 342 B, 365 and 725 B, *B. cereus* ATCC 11778, and *B. subtilis* ATCC 6633), Gram-negative species (*P. aeruginosa* ATCC 27853, *E. coli* ATCC 10536, *S. marcescens* ATCC 10905, *P. mirabilis* MFBF 10430, *A. baumannii* MFBF 10913, and *S. enteritidis* MFBF 11945), four *Mycobacterium* strains (*M. tuberculosis* H37Ra, *M. smegmatis* ATCC 700084, *M. kansasii* DSM 44162, and *M. marinum* CAMP 5644) and fungi (two strains of *C. albicans* ATCC 90028 and CCM 8361, *C. krusei* CCM 8271, *C. parapsilosis* CCM 8260 and *A. brasiliensis* ATCC 16404). The results of the antimicrobial screening of fumardiamides **4a**–**f** are presented in Table 1 (the results for inactive fumardiamides and succindiamides **5a**–**f** are not shown). In general, Gram-positive bacteria, especially *S. pneumoniae* and *S. aureus* ATCC 6538 were susceptible to **4b**–**f**. Compounds **4a**,**b** were active against *B. cereus* as well. However, Gram-negative bacterium *A. baumannii* was the most susceptible among all tested microorganisms: all compounds, except *m*-fluoro derivative **4a**, showed selective antimicrobial activity against this bacterium strain. Comparison of *meta* and *para* derivatives, e.g., **a** vs. **b**, **c** vs. **d**, **e** vs. **f** revealed that in the most cases *p*-substituted derivatives were more active than the analogous *m*-derivatives. *p*-Chlorofumardiamide **4d** showed significant activity against three bacterial strains (*S. aureus, S. pneumoniae* and *A. baumannii*), but also against *C. albicans* ATCC 90028, with minimum inhibitory concentration (MIC) values ranging from 6.1 to 12.5 µg/mL. This compound, together with *p*-fluoro and *p*-CF_3_ fumardiamides **4b**,**f** showed antitubercular activity against *M. marinum*, while **4b**,**f** were active against *M. tuberculosis* as well.

#### 2.2.2. Biofilm Eradication Assay

We tested the susceptibility of different bacterial and yeast strains to fumardiamides **4a**–**f** and succindiamides **5a**–**f** by determining minimum biofilm eradication concentrations (MBECs). The results are presented in Table 2. Again, fumardiamides **4** were much more active than the analogous succindiamides **5**. The most active compounds were **4a** and **4b**. Two microorganisms, namely *E. faecalis* and *S. aureus* showed high susceptibility to all fumardiamides, while *E. coli*, *S. pneumoniae* and *P. aeruginosa* were susceptible to five out of six fumardiamides. A high biofilm eradication potential of fumardiamides might be explained by the reaction of Michael acceptors with cysteine thiol, which could prevent disulfide bond formation. It is a well-known fact that cysteine homeostasis impacts biofilm formation and production of extracellular matrix components, as well as folding and stability of extracytoplasmic proteins [31]. They are also crucial for dental plaque formation, autolysis, extracellular DNA release, genetic competence, bacteriocin production and stabilization of outer membrane porin proteins [32].

#### 2.2.3. Antiviral Evaluation

Compounds **4a**–**f** and **5a**–**f** were evaluated against a broad variety of viral infections including herpes simplex viruses, vaccinia virus, adenovirus-2, human coronavirus (229E), vesicular stomatitis virus, coxsackie virus B4, respiratory syncytial virus, para-influenza-3 virus, reovirus-1, sindbis virus, coxsackie virus B4, Punta Toro virus and yellow fever virus. Only fumardiamides with *m*-chloro and *m*-trifluoromethyl aniline residues, namely **4c** and **4e**, showed significant antiviral activity against reovirus-1, sindbis virus and Punta Toro virus with EC_50_ = 3.1–5.5 µM (EC_50_ = concentration required to reduce virus-induced cytopathogenicity by 50%). Compound **4e** was also active against coxsackie virus B4 (EC_50_ = 3.1 µM). However, their selectivity index (SI), e.g., minimum cytotoxicity concentration (MCC) and EC_50_ ratio, was quite low (1.8–3.7). Cytotoxicity and antiviral activity of fumardiamides **4a**–**f** in Vero cell cultures are displayed in Table 3 (data for HEL (human erythroleukemia cell line), Hela (cervical carcinoma cell line) and Madin-Darby Canine Kidney cells (MDCK) cultures are not shown, as well as data for inactive succindiamides **5a**–**f**).

#### 2.2.4. Cytostatic Activity

To gain insight into cytotoxicity of newly synthesized compounds **4a**–**f** and **5a**–**f**, their cytostatic activity was evaluated in vitro against a panel of nine different human cancer cell lines, representing various solid tumor types including pancreatic adenocarcinoma (Capan-1), chronic myeloid leukemia (Hap1), colorectal carcinoma (HCT-116), lung carcinoma (NCI-H460), acute lymphoblastic leukemia (DND-41), acute myeloid leukemia (HL-60), chronic myeloid leukemia (K-562), multiple myeloma (MM.1S) and non-Hodgkin lymphoma (Z-138). Succindiamides **5a**–**f** were completely inactive, while free fumardiamides showed activity towards the selected tumor cell lines (Table 4). Fumardiamide **4a** with *m*-fluoroaniline moiety showed cytostatic activity against all tested cell lines, with IC_50_ values from 5.7 to 31.2 μM (IC_50_ = the lowest concentration resulting in 50% growth inhibition). DND-41 cell line was susceptible to all three *m*-substituted derivatives, e.g., **4a**, **4c** and **4f** (IC_50_ values between 6.7 and 8.9 μM). HL-60 and Z-138 cell lines were also susceptible to compound **4a** (IC_50_ values 5.7 and 8.4 μM, respectively).

#### 2.2.5. Interaction with Glutathione (GSH)

Glutathione (GSH) is an important compound present in most mammalian cells, a tripeptide, with the central amino acid cysteine bearing thiol group, which scavenges carcinogenic compounds by conjugate addition and protects from oxidative damage [1]. In the drug development process, it is usual to evaluate the Michael acceptor–GSH interaction [33]. That is why the final step in our research was to study the interaction of fumarmides with GSH. Fumardiamide **4b** (1.25 μM) was incubated with GSH (125 μM) in ammonium formate buffer (pH = 7.4) containing 10% acetonitrile at 37 °C for 216 h. The MS analysis confirmed the consumption of **4b** with GSH. However, the rate of GSH addition was slow and incomplete, as only 18.3% of **4b** reacted in the monitored period (See Supporting Material).

## 3. Materials and Methods

### 3.1. Chemistry

#### 3.1.1. Materials and General Methods

Melting points were determined on an SMP3 apparatus (Barloworld Scientific, UK) in open capillaries and were uncorrected. IR spectra were recorded on Spectrum One FT-IR (Perkin-Elmer, UK) and UV-Vis spectra on Lambda 20 double-beam spectrophotometers (Perkin-Elmer, UK). NMR ^1^H and ^13^C spectra were recorded at 25 °C on an NMR Avance 600 spectrometer (Bruker, Germany) at 300.13 or 600.13 and 75.47 or 150.9 MHz for ^1^H and ^13^C nuclei, respectively. Chemical shifts (*δ*) were reported in parts per million (ppm) relative to tetramethylsilane in the ^1^H and the dimethyl sulfoxide residual peak as a reference in the ^13^C NMR spectra (39.51 ppm). Coupling constants (*J*) were reported in hertz (Hz). Mass spectra were collected on an HPLC-MS/MS instrument (HPLC, Agilent Technologies 1200 Series; MS, Agilent Technologies 6410 Triple Quad) using electrospray ionization in positive mode. Elemental analyses were performed on a CHNS LECO analyzer (LECO Corporation, USA). All compounds were routinely checked by thin-layer chromatography (TLC) with Merck silica gel 60F-254 glass plates using appropriate solvent systems. Spots were visualized by short-wave UV light and iodine vapour. Column chromatography was performed on silica gel 0.063–0.200 mm. All chemicals and solvents were of analytical grade and purchased from commercial sources. PQ diphosphate, 4-methoxy-4-oxobutanoic acid (mono-methyl succinate), (*E*)-4-ethoxy-4-oxobut-2-enoic acid (mono-ethyl fumarate), 3-fluoroaniline, 4-fluoroaniline, 3-chloroaniline, 4-chloroaniline, 3-trifluoromethylaniline, 4-trifluoromethylaniline, DIEA, and HATU were purchased from Sigma-Aldrich. PQ was prepared from PQ diphosphate prior to use. All reactions with PQ were run light protected.

#### 3.1.2. General Procedure for the Synthesis of Esters **2a**,**b**

PQ-succinamide and fumaramide monoesters **2a**,**b** were prepared by condensation of PQ base with mono-methyl succinate (**1a**) or mono-ethyl fumarate (**1b**), following the previously described procedure [29].

#### 3.1.3. General Procedure for the Synthesis of Carboxylic Acids **3a**,**b**

Carboxylic acids **3a**,**b** were prepared by hydrolysis of esters **2a**,**b** with lithium hydroxide following the previously described procedure [29].

#### 3.1.4. General Procedure for the Synthesis of Fumardiamides **4a**–**f** and Succindiamides **5a**–**f**

A solution of 0.27 mmol of **3a** or **3b**, 0.068 g (0.54 mmol) DIEA and 0.103 g (0.27 mmol) HATU in 6 mL of dichloromethane was stirred at room temperature. After 10 min, 0.297 mmol of the corresponding halogenaniline was added. The reaction mixture was stirred for 2–3 h at room temperature, evaporated under reduced pressure, dissolved in 8 mL ethyl acetate and extracted 3 times with water. The organic layer was dried over sodium sulfate, filtered and evaporated under reduced pressure.

*(2E)-N-(3-Fluorophenyl)-N′-{4-[(6-methoxyquinolin-8-yl)amino]pentyl}but-2-enediamide (**4a**).* From the reaction of 0.096 g acid **3a** and 0.033 g (0.297 mmol) 3-fluoroaniline and after purification by column chromatography (mobile phase dichloromethane/methanol 9.5:0.5) and crystallization from ether, 0.041 g (34%) of **4a** was obtained; mp 203–204 °C; IR (ATR): *ν*_max_ 3388, 3319, 3269, 3080, 2961, 2935, 2866, 1630, 1554, 1520, 1452, 1387, 1334, 1201, 1158, 782, 680 cm^–1^; ^1^H NMR (DMSO-*d*_6_) *δ* 10.63 (s, 1H), 8.55–8.53 (dd, 1H, *J* = 1.6, 4.2), 8.52 (t, 1H, *J* = 5.4), 8.09–8.06 (dd, 1H, *J* = 1.5, 8.3), 7.69 (d, 2H, *J* = 11.7), 7.45–7.41 (m, 1H), 7.39–7.36 (m, 2H), 7.06–7.96 (m, 2H), 6.96–6.92 (m, 1H), 6.47 (d, 1H, *J* = 2.4), 6.28 (d, 1H, *J* = 2.4), 6.15 (d, 1H, *J* = 8.8), 3.82 (s, 3H), 3.70–3.61 (m, 1H), 3.24–3.18 (m, 2H), 1.74–1.64, 1.63–1.52 (2m, 4H), 1.22 (d, 3H, *J* = 6.3); ^13^C NMR (DMSO-*d*_6_) *δ* 163.25, 162.62, 162.09 (d, *J* = 242.0), 159.00, 144.63, 144.23, 140.50 (d, *J* = 11.5), 134.79, 134.52, 134.40, 132.28, 130.50 (d, *J* = 9.5), 129.57, 122.09, 115.14, 110.28, 106.13 (d, *J* = 27.2), 96.14, 91.62, 54.96, 46.97, 38.95, 33.45, 25.77, 20.21; MS/MS *m/z* 451.1 (M + 1)^+^; Anal. Calcd. for C_25_H_27_FN_4_O_3_: C, 66.65; H, 6.04; N, 12.44. Found: C, 66.32; H, 6.30; N, 12.49.

*(2E)-N-(4-Fluorophenyl)-N′-{4-[(6-methoxyquinolin-8-yl)amino]pentyl}but-2-enediamide (**4b**).* From the reaction of 0.096 g acid **3a** and 0.033 g (0.297 mmol) 4-fluoroaniline and after purification by column chromatography (mobile phase dichloromethane/methanol 9.5:0.5) and crystallization from ether, 0.071 g (58%) of **4b** was obtained; mp 226–227 °C; IR (ATR): *ν*_max_ 3386, 3294, 3072, 2963, 2928, 2863, 1635, 1548, 1513, 1452, 1391, 1330, 1212, 1160, 1051, 973, 829, 673 cm^–1^; ^1^H NMR (DMSO-*d*_6_) *δ* 10.49 (s, 1H), 8.55–8.53 (dd, 1H, *J* = 1.6, 4.2), 8.52 (t, 1H, *J* = 5.4), 8.09–8.06 (dd, 1H, *J* = 1.5, 8.3), 7.72–7.68 (dd, 2H, *J* = 5.0, *J* = 8.9), 7.45–7.41 (m, 1H), 7.18 (t, 2H, *J* = 8.8), 7.05–6.93 (m, 2H), 6.47 (d, 1H, *J* = 2.1), 6.28 (d, 1H, *J* = 2.1), 6.15 (d, 1H, *J* = 8.8), 3.82 (s, 3H), 3.69–3.61 (m, 1H), 3.24–3.18 (m, 2H), 1.74–1.64, 1.63–1.52 (2m, 4H), 1.22 (d, 3H, *J* = 6.3); ^13^C NMR (DMSO-*d*_6_) *δ* 163.34, 162.21, 159.00, 158.26 (d, *J* = 240.0), 144.63, 144.23, 135.22, 134.79, 134.53, 133.98, 132.52, 129.58, 122.09, 121.05 (d, *J* = 7.6), 115.44 (d, *J* = 21.9), 96.14, 91.62, 54.96, 46.97, 38.95, 33.45, 25.79, 20.21; MS/MS *m/z* 451.1 (M + 1)^+^; Anal. Calcd. for C_25_H_27_FN_4_O_3_: C, 66.65; H, 6.04; N, 12.44. Found: C, 66.47; H, 6.38; N, 12.35.

*(2E)-N-(3-Chlorophenyl)-N′-{4-[(6-methoxyquinolin-8-yl)amino]pentyl}but-2-enediamide (**4c**).* From the reaction of 0.096 g acid **3a** and 0.038 g (0.297 mmol) 3-chloroaniline and after purification by column chromatography (mobile phase dichloromethane/methanol 9.5:0.5) and crystallization from ether, 0.053 g (42%) of **4c** was obtained; mp 187–188 °C; IR (ATR): *ν*_max_ 3381, 3298, 3068, 2959, 2928, 2863, 1635, 1591, 1521, 1465, 1419, 1386, 1331, 1210, 1163, 976, 821, 783, 670 cm^–1^; ^1^H NMR (DMSO-*d*_6_) *δ* 10.59 (s, 1H), 8.54–8.53 (dd, 1H, *J* = 1.5, 4.1), 8.50 (t, 1H, *J* = 5.5), 8.08–8.06 (dd, 1H, *J* = 1.4, 8.2), 7.90 (s, 1H), 7.51 (d, 1H, *J* = 8.1), 7.43–7.41 (m, 1H), 7.37 (t, H, *J* = 8.1), 7.16–7.14 (dd, 1H, *J* = 1.2, *J* = 8.0), 7.00 (q, 2H, *J* = 15.1), 6.47 (d, 1H, *J* = 2.4), 6.28 (d, 1H, *J* = 2.3), 6.14 (d, 1H, *J* = 8.8), 3.82 (s, 3H), 3.67–3.63 (m, 1H), 3.23–3.20 (m, 2H), 1.73–1.67, 1.63–1.53 (2m, 4H), 1.22 (d, 3H, *J* = 6.3); ^13^C NMR (DMSO-*d*_6_) *δ* 163.21, 162.60, 158.97, 144.60, 144.19, 140.19, 134.74, 134.50, 134.41, 133.10, 132.19, 130.48, 129.54, 123.45, 122.03, 118.74, 117.72, 96.11, 91.63, 54.93, 46.98, 39.23, 33.44, 25.72, 20.18; MS/MS *m/z* 467.0 (M+1)^+^; Anal. Calcd. for C_25_H_27_ClN_4_O_3_: C, 64.30; H, 5.83; N, 12.00. Found: C, 64.21; H, 6.05; N, 11.78.

*(2E)-N-(4-Chlorophenyl)-N′-{4-[(6-methoxyquinolin-8-yl)amino]pentyl}but-2-enediamide (**4d**).* From the reaction of 0.096 g acid **3a** and 0.038 g (0.297 mmol) 4-chloroaniline and after purification by column chromatography (mobile phase cyclohexane/ethyl acetate/methanol 3:1:0.5) and crystallization from ether, 0.062 g (49%) of **4d** was obtained; mp 223–226 °C; IR (ATR): *ν*_max_ 3381, 3289, 3071, 2959, 2931, 2864, 1640, 1526, 1452, 1388, 1331, 1210, 1163, 1094, 1049, 973, 822, 787, 686, 631, 507 cm^–1^; ^1^H NMR (DMSO-*d*_6_) *δ* 10.56 (s, 1H), 8.55–8.53 (dd, 1H, *J* = 1.7, 4.2), 8.50 (t, 1H, *J* = 5.6), 8.09–8.06 (dd, 1H, *J* = 1.6, 8.3), 7.72–7.68 (m, 2H), 7.45–7.37 (m, 3H), 7.06–6.94 (m, 2H), 6.47 (d, 1H, *J* = 2.5), 6.27 (d, 1H, *J* = 2.4), 6.14 (d, 1H, *J* = 8.8), 3.82 (s, 3H), 3.69–3.60 (m, 1H), 3.24–3.18 (m, 2H), 1.74–1.64, 1.63–1.51 (2m, 4H), 1.22 (d, 3H, *J* = 6.3); ^13^C NMR (DMSO-*d*_6_) *δ* 163.29, 162.43, 159.00, 144.63, 144.23, 137.76, 134.79, 134.52, 134.21, 132.38, 129.57, 128.75, 127.36, 122.09, 120.84, 96.14, 91.61, 54.96, 46.97, 38.82, 33.44, 25.77, 20.21; MS/MS *m/z* 467.0 (M+1)^+^; Anal. Calcd. for C_25_H_27_ClN_4_O_3_: C, 64.30; H, 5.83; N, 12.00. Found: C, 64.21; H, 5.56; N, 11.83.

*(2E)-N′-{4-[(6-Methoxyquinolin-8-yl)amino]pentyl}-N-[3-(trifluoromethyl)phenyl]but-2-enediamide (**4e**).* From the reaction of 0.096 g acid **3a** and 0.048 g (0.297 mmol) 3-trifluoroaniline and after purification by column chromatography (mobile phase dichloromethane/methanol 9.5:0.5) after crystallization from acetone/water, 0.042 g (31%) of **4e** was obtained; mp 149–150 °C; IR (ATR): *ν*_max_ 3399, 3357, 3282, 3094, 2960, 2935, 2867, 1651, 1621, 1563, 1526, 1452, 1388, 1331, 1168, 1122, 973, 893, 788, 694 cm^–1^; ^1^H NMR (DMSO-*d*_6_) *δ* 10.75 (s, 1H), 8.55–8.52 (m, 2H), 8.18 (s, 1H), 8.08–8.06 (dd, 1H, *J* = 1.1, 8.2), 7.83 (d, 1H), 7.59 (t, 1H, *J* = 7.9), 7.45–7.42 (m, 2H), 7.00–6.98 (q, 2H, *J* = 15.1), 6.47 (d, 1H, *J* = 2.4), 6.28 (d, 1H, *J* = 2.0), 6.15 (d, 1H, *J* = 8.7), 3.82 (s, 3H), 3.67–3.63 (m, 1H), 3.23–3.20 (m, 2H), 1.73–1.67, 1.63–1.53 (2m, 4H), 1.22 (d, 3H, *J* = 6.3); ^13^C NMR (DMSO-*d*_6_) *δ* 163.19, 162.81, 158.99, 144.62, 144.21, 139.54, 134.77, 134.55, 134.52, 132.15, 130.09, 129.56, 129.82–129.19 (q, *J* = 31.7), 126.73–121.32 (q, *J* = 273.3), 122.88, 122.07, 120.09, 115.33, 96.13, 91.62, 54.95, 46.19, 39.23, 33.45, 25.76, 20.20; MS/MS *m/z* 501.1 (M+1)^+^; Anal. Calcd. for C_26_H_27_F_3_N_4_O_3_: C, 62.39; H, 5.44; N, 11.19. Found: C, 62.25; H, 5.76; N, 11.08.

*(2E)-N′-{4-[(6-Methoxyquinolin-8-yl)amino]pentyl}-N-[4-(trifluoromethyl)phenyl]but-2-enediamide (**4f**).* From the reaction of 0.096 g acid **3a** and 0.048 g (0.297 mmol) 4-trifluoroaniline and after crystallization from ether, 0.046 g (34%) of **4f** was obtained; mp 189–191 °C; IR (ATR): *ν*_max_ 3387, 3309, 3071, 2963, 2932, 1636, 1527, 1457, 1417, 1390, 1328, 1213, 1166, 1122, 1065, 970, 832, 681 cm^–1^; ^1^H NMR (DMSO-*d*_6_) *δ* 10.75 (s, 1H), 8.54–8.53 (m, 1H), 8.51 (t, 1H, *J* = 5.3), 8.07 (d, 1H, *J* = 8.2), 7.88 (d, 2H, *J* = 8.5), 7.71 (d, 2H, *J* = 8.5), 7.43–7.41 (m, 1H), 7.07–6.99 (q, 2H, *J* = 15.1), 6.47 (d, 1H, *J* = 2.2), 6.28 (d, 1H, *J* = 2.2), 6.14 (d, 1H, *J* = 8.7), 3.82 (s, 3H), 3.67–3.63 (m, 1H), 3.23–3.20 (dd, 2H, *J* = 6.1, 12.1), 2.59 (t, 2H, *J* = 7.0), 1.73–1.67, 1.61–1.53 (2m, 4H), 1.22 (d, 3H, *J* = 6.3); ^13^C NMR (DMSO-*d*_6_) *δ* 161.17, 162.83, 158.97, 144.60, 144.17, 142.28, 134.72, 134.66, 134.50, 132.11, 129.53, 126.95–121.55 (q, *J* = 271.6), 126.11–126.04 (q, *J* = 3.0), 124.02–123.39 (q, *J* = 31.7), 122.02, 119.26, 96.10, 91.63, 54.92, 46.96, 38.80, 33.43, 25.70, 20.17; MS/MS *m/z* 501.1 (M+1)^+^; Anal. Calcd. for C_26_H_27_F_3_N_4_O_3_: C, 62.39; H, 5.44; N, 11.19. Found: C, 62.17; H, 5.61; N, 11.40.

*N-(3-Fluorophenyl)-N′-{4-[(6-methoxyquinolin-8-yl)amino]pentyl}butanediamide (**5a**).* From the reaction of 0.097 g acid **3b** and 0.033 g (0.297 mmol) 3-fluoroaniline and after purification by column chromatography (mobile phase dichloromethane/methanol 9.5:0.5), 0.077 g (63%) of **5a** was obtained; mp 140–142 °C; IR (ATR): *ν*_max_ 3391, 3283, 3145, 3080, 2959, 2926, 1744, 1646, 1615, 1555, 1507, 1387, 1224, 1202, 1167, 1157, 838, 815, 786, 681 cm^–1^; ^1^H NMR (DMSO-*d*_6_) *δ* 10.13 (s, 1H), 8.54–8.53 (dd, 1H, *J* = 1.7, 4.2), 8.08–8.06 (dd, 1H, *J* = 1.6, 8.3), 7.86 (t, 1H, *J* = 5.6), 7.60–7.58 (m, 1H), 7.43–7.41 (m, 1H), 7.31–7.27 (m, 2H), 6.85–6.81 (m, 1H), 6.47 (d, 1H, *J* = 2.5), 6.26 (d, 1H, *J* = 2.5), 6.11 (d, 1H, *J* = 8.7), 3.82 (s, 3H), 3.64–3.59 (m, 1H), 3.10–3.03 (m, 2H), 2.55 (t, 2H, *J* = 7.2), 2.37 (t, 2H, *J* = 7.2), 1.67–1.63, 1.55–1.45 (2m, 4H), 1.19 (d, 3H, *J* = 6.3); ^13^C NMR (DMSO-*d*_6_) *δ* 170.86, 162.10 (d, *J* = 240.1), 158.98, 144.61, 144.19, 141.03 (d, *J* = 13.1), 134.75, 134.51, 130.19 (d, *J* = 9.0), 129.55, 122.05, 114.55, 109.22 (d, *J* = 20.1), 105.61, 96.07, 91.59, 54.94, 46.99, 38.46, 33.38, 31.75, 30.16, 25.95, 20.15; MS/MS *m/z* 453.3 (M+1)^+^; Anal. Calcd. for C_25_H_29_FN_4_O_3_: C, 66.35; H, 6.46; N, 12.38. Found: C, 66.25; H, 6.50; N, 12.20.

*N-(4-Fluorophenyl)-N′-{4-[(6-methoxyquinolin-8-yl)amino]pentyl}butanediamide (**5b**).* From the reaction of 0.097 g acid **3b** and 0.033 g (0.297 mmol) 4-fluoroaniline and after purification by column chromatography (mobile phase dichloromethane/methanol 9.5:0.5) and crystallization from ether, 0.098 g (80%) of **5b** was obtained; mp 118–120 °C; IR (ATR): *ν*_max_ 3391, 3283, 3145, 3080, 2959, 2926, 1744, 1646, 1615, 1555, 1507, 1387, 1224, 1202, 1167, 1157, 838, 815, 786, 681 cm^–1^; ^1^H NMR (DMSO-*d*_6_) *δ* 9.98 (s, 1H), 8.55–8.53 (dd, 1H, *J* = 1.6, 4.2), 8.09–8.06 (dd, 1H, *J* = 1.5, 8.3), 7.87 (t, 1H, *J* = 5.5), 7.62–7.57 (m, 2H), 7.45–7.40 (m, 1H), 7.14–7.08 (m, 2H), 6.47 (d, 1H, *J* = 2.4), 6.26 (d, 1H, *J* = 2.4), 6.12 (d, 1H, *J* = 8.8), 3.82 (s, 3H), 3.65–3.57 (m, 1H), 3.10–3.04 (m, 2H), 2.54 (t, 2H, *J* = 7.1), 2.37 (t, 2H, *J* = 7.0), 1.69–1.59, 1.58–1.43 (2m, 4H), 1.19 (d, 3H, *J* = 6.3); ^13^C NMR (DMSO-*d*_6_) *δ* 170.96, 170.35, 159.00, 157.72 (d, *J* = 246.3), 144.63, 144.22, 135.76, 134.79, 134.52, 129.57, 122.09, 120.53 (d, *J* = 7.8), 115.14 (d, *J* = 22.1), 96.09, 91.58, 54.97, 47.00, 38.48, 33.39, 31.66, 30.34, 25.99, 20.17; MS/MS *m/z* 453.4 (M+1)^+^; Anal. Calcd. for C_25_H_29_FN_4_O_3_: C, 66.35; H, 6.46; N, 12.38. Found: C, 66.53, H, 6.29; N, 12.55.

*N-(3-Chlorophenyl)-N′-{4-[(6-methoxyquinolin-8-yl)amino]pentyl}butanediamide (**5c**).* From the reaction of 0.097 g acid **3b** and 0.038 g (0.297 mmol) 3-chloroaniline and after purification by column chromatography (mobile phase cyclohexane/ethyl acetate/methanol 3:1:0.5) and crystallization from ether, 0.062 g (49%) of **5c** was obtained; mp 156–158 °C; IR (ATR): *ν*_max_ 3390, 3287, 3242, 3180, 3102, 3074, 2962, 2922, 2856, 1738, 1650, 1612, 1591, 1572, 1539, 1516, 1422, 1388, 1202, 1066, 816, 787, 698, 682 cm^–1^; ^1^H NMR (DMSO-*d*_6_) *δ* 10.11 (s, 1H), 8.54–8.53 (dd, 1H, *J* = 1.6, 4.2), 8.08–8.06 (dd, 1H, *J* = 1.6, 8.3), 7.86 (t, 1H, *J* = 5.5), 7.81 (t, 1H, *J* = 2.0), 7.43–7.41 (m, 2H), 7.31–7.28 (m, 1H), 7.07–7.05 (m, 1H), 6.47 (d, 1H, *J* = 2.5), 6.25 (d, 1H, *J* = 2.5), 6.11 (d, 1H, *J* = 8.2), 3.82 (s, 3H), 3.63–3.59 (m, 1H), 3.10–3.03 (m, 2H), 2.55 (t, 2H, *J* = 7.1), 2.37 (t, 2H, *J* = 7.2), 1.67–1.63, 1.56–1.44 (2m, 4H), 1.19 (d, 3H, *J* = 6.3); ^13^C NMR (DMSO-*d*_6_) *δ* 170.89, 170.85, 158.98, 144.60, 144.19, 140.73, 134.77, 134.49, 132.97, 130.29, 129.55, 122.51, 122.06, 118.31, 117.19, 96.08, 91.59, 54.95, 46.99, 38.46, 33.38, 31.74, 30.16, 25.96, 20.16; MS/MS *m/z* 469.2 (M+1)^+^; Anal. Calcd. for C_25_H_29_ClN_4_O_3_: C, 64.03; H, 6.23; N, 11.95. Found: C, 63.91; H, 6.33; N, 11.70.

*N-(4-Chlorophenyl)-N′-{4-[(6-methoxyquinolin-8-yl)amino]pentyl}butanediamide (**5d**).* From the reaction of 0.097 g acid **3b** and 0.038 g (0.297 mmol) 3-chloroaniline and after purification by column chromatography (mobile phase dichloromethane/methanol 9.5:0.5) and crystallization from ether, 0.076 g (60%) of **5d** was obtained; mp 152–153 °C; IR (ATR): *ν*_max_ 3390, 3287, 3242, 3180, 3102, 3074, 2962, 2922, 2856, 1738, 1650, 1612, 1591, 1572, 1539, 1516, 1422, 1388, 1202, 1066, 816, 787, 698, 682 cm^–1^; ^1^H NMR (DMSO-*d*_6_) *δ* 10.07 (s, 1H), 8.55–8.53 (dd, 1H, *J* = 1.5, 4.2), 8.10–8.07 (dd, 1H, *J* = 1.4, 8.3), 7.88 (t, 1H, *J* = 5.4), 7.61 (d, 2H, *J* = 8.9), 7.45–7.41 (m, 1H), 7.32 (d, 2H, *J* = 8.9), 6.48 (d, 1H, *J* = 2.3), 6.26 (d, 1H, *J* = 2.3), 6.13 (bs, 1H), 3.81 (s, 3H), 3.63–3.57 (m, 1H), 3.09–3.03 (m, 2H), 2.55 (t, 2H, *J* = 7.4), 2.39 (t, 2H, *J* = 7.0), 1.70–1.59, 1.58–1.43 (2m, 4H), 1.19 (d, 3H, *J* = 6.3); ^13^C NMR (DMSO-*d*_6_) *δ* 170.92, 170.64, 159.01, 144.55, 144.18, 138.28, 134.89, 134.42, 129.59, 128.51, 126.33, 122.09, 120.37, 96.19, 91.63, 54.98, 47.02, 38.47, 33.37, 31.73, 30.23, 25.98, 20.15 (11); MS/MS *m/z* 469.3 (M+1)^+^; Anal. Calcd. for C_25_H_29_ClN_4_O_3_: C, 64.03; H, 6.23; N, 11.95. Found: C, 64.35; H, 6.57; N, 12.13.

*N′-{4-[(6-Methoxyquinolin-8-yl)amino]pentyl}-N-[3-(trifluoromethyl)phenyl]butanediamide (**5e**).* From the reaction of 0.097 g acid **3b** and 0.048 g (0.297 mmol) 3-trifluoromethylaniline and after purification by column chromatography (mobile phase dichloromethane/methanol 9.5:0.5) and crystallization from ether, 0.122 g (90%) of **5e** was obtained; mp 146–149 °C; IR (ATR): *ν*_max_ 3394, 3302, 3263, 3216, 3164, 3097, 2963, 2928, 2859, 1651, 1617, 1562, 1518, 1450, 1389, 1331, 1265, 1173, 1127, 1064, 894, 816 cm^–1^; ^1^H NMR (DMSO-*d*_6_) *δ* 10.26 (s, 1H), 8.54–8.53 (dd, 1H, *J* = 1.6, 4.2), 8.11 (s, 1H), 8.08–8.06 (dd, 1H, *J* = 1.6, 8.3), 7.88 (t, 1H, *J* = 5.5), 7.74 (d, 1H, *J* = 8.1), 7.52 (t, 1H, *J* = 8.0), 7.43–7.41 (m, 1H), 7.36 (d, 1H, *J* = 7.8), 6.47 (d, 1H, *J* = 2.5), 6.25 (d, 1H, *J* = 2.5), 6.11 (d, 1H, *J* = 8.7), 3.82 (s, 3H), 3.63–3.58 (m, 1H), 3.10–3.04 (m, 2H), 2.57 (t, 2H, *J* = 7.2), 2.41 (t, 2H, *J* = 7.2), 1.67–1.63, 1.56–1.44 (2m, 4H), 1.19 (d, 3H, *J* = 6.3); ^13^C NMR (DMSO-*d*_6_) *δ* 171.11, 170.84, 158.98, 144.61, 144.19, 140.03, 134.75, 134.50, 129.83, 129.55, 129.67–129.04 (q, *J* = 31.9), 126.81–121.40 (q, *J* = 269.8), 122.33, 122.05, 119.14, 114.88, 96.06, 91.58, 54.94, 46.98, 38.46, 33.38, 31.72, 30.11, 25.96, 20.14; MS/MS *m/z* 503.3 (M+1)^+^; Anal. Calcd. for C_26_H_29_F_3_N_4_O_3_: C, 62.14; H, 5.82; N, 11.15. Found: C, 62.25; H, 5.99; N, 11.08.

*N′-{4-[(6-Methoxyquinolin-8-yl)amino]pentyl}-N-[4-(trifluoromethyl)phenyl]butanediamide (**5f**).* From the reaction of 0.097 g acid **3b** and 0.048 g (0.297 mmol) 4-trifluoromethylaniline and after purification by column chromatography (mobile phase dichloromethane/methanol 9.5:0.5), 0.068 g (50%) of **5f** was obtained; mp 163–165 °C; IR (ATR): *ν*_max_ 3387, 3287, 3256, 3198, 3123, 3071, 2963, 2925, 2859, 1652, 1614, 1547, 1515, 1452, 1419, 1389, 1327, 1264, 1169, 1124, 1064, 846, 784 cm^–1^; ^1^H NMR (DMSO-*d*_6_) *δ* 10.31 (s, 1H), 8.54–8.52 (dd, 1H, *J* = 1.5, 4.2), 8.09–8.06 (dd, 1H, *J* = 1.5, 8.3), 7.89 (t, 1H, *J* = 5.3), 7.79 (d, 2H, *J* = 8.5), 7.63 (d, 2H, *J* = 8.7), 7.44–7.40 (m, 1H), 6.47 (d, 1H, *J* = 2.4), 6.25 (d, 1H, *J* = 2.4), 6.11 (d, 1H, *J* = 8.7), 3.82 (s, 3H), 3.65–3.57 (m, 1H), 3.09–3.04 (m, 2H), 2.59 (t, 2H, *J* = 7.0), 2.40 (t, 2H, *J* = 7.0), 1.71–1.58, 1.57–1.43 (2m, 4H), 1.19 (d, 3H, *J* = 6.2); ^13^C NMR (DMSO-*d*_6_) *δ* 171.18, 170.86, 158.99, 144.61, 144.20, 142.83, 134.77, 134.51, 129.55, 125.95, 127.05–121.68 (q, *J* = 274.2), 123.15-122.52 (q, *J* = 28.2), 122.06, 118.69, 96.08, 91.59, 54.95, 46.99, 38.47, 33.38, 31.79, 30.10, 25.96, 20.14; MS/MS *m/z* 503.3 (M+1)^+^; Anal. Calcd. for C_26_H_29_F_3_N_4_O_3_: C, 62.14; H, 5.82; N, 11.15. Found: C, 62.39; H, 5.76; N, 11.41.

### 3.2. Biological Evaluation

#### 3.2.1. In Vitro Antibacterial Susceptibility Assay (MIC Determination)

(a) *Staphylococci* and *Enterococci*: The synthesized compounds were evaluated for in vitro antibacterial activity against representatives of multidrug-resistant bacteria and clinical isolates of methicillin-resistant *Staphylococcus aureus* (MRSA) 63718, SA 630 and SA 3202, that were obtained from the National Institute of Public Health (Prague, Czech Republic). *S. aureus* ATCC 29213 was used as a reference and quality control strain. In addition, all the compounds were tested for their activity against vancomycin-susceptible *Enterococcus faecalis* ATCC 29212 as a reference strain and three isolates from American crows of vanA-carrying vancomycin-resistant *E. faecalis* (VRE) 342B, 368 and 725B [34]. Ampicillin (Amp) and ciprofloxacin (CIP) (Sigma-Aldrich, St. Louis, MO, USA) were used as standards. Prior to testing, each strain was passaged onto nutrient agar (Oxoid, Basingstoke, UK) with 5% of bovine blood, and bacterial inocula were prepared by suspending a small portion of the bacterial colony in sterile phosphate-buffered saline (pH 7.2–7.3). The cell density was adjusted to 0.5 McFarland units using a densitometer (Densi-La-Meter, LIAP, Riga, Latvia). This inoculum was diluted to reach the final concentration of bacterial cells 5 × 10^5^ CFU/mL in the wells. The compounds were dissolved in DMSO (Sigma-Aldrich, St. Louis, MO, USA), and the final concentration of DMSO in the Cation Adjusted Mueller-Hinton (CaMH) broth (Oxoid) for *Staphylococci* or brain-heart infusion for *Enterococci* did not exceed 2.5% of the total solution composition. The final concentrations of the evaluated compounds ranged from 256 to 0.008 μg/mL. The broth dilution micro-method, modified according to NCCLS (National Committee for Clinical Laboratory Standards) guidelines [35] in MH broth for *Staphylococcus* strains and CaMH for *Enterococcus* strains, was used to determine MIC. Drug-free controls, sterility controls, and controls consisting of MH and CaMH broths and DMSO alone were included. The determination of results was performed visually after 24 h of static incubation in darkness at 37 °C in an aerobic atmosphere.

(b) Other Gram-positive (*Streptococcus pneumoniae* MFBF 10373, *Bacillus cereus* ATCC 11778, and *Bacillus subtilis* ATCC 6633) and Gram-negative bacteria species (*Pseudomonas aeruginosa* ATCC 27853, *Escherichia coli* ATCC 10536, *Serratia marcescens* ATCC 10905, *Proteus mirabilis* MFBF 10430, *Acinetobacter baumannii* MFBF 10913, and *Salmonella enteritidis* MFBF 11945): Serial microdilution broth assay was used to determine MIC of compounds **4a**–**f** and **5a**–**f** [36]. Cell suspensions were prepared from stock cultures using phosphate-buffered saline (PBS) (Gibco Laboratories, USA) pH 7.4 and adjusted to 0.5 McFarland units using a nephelometer (ATB 1550, BioMérieux, France). Stock solutions of the tested compounds were prepared in DMSO (10 mg/mL). Testing was performed using serial dilution in microtiter flat-bottom 96-well plates with compounds ranging from 100 to 0.78125 µg/mL. After inoculation (10^7^ CFU/mL) and incubation (18 h, 35 °C, aerobically in darkness), MICs for bacterial species were determined by addition of 0.5 mg/mL TTC (2,3,5-triphenyl-2*H*-tetrazolium chloride). The absorbance was recorded at 540 nm. As a positive quality control (susceptibility of strains) tetracycline hydrochloride (TC) (Sigma-Aldrich) was added into wells. MICs were determined as non-linear regression using GraphPad Prism as the lowest concentrations resulting in 50% growth inhibition of growth in comparison to control.

(c) *Mycobacteria: Mycobacterium tuberculosis* H37Ra ATCC 25177 was grown in Middlebrook broth (MB), supplemented with Oleic-Albumin-Dextrose-Catalase (OADC) supplement (Difco, Lawrence, KS, USA) and mycobactin J (2 μg/mL). At log phase growth, a culture sample (10 mL) was centrifuged at 15,000 rpm/20 min using a bench-top centrifuge (MPW-65R, MPW Med Instruments, Poland). Following removal of the supernatant, the pellet was washed in fresh Middlebrook 7H9GC broth and re-suspended in fresh, ODAC-supplemented MB (10 mL). The turbidity was adjusted to match McFarland standard No. 1 (3 × 10^8^ CFU) with MB broth. A further 1:20 dilution of the culture was then performed in MB broth. The antimicrobial susceptibility of *M. tuberculosis* was investigated in a 96-well plate format. In these experiments, sterile deionised water (300 µl) was added to all outer-perimeter wells of the plates to minimize evaporation of the medium in the test wells during incubation. Each evaluated compound (100 µL) was incubated with *M. tuberculosis* (100 µl). Dilutions of each compound were prepared in duplicate. For all synthesized compounds, final concentrations ranged from 1000 μg/mL to 8 μg/mL. All compounds were dissolved in DMSO, and subsequent dilutions were made in supplemented MB. The plates were sealed with parafilm and incubated at 37 °C for 7 days. Following incubation, 10% of alamarBlue (Difco, Lawrence, KS, USA) was mixed into each well, and readings at 570 nm and 600 nm were taken, initially for background subtraction and subsequently after 24 h reincubation. The background subtraction is necessary for strongly coloured compounds, where the colour may interfere with the interpretation of any colour change. For non-interfering compounds, a blue colour in the well was interpreted as the absence of growth, and a pink colour was scored as growth.

The evaluation of the in vitro antimycobacterial activity of the compounds was additionally performed against *M. smegmatis* ATCC 700084, *M. marinum* CAMP 5644 and *M. kansasii* DSM 44162. The broth dilution micro-method in Middlebrook 7H9 medium (Difco, Lawrence, KS, USA) supplemented with ADC Enrichment (Becton, Dickinson and Comp.) was used to determine MIC as previously described [37]. The compounds were dissolved in DMSO (Sigma-Aldrich), and the final concentration of DMSO did not exceed 2.5% of the total solution composition. Final concentrations of the evaluated compounds ranging from 256 μg/mL to 0.125 μg/mL were obtained by twofold serial dilution of the stock solution in a microtiter plate with sterile medium. Bacterial inocula were prepared by transferring colonies from culture to sterile water. The cell density was adjusted to 0.5 McFarland units using a densitometer (Densi-La-Meter, LIAP, Riga, Latvia). The final inoculum was made by 1:1000 dilution of the suspension with sterile water. Drug-free controls, sterility controls and controls consisted of medium and DMSO (Sigma-Aldrich, St. Louis, MO, USA) alone were included. The determination of results was performed visually after 3 days of static incubation in darkness at 37 °C in an aerobic atmosphere for *M. smegmatis*, after 7 days of static incubation in darkness at 37 °C in an aerobic atmosphere for *M. kansasii* and after 21 days of static incubation in darkness at 28 °C in an aerobic atmosphere for *M. marinum*. Ciprofloxacin (CIP), rifampicin (RIF) and isoniazid (INH) (Sigma-Aldrich, St. Louis, MO, USA) were used as the standards.

#### 3.2.2. In Vitro Antifungal Susceptibility Testing

Microdilution method was used for testing the antifungal activities of newly synthesized compounds against *Candida albicans* CCM 8261 and ATCC 90028, *C. krusei* CCM 8271, *C. parapsilosis* CCM 8260 (Czech Collection of Microorganisms, Brno, Czech Republic) [38]. Tested compounds were diluted in RPMI-1640 (Sigma) broth to concentrations 128–0.016 µg/mL. Flucytosine (FLU) (Sigma) and amphotericin B (Amph) were used as positive controls. The plates were inoculated by an inoculum prepared in RPMI-1640 broth. The final concentration of fungal cells was 5 × 10^2^–2.5 × 10^3^ CFU/mL in each well. The plates were incubated at 37 °C for 24 (*C. albicans*, *C. krusei*) or 48 (*C. parapsilosis*) hours. Drug-free controls were included. MIC was defined as 80% or greater (IC_80_) reduction of growth in comparison with the control [35].

MIC determination for *Aspergillus brasiliensis* was performed in RPMI 1640 broth with glutamine supplemented with 2% glucose, following the same scheme as for bacteria, procedure b). After incubation period (48 h, 35 °C, aerobically in dark), XTT (2*H*-tetrazolium, 2,3-bis(2-methoxy-4-nitro-5-sulfophenyl)-5-[(phenylamino)carbonyl]-hydroxide) (10 mg/mL) in combination with menadione (1 mg/mL in acetone) (7:1, *v*/*v*) was added [36]. Absorbance was read at 540 nm. Amph was used as a positive control and solvent and media (no microorganisms added) as negative controls.

#### 3.2.3. Minimum Biofilm Eradication Assay

Biofilm eradication screening was performed on the following microorganisms: *S. aureus* ATCC 6538, *S. pneumoniae* MFBF 10373, *E. faecalis* ATCC 29212, *B. cereus* ATCC 11778, *B. subtilis* ATCC 6633, *E. coli* ATCC 10536, *P. aeruginosa* ATCC 27853, *S. marcescens* ATCC 10905, *P. mirabilis* MFBF 10430, *S. enteritidis* MFBF 11945, *A. baumannii* MFBF 10913, and *C. albicans* ATCC 90028. MBECs of fumardiamides **4a**–**f** and succindiamides **5a**–**f** were determined as follows [39]. Each well (96-well plate) was filled with 100 µl of bacterial (10^7^ CFU/mL) or yeast (5 × 10^6^ CFU/mL) suspension. When the inhibition of yeast biofilm formation was tested, the wells were pre-treated with fetal bovine serum (FBS) (250 µl per well). Negative controls contained broth only. Positive controls were performed using standard antimicrobial drugs gentamycin (Gen) and Amph, respectively. The plates were covered and incubated aerobically for 24 h (bacteria) or 48 h (yeast) at 37 °C. Following incubation period, each well was aspirated, washed three times and vigorously shaken to remove all non-adherent bacteria/yeast. The remaining attached cells were fixed with methanol (15 min) and the plates were left to dry overnight. Formed biofilm was stained with crystal violet (1%, 5 min). Excess stain was rinsed by placing the plate under running tap water and the plates were left to dry. Adherent cells were solubilized using ethanol. The absorbance was read at 570 nm. The MBEC value represents the lowest dilution of a compound at which bacteria fail to grow.

#### 3.2.4. Antiviral Evaluation

Antiviral activity of compounds **4a**–**f** and **5a**–**f** was determined as described previously [40]. Cytotoxicity and antiviral activity assay towards herpes simplex virus (HSV) strains HSV-1 KOS, HSV-2 G, HSV-1 TK-KOS ACVr, vaccinia virus, adenovirus-2, human coronavirus (229E) in HEL cell cultures, vesicular stomatitis virus, coxsackie virus B4, respiratory syncytial virus in Hela cell cultures, para-influenza-3 virus, reovirus-1, sindbis, coxsackie virus B4, Punta Toro virus, yellow fever virus in Vero cell cultures, while influenza A/H1N1 A/Ned/378/05, influenza A/H3N2 A/HK/7/87 and influenza B B/Ned/537/05 viruses in Madin-Darby Canine Kidney (MDCK) cell cultures were performed. On the day of the infection, growth medium was aspirated and replaced by serial dilutions of the test compounds. Virus was then added to each well, diluted to obtain a viral input of 100 CCID_50_ (CCID_50_ being the virus dose that is able to infect 50% of the cell cultures). Mock-treated cell cultures receiving solely the test compounds were included, to determine their cellular cytotoxicity. After 3 to 10 days of incubation the virus-induced cytopathicity was determined by visual scoring of the cytopathic effect (CPE) (light microscopic evaluation of the virus-induced CPE and inhibition of evaluated compounds), as well as by measuring the cell viability with the colorimetric formazan-based MTS assay (3-(4,5-dimethylthiazol-2-yl)-5-(3-carboxymethoxyphenyl)-2-(4-sulfophenyl)-2*H*-tetrazolium). All experiments were performed in duplicate. Antiviral activity was expressed as EC_50_. The activities were compared with the activities of the parent drug PQ, DS-10.000 (dextran sulfate, approx. MW = 10.000) and standard antiviral drugs: ribavirin (Rib), mycophenolic acid (MPA), brivudin, cidofovir, acyclovir, gancyclovir, zalcitabine, alovudine, UDA, zanamivir, amantadine and rimantadine.

#### 3.2.5. Cytostatic Activity

Cytostatic activity was evaluated in vitro on nine different types of human tumor cell lines: Capan-1, Hap1, HCT-116, and NCI-H460, as well as hematological tumors such as DND-41, HL-60, K-562, MM.1S and Z-138 as described previously [41]. All human tumor cell lines were acquired from the American Type Culture Collection (ATCC, Manassas, VA, USA), except for the DND-41 cell line which was purchased from the Deutsche Sammlung von Mikroorganismen und Zellkulturen (DSMZ Leibniz-Institut, Braunschweig, Germany) and the Hap1 cell line which was purchased from Horizon Discovery (Waterbeach, UK). Capan-1, Hap1, HL-60, K-562, Z-138, MM.1S and DND-41 were cultured in Iscove′s Modified Dulbecco′s Medium (IMDM, Gibco Life Technologies, Gaithersburg, MD, USA), HCT-116 were grown in McCoy′s 5A Medium (Gibco Life Technologies) and NCI-H460 were cultured in RPMI (Gibco Life Technologies). All media were supplemented with 10% FBS (HyClone, GE Healthcare Life Sciences, USA). Adherent cell lines HCT-116, Hap1, NCI-H460, and Capan-1 cells were seeded at a density between 400 and 1250 cells per well, in 384-well, black-walled, clear-bottomed tissue culture plates (Greiner Bio-One, Kremsmünster, Germany). After overnight incubation, cells were treated with test compounds at four different concentrations ranging from 100 to 0.8 µM. Suspension cell lines HL-60, K-562, Z-138, MM.1S, and DND-41 were seeded at densities ranging from 3000 to 10,000 cells per well in 384-well, black-walled, clear-bottomed tissue culture plates containing test compounds at the same four concentrations. The plates were incubated and monitored at 37 °C for 72 h in an IncuCyte (Essen BioScience Inc., Ann Arbor, MI, USA) for real-time imaging. Images were taken every 3 h, with one field image per well under 10× magnification. All compounds were tested with duplicate data points and averaged. The activities were compared with the activities of the parent drug PQ and standard anticancer drugs docetaxel (DXT), etoposide (EPEG) and staurosporine (STS).

#### 3.2.6. Interaction with Glutathione (GSH)

Fumardiamide **4b** (1.25 μM) was incubated with GSH (125 μM) in ammonium formate buffer (pH = 7.4) containing 10% acetonitrile at 37 °C for 216 h [33]. The progress of the reactions was monitored with the percent of remaining fumardiamide determined by MS using an internal standard (*N*-(benzyloxy)-*N*′-{4-[(6-methoxyquinolin-8-yl)amino]pentyl}butanediamide). Aliquots of the reaction mixture (taken after 0, 4.5, 26, 52, 124 and 216 h) were analysed with Synapt G2-Si ESI-QTOF-MS system (Waters, Milford, USA). The aliquots were diluted 10 times with acetonitrile and sprayed at a flow rate of 50 μL/min using the fluidics system of the instrument. MS conditions were set as follows: positive ion mode, capillary voltage 3 kV, sampling cone voltage 10 V, source temperature 120 °C, desolvation temperature 350 °C, desolvation gas flow 800 L/h. Mass spectra were recorded from 100–1000 *m/z* at a frequency of 1 Hz. Data were acquired and analysed with Waters MassLynx v4.1 software.

## 4. Conclusions

Twelve novel PQ-derivatives of diamide type were designed and synthesized. These compounds differ in the type of spacer and/or halogen atom in aniline region. Compounds **4a**–**f** are fumardiamides and **5a**–**f** succindiamides. Compounds **4a**,**b**, **5a**,**b** are fluoro, **4c**,**d**, **5c**,**d** chloro and **4e**,**f**, **5e**,**f** are trifluoroderivatives. All new compounds were screened for antibacterial, antitubercular, antiviral and cytostatic activity as well as biofilm eradication ability. In all biological assays, fumardiamides **4** were superior to succindiamides **5**, which indicates that the double bond conjugated to the carbonyl was important for the activity. With their high bioactivity, low cytotoxicity and convenient drug-like properties, *p*-substituted derivatives **4b**,**d**,**f** provide a strong basis for further research and optimization of novel agents useful in the treatment of bacterial and biofilm-associated infections, while *m*-substituted derivatives **4a**,**c**,**e** could be potential leads for the development of antitumor agents.

## Figures and Tables

**Table 1:** Antimicrobial susceptibility assay in vitro (minimum inhibitory concentration (MIC) determination) for compounds **4a**–**f**.

Compd.	MIC (µg/mL)								
	*Staphylococcus aureus* ATCC 6538	*Streptococcus pneumoniae* MFBF 10373	*Enterococcus faecalis* ATCC 29212	*Bacillus cereus* ATCC 11778	*Bacillus subtilis* ATCC 6633	*Acinetobacter baumannii* MFBF 10913	*Mycobacterium tuberculosis* H37Ra	*Mycobacterium marinum* CAMP 5644	*Candida albicans* ATCC 90028
**4a**	>100	>100	128	>100	50	>100	128	>256	>100
**4b**	12.5	12.5	64	25	25	25	32	64	>100
**4c**	12.5	25	256	25	50	25	128	128	>100
**4d**	6.1	12.5	64	>100	>100	12.5	128	64	12.5
**4e**	50	50	128	>100	>100	25	128	256	>100
**4f**	50	50	64	>100	>100	25	64	64	>100
PQ ^1^	50	50	128	70	80	50	256	256	>100
TC ^2^	0.3	0.3	–	0.3	0.3	3	–	–	–
CIP ^3^	–	–	–	–	–	–	16.0	0.3	–
INH ^4^	–	–	–	–	–	–	5.0	64.0	–
RIF ^5^	–	–	–	–	–	–	8.7	2.2	–
Amph ^6^	–	–	–	–	–	–	–	–	0.5

^1^ PQ—primaquine; ^2^ TC—tetracycline; ^3^ CIP—ciprofloxacin; ^4^ INH—isoniazid; ^5^ RIF—rifampicin; ^6^ Amph—amphotericin B.

**Table 2:** Sensitivity of microbial strains to fumardiamides **4a**–**f** expressed as minimum biofilm eradication concentrations (MBEC).

Compd.	MBEC (µg/mL)										
	*Staphylococcus aureus* ATCC 6538	*Streptococcus pneumonia* MFBF 10373	*Enterococcus faecalis* ATCC 29212	*Bacillus cereus* ATCC 11778	*Bacillus subtilis* ATCC 6633	*Escherichia coli* ATCC 10536	*Pseudomonas aeruginosa* ATCC 27853	*Serratia marcescens* ATCC 10905	*Proteus mirabilis* MFBF 10430	*Salmonella enteritidis* MFBF 11945	*Acinetobacter baumannii* MFBF 10913
**4a**	12.5	12.5	25	>100	50	100	25	25	>100	>100	6.3
**4b**	12.5	25	25	25	25	50	12.5	>100	25	>100	25
**4c**	12.5	100	25	25	50	50	50	>100	>100	>100	100
**4d**	25	50	50	>100	>100	50	>100	>100	>100	>100	100
**4e**	50	50	50	>100	>100	50	50	>100	>100	>100	100
**4f**	25	50	50	>100	>100	50	25	>100	>100	>100	100
PQ ^1^	50	70	>100	70	80	65	65	>100	>100	70	50
Gen ^2^	12.5	25	50	50	50	25	50	50	50	50	25

^1^ PQ—primaquine; ^2^ Gen—gentamycin.

**Table 3:** Cytotoxicity and antiviral activity of fumardiamides **4a**–**f** evaluated in Vero cell cultures.

Compd.	Cytotoxicity		EC_50_^1^ (µM)											
	CC_50_^2^	MCC ^3^	Para-Influenza-3 Virus		Reovirus-1		Sindbis Virus		Coxsackie Virus B4		Punta Toro Virus		Yellow Fever Virus	
			MTS	Visual CPE Score	MTS	Visual CPE Score	MTS	Visual CPE Score	MTS	Visual CPE Score	MTS	Visual CPE Score	MTS	Visual CPE Score
**4a**	46.9	–	>50	–	>50	–	>50	–	>50	–	>50	–	>50	–
**4b**	>50	–	>50	–	>50	–	>50	–	>50	–	>50	–	>50	–
**4c**	>50	10	>50	>50	3.8	>50	3.0	>50	>50	>50	5.5	>50	>50	>50
**4d**	>50	–	>50	–	>50	–	>50	–	>50	–	>50	–	>50	–
**4e**	>50	10	>50	>50	3.1	>50	5.3	4.2	3.1	2.7	5.4	>50	>50	>50
**4f**	>50	–	>50	–	>50	–	>50	–	>50	–	>50	–	>50	–
PQ	>50	–	>50	–	>50	–	>50	–	>50	–	>50	–	>50	–
DS-10.000 ^4^	>100	>100	>100	>100	>100	>100	4.0	10	>100	34	16	8.9	1.6	>100
RIB ^5^	>250	>250	73	111	107	126	>250	11	>250	>250	85	111	119	>250
MPA ^6^	>100	>100	1.0	0.8	0.6	0.8	12	1.7	>100	>100	11	8.9	0.5	0.8

^1^ Concentration required to reduce virus-induced cytopathicity by 50%, as determined by visual scoring of the cytopathic effect (CPE), or by measuring the cell viability with the colorimetric formazan-based 3-(4,5-dimethylthiazol-2-yl)-5-(3-carboxymethoxyphenyl)-2-(4-sulfophenyl)-2*H*-tetrazolium (MTS) assay; ^2^ Concentration that causes 50% cytotoxic effect, as determined by measuring the cell viability with the colorimetric formazan-based MTS assay; ^3^ Minimum cytotoxic concentration, i.e., the minimum concentration that causes a microscopically detectable alteration of normal cell morphology; ^4^ Concentration in µg/mL; ^5^ RIB—ribavirin; ^6^ MPA—mycophenolic acid. All experiments were performed in duplicate.

**Table 4:** Antiproliferative screening of fumardiamides **4a**–**f** in diverse human tumor cell lines.

Compd.	IC_50_ (µM)								
	Capan-1	Hap1	HCT-116	NCI-H460	DND-41	HL-60	K-562	MM.1S	Z-138
**4a**	16.9	19.3	22.3	15.5	6.7	5.7	31.2	13.0	8.4
**4b**	50.5	66.4	>100	29.1	>100	>100	70.5	>100	>100
**4c**	69.9	>100	73.0	43.7	8.4	>100	>100	>100	46.5
**4d**	91.8	>100	>100	>100	>100	>100	>100	>100	>100
**4e**	56.5	>100	>100	34.2	8.9	>100	71.2	>100	68.4
**4f**	79.4	>100	>100	>100	>100	>100	>100	>100	>100
PQ ^1^	18.7	42.7	30.9	52.6	11.4	2.2	35.2	28.3	7.1
DXT ^2^	0.75	1.17	7.66	1.30	0.94	1.24	1.22	3.38	5.42
EPEG ^3^	0.15	0.04	1.35	0.09	0.03	0.03	0.01	0.97	0.02
STS ^4^	0.66	3.56	0.78	1.66	6.96	13.10	0.23	1.61	0.40

^1^ PQ—primaquine; ^2^ DXT—docetaxel (nM); ^3^ EPEG—etoposide; ^4^ STS—staunoporin (nM). All compounds were tested with duplicate data points and averaged.

## Supplementary Materials

The following are available online: Table S1. Properties of novel compounds calculated with Chemicalize.org program. The Lipinski and Gelovani parameters; Table S2. Analytical and spectral data of compounds **4a**–**f** and **5a**–**f**; Table S3. ^1^H and ^13^C NMR spectra of amides **4a**–**f** and **5a**–**f**; Table S4. Interaction of fumardiamide **4b** with GSH; Figure S1. Interaction of fumardiamide **4b** with GSH (♦). Control: (*N*-(benzyloxy)-*N*′-{4-[(6-methoxyquinolin-8-yl)amino]pentyl}butanediamide) (∙). Spectra of all compounds.

## References

[1] Clayden J., Greeves N., Warren S. (2012). Organic Chemistry.

[2] Smaill J.B., Rewcastle G.W., Loo J.A., Greis K.D., Chan O.H., Reyner E.L., Lipka E., Showalter H.D., Vincent P.W., Elliott W.L. (2000). Tyrosine kinase inhibitors. 17. Irreversible inhibitors of the epidermal growth factor receptor: 4-(phenylamino)quinazoline- and 4-(phenylamino)pyrido[3,2-*d*]pyrimidine-6-acrylamides bearing additional solubilizing functions. J. Med. Chem..

[3] Minami Y., Shimamura T., Shah K., LaFramboise T., Glatt K.A., Liniker E., Borgman C.L., Haringsma H.J., Feng W., Weir B.A. (2007). The major lung cancer-derived mutants of ERBB2 are oncogenic and are associated with sensitivity to the irreversible EGFR/ERBB2 inhibitor HKI-272. Oncogene.

[4] Baselga J., Coleman R.E., Cortés J., Janni W. (2017). Advances in the management of HER2-positive early breast cancer. Crit. Rev. Oncol. Hematol..

[5] Jackson P.A., Widen J.C., Harki D.A., Brummond K.M. (2017). Covalent modifiers: A chemical perspective on the reactivity of α,β-unsaturated carbonyls with thiols via hetero-Michael addition reactions. J. Med. Chem..

[6] Zhang X., Li X., You Q., Zhang X. (2017). Prodrug strategy for cancer cell-specific targeting: A recent overview. Eur. J. Med. Chem..

[7] Compound Summary for CID 5281081. https://pubchem.ncbi.nlm.nih.gov/compound/entacapone#section=Top.

[8] Matthews D.A., Dragovich P.S., Webber S.E., Fuhrman S.A., Patick A.K., Zalman L.S., Hendrickson T.F., Love R.A., Prins T.J., Marakovits J.T. (1999). Structure-assisted design of mechanism-based irreversible inhibitors of human rhinovirus 3C protease with potent antiviral activity against multiple rhinovirus serotypes. Proc. Natl. Acad. Sci. USA.

[9] Buzdar A.U., Robertson J.F., Eiermann W., Nabholtz J.M. (2002). An overview of the pharmacology and pharmacokinetics of the newer generation aromatase inhibitors anastrozole, letrozole, and exemestane. Cancer.

[10] Somberg J.C., Molnar J. (2009). The pleiotropic effects of ethacrynic acid. Am. J. Ther..

[11] Dawson R.M. (1998). The toxicology of microcystins. Toxicon.

[12] Eusugi S., Fujisawa N., Yoshida J., Watanabe M., Dan S., Yamori T., Shiono Y., Kimura K., Pyrrocidine A. (2016). A metabolite of endophytic fungi, has a potent apoptosis-inducing activity against HL60 cells through caspase activation via the Michael addition. J. Antibiot..

[13] Ramsay J.R., Suhrbier A., Aylward J.H., Ogbourne S., Cozzi S.J., Poulsen M.G., Baumann K.C., Welburn P., Redlich G.L., Parsons P.G. (2011). The sap from *Euphorbia peplus* is effective against human nonmelanoma skin cancers. Br. J. Dermatol..

[14] Lebwohl M., Swanson N., Anderson L.L., Melgaard A., Xu Z., Berman B. (2012). Ingenol mebutate gel for actinic keratosis. N. Engl. J. Med..

[15] Picato® gel–FDA. https://www.accessdata.fda.gov/drugsatfda_docs/label/2012/202833lbl.pdf..

[16] Sebök B., Bonnekoh B., Geisel J., Mahrle G. (1994). Antiproliferative and cytotoxic profiles of antipsoriatic fumaric acid derivatives in keratinocyte cultures. Eur. J. Pharmacol..

[17] Smith D. (2017). Fumaric acid esters for psoriasis: A systematic review. Ir. J. Med. Sci..

[18] Kocaadam B., Şanlier N. (2017). Curcumin, an active component of turmeric (*Curcuma longa*), and its effects on health. Crit. Ver. Food Sci. Nutr..

[19] Seca A.M.L., Pinto D.C.G.A. (2018). Plant secondary metabolites as anticancer agents: Successes in clinical trials and therapeutic application. Int. J. Mol. Sci..

[20] Pavić K., Perković I., Cindrić M., Pranjić M., Martin-Kleiner I., Kralj M., Schols D., Hadjipavlou-Litina D., Katsori A.-M., Zorc B. (2014). Novel semicarbazides and ureas of primaquine with bulky aryl or hydroxyalkyl substituents: Synthesis, cytostatic and antioxidative activity. Eur. J. Med. Chem..

[21] Perković I., Antunović M., Marijanović I., Pavić K., Ester K., Kralj M., Vlainić J., Kosalec I., Schols D., Hadjipavlou-Litina D. (2016). Novel urea and bis-urea primaquine derivatives with hydroxyphenyl and halogenphenyl substituents: Synthesis and biological evaluation. Eur. J. Med. Chem..

[22] Guzman J.D. (2014). Natural cinnamic acids, synthetic derivatives and hybrids with antimicrobial activity. Molecules.

[23] Kakwani M.D., Suryavanshi P., Ray M., Rajan M.G.R., Majee S., Samad A., Devarajan P., Degani M.S. (2011). Design, synthesis and antimycobacterial activity of cinnamide derivatives: A molecular hybridization approach. Bioorg. Med. Chem. Lett..

[24] De P., Baltas M., Bedos-Belval F. (2011). Cinnamic acid derivatives as anticancer agents-a review. Curr. Med. Chem..

[25] Pavić K., Perković I., Gilja P., Kozlina F., Ester K., Kralj M., Schols D., Hadjipavlou-Litina D., Pontiki E., Zorc B. (2016). Design, synthesis and biological evaluation of novel primaquine-cinnamic acid conjugates of amide and acylsemicarbazide type. Molecules.

[26] Pavić K., Perković I., Pospíšilová Š., Machado M., Fontinha D., Prudêncio M., Jampilek J., Coffey A., Endersen L., Rimac H. (2018). Primaquine hybrids as promising antimycobacterial and antimalarial agents. Eur. J. Med. Chem..

[27] Vlainić J., Kosalec I., Pavić K., Hadjipavlou-Litina D., Pontiki E., Zorc B. (2018). Insights into biological activity of ureidoamides with primaquine and amino acid moieties. J. Enzyme Inhib. Med. Chem..

[28] Levatić J., Pavić K., Perković I., Uzelac L., Ester K., Kralj M., Kaiser M., Rottmann M., Supek F., Zorc B. (2018). Machine learning prioritizes synthesis of primaquine ureidoamides with high antimalarial activity and attenuated cytotoxicity. Eur. J. Med. Chem..

[29] Beus M., Rajić Z., Maysinger D., Mlinarić Z., Antunović M., Marijanović I., Fontinha D., Prudêncio M., Held J. SAHA-primaquine hybrids (sahaquines) as potential anticancer and antimalarial compounds. Chem. Open.

[30] Chemicalize, 2017, ChemAxon Ltd.. http://www.chemicalize.org.

[31] Hufnagel D.A., Price J.E., Stephenson R.E., Kelley J., Benoit M.F., Chapman M.R. (2018). Thiol starvation induces redox-mediated dysregulation of *Escherichia coli* biofilm components. J. Bacteriol..

[32] Lee S.F., Davey L. (2017). Disulfide bonds: A key modification in bacterial extracytoplasmic proteins. J. Dent. Res..

[33] Flanagan M.E., Abramite J.A., Anderson D.P., Aulabaugh A., Dahal U.P., Gilbert A.M., Li C., Montgomery J., Oppenheimer S.R., Ryder T. (2014). Chemical and computational methods for the characterization of covalent reactive groups for the prospective design of irreversible inhibitors. J. Med. Chem..

[34] Oravcova V., Zurek L., Townsend A., Clark A.B., Ellis J.C., Cizek A. (2014). American crows as carriers of vancomycin-resistant enterococci with vanA gene. Environ. Microbiol..

[35] Clinical and Laboratory Standards Institute (2012). Performance Standards for Antimicrobial Susceptibility Testing.

[36] EUCAST, European Committee on Antimicrobial Susceptibility Testing (2013). Determination of minimum inhibitory concentrations (MICs) of antibacterial agents by broth micro dilution. EUCAST Discussion Document. Clin. Microbiol. Infect..

[37] Schwalbe R., Steele-Moore L., Goodwin A.C. (2007). Antimicrobial Susceptibility Testing Protocols.

[38] Sheehan D.J., Espinel-Ingroff A., Steele M., Webb C.D. (1993). Antifungal susceptibility testing of yeasts: A brief overview. Clin. Infect. Dis..

[39] Stepanović S., Vuković D., Hola V., Di Bonaventura G., Djukić S., Cirković I., Ruzicka F. (2007). Quantification of biofilm in microtiter plates: Overview of testing conditions and practical recommendations for assessment of biofilm production by *Staphylococci*. APMIS.

[40] Tzioumaki N., Manta S., Tsoukala E., Vande Voorde J., Liekens S., Komiotis D., Balzarini J. (2011). Synthesis and biological evaluation of unsaturated keto and exomethylene D-arabinopyranonucleoside analogs: Novel 5-fluorouracil analogs that target thymidylate synthase. Eur. J. Med. Chem..

[41] Li Q., Lescrinier E., Groaz E., Persoons L., Daelemans D., Herdewijn P., De Jonghe S. (2018). Synthesis and biological evaluation of pyrrolo[2,1-f][1,2,4]triazine C-nucleosides with a ribose, 2′-deoxyribose, and 2′,3′-dideoxyribose sugar moiety. ChemMedChem..

