# Supplementary material for: Asymmetric Primaquine and Halogenaniline Fumardiamides as Novel Biologically Active Michael Acceptors
Source: Molecules. 2018 Jul 14;23(7):1724. doi: 10.3390/molecules23071724 (PMC6100582; doi:10.3390/molecules23071724)
Supplement: Supplementary file 1 [file molecules-23-01724-s001.pdf]

**Supporting Material**

**Table S1.** Properties of novel compounds calculated with Chemicalize.org program[1]. The Lipinski's and Gelovani's parameters.

| Compd. | Molecular formula | Number of atoms | MW | log *P* | H-bond donor | H-bond acceptor | Lipinski score[2] | MR[3] (cm3/mol) | TPSA[4] (Å$^2$) |
|---|---|---|---|---|---|---|---|---|---|
| **4a** | $C_{25}H_{27}FN_4O_3$ | 60 | 450.514 | 3.22 | 3 | 5 | 4 | 128.42 | 92.35 |
| **4b** | $C_{25}H_{27}FN_4O_3$ | 60 | 450.514 | 3.22 | 3 | 5 | 4 | 128.42 | 92.35 |
| **4c** | $C_{25}H_{27}ClN_4O_3$ | 60 | 466.970 | 3.68 | 3 | 5 | 4 | 133.00 | 92.35 |
| **4d** | $C_{25}H_{27}ClN_4O_3$ | 60 | 466.970 | 3.68 | 3 | 5 | 4 | 133.00 | 92.35 |
| **4e** | $C_{26}H_{27}F_3N_4O_3$ | 63 | 500.522 | 3.96 | 3 | 5 | 4 | 134.17 | 92.35 |
| **4f** | $C_{26}H_{27}F_3N_4O_3$ | 63 | 500.522 | 3.96 | 3 | 5 | 4 | 134.17 | 92.35 |
| **5a** | $C_{25}H_{29}FN_4O_3$ | 62 | 452.530 | 2.86 | 3 | 5 | 4 | 127.35 | 92.35 |
| **5b** | $C_{25}H_{29}FN_4O_3$ | 62 | 452.530 | 2.86 | 3 | 5 | 4 | 127.35 | 92.35 |
| **5c** | $C_{25}H_{29}ClN_4O_3$ | 62 | 468.980 | 3.32 | 3 | 5 | 4 | 131.93 | 92.35 |
| **5d** | $C_{25}H_{29}ClN_4O_3$ | 62 | 468.980 | 3.32 | 3 | 5 | 4 | 131.93 | 92.35 |
| **5e** | $C_{26}H_{29}F_3N_4O_3$ | 65 | 502.538 | 3.60 | 3 | 5 | 4 | 133.10 | 92.35 |
| **5f** | $C_{26}H_{29}F_3N_4O_3$ | 65 | 502.538 | 3.60 | 3 | 5 | 4 | 133.10 | 92.35 |

[1] Chemicalize, 2017, ChemAxon Ltd. Available from: http://www.chemicalize.org; [2] Out of four; [3] MR – molar refractivity; [4] TPSA – topological polar surface area.

**Table S2.** Analytical and spectral data of compounds **4a-f** and **5a-f.**

| Compd. | Yield (%) | m.p. (°C) | IR (ATR): $\nu_{max}$ (cm$^{-1}$) | MS (*m*/*z*) | Molecular formula (*M*$_r$) | CHN analysis calcd./found (%) | | |
|---|---|---|---|---|---|---|---|---|
| **4a** | 34 | 203–204 | 3388, 3319, 3269, 3080, 2961, 2935, 2866, 1630, 1554, 1520, 1452, 1387, 1334, 1201, 1158, 782, 680 | 451.1 (M+1) | $C_{25}H_{27}FN_4O_3$ (450.514) | 66.65<br>66.32 | 6.04<br>6.30 | 12.44<br>12.49 |
| **4b** | 58 | 226–227 | 3386, 3294, 3072, 2963, 2928, 2863, 1635, 1548, 1513, 1452, 1391, 1330, 1212, 1160, 1051, 973, 829, 673 | 451.1 (M+1) | $C_{25}H_{27}FN_4O_3$ (450.514) | 66.65<br>66.47 | 6.04<br>6.38 | 12.44<br>12.35 |
| **4c** | 42 | 187–188 | 3381, 3298, 3068, 2959, 2928, 2863, 1635, 1591, 1521, 1465, 1419, 1386, 1331, 1210, 1163, 976, 821, 783, 670 | 467.0 (M+1) | $C_{25}H_{27}ClN_4O_3$ (466.970) | 64.30<br>64.21 | 5.83<br>6.05 | 12.00<br>11.78 |

| | | | | | | | | |
|---|---|---|---|---|---|---|---|---|
| **4d** | 49 | 223–226 | 3381, 3289, 3071, 2959, 2931, 2864, 1640, 1526, 1452, 1388, 1331, 1210, 1163, 1094, 1049, 973, 822, 787, 686, 631, 507 | 467.0 (M+1) | $C_{25}H_{27}ClN_4O_3$ (466.970) | 64.30<br>64.21 | 5.83<br>5.56 | 12.00<br>11.83 |
| **4e** | 31 | 149–150 | 3399, 3357, 3282, 3094, 2960, 2935, 2867, 1651, 1621, 1563, 1526, 1452, 1388, 1331, 1168, 1122, 973, 893, 788, 694 | 501.1 (M+1) | $C_{26}H_{27}F_3N_4O_3$ (500.522) | 62.39<br>62.25 | 5.44<br>5.76 | 11.19<br>11.08 |
| **4f** | 34 | 189–191 | 3387, 3309, 3071, 2963, 2932, 1636, 1527, 1457, 1417, 1390, 1328, 1213, 1166, 1122, 1065, 970, 832, 681 | 501.1 (M+1) | $C_{26}H_{27}F_3N_4O_3$ (500.522) | 62.39<br>62.17 | 5.44<br>5.61 | 11.19<br>11.40 |
| **5a** | 63 | 140–142 | 3391, 3283, 3145, 3080, 2959, 2926, 1744, 1646, 1615, 1555, 1507, 1387, 1224, 1202, 1167, 1157, 838, 815, 786, 681 | 453.3 (M+1) | $C_{25}H_{29}FN_4O_3$ (452.530) | 66.35<br>66.25 | 6.46<br>6.50 | 12.38<br>12.20 |
| **5b** | 80 | 118–120 | 3391, 3283, 3145, 3080, 2959, 2926, 1744, 1646, 1615, 1555, 1507, 1387, 1224, 1202, 1167, 1157, 838, 815, 786, 681 | 453.4 (M+1) | $C_{25}H_{29}FN_4O_3$ (452.530) | 66.35<br>66.53 | 6.46<br>6.29 | 12.38<br>12.55 |
| **5c** | 49 | 156–158 | 3390, 3287, 3242, 3180, 3102, 3074, 2962, 2922, 2856, 1738, 1650, 1612, 1591, 1572, 1539, 1516, 1422, 1388, 1202, 1066, 816, 787, 698, 682 | 469.2 (M+1) | $C_{25}H_{29}ClN_4O_3$ (468.980) | 64.03<br>63.91 | 6.23<br>6.33 | 11.95<br>11.70 |
| **5d** | 60 | 152–153 | 3390, 3287, 3242, 3180, 3102, 3074, 2962, 2922, 2856, 1738, 1650, 1612, 1591, 1572, 1539, 1516, 1422, 1388, 1202, 1066, 816, 787, 698, 682 | 469.3 (M+1) | $C_{25}H_{29}ClN_4O_3$ (468.980) | 64.03<br>64.35 | 6.23<br>6.57 | 11.95<br>12.13 |
| **5e** | 90 | 146–149 | 3394, 3302, 3263, 3216, 3164, 3097, 2963, 2928, 2859, 1651, 1617, 1562, 1518, 1450, 1389, 1331, 1265, 1173, 1127, 1064, 894, 816 | 503.3 (M+1) | $C_{26}H_{29}F_3N_4O_3$ (502.538) | 62.14<br>62.25 | 5.82<br>5.99 | 11.15<br>11.08 |
| **5f** | 50 | 163–165 | 3387, 3287, 3256, 3198, 3123, 3071, 2963, 2925, 2859, 1652, 1614, 1547, 1515, 1452, 1419, 1389, 1327, 1264, 1169, 1124, 1064, 846, 784 | 503.3 (M+1) | $C_{26}H_{29}F_3N_4O_3$ (502.538) | 62.14<br>62.39 | 5.82<br>5.76 | 11.15<br>11.41 |

**Table S3.** $^{1}H$ and $^{13}C$ NMR spectra of amides **4a-f** and **5a-f**

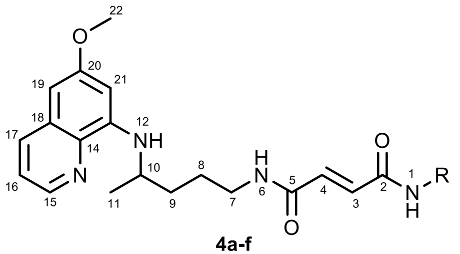

**4a-f**

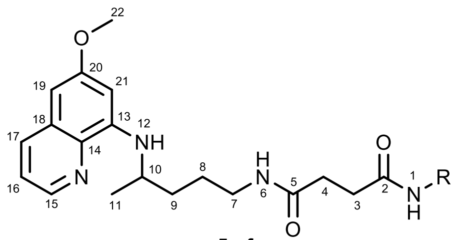

**5a-f**

| **Compd.** | **R** | **$^{1}H$ NMR (DMSO-$d_6$, $\delta$ ppm, $J$/Hz)** | **$^{13}C$ NMR (DMSO-$d_6$, $\delta$ ppm, $J$/Hz)** |
|---|---|---|---|
| **4a** | 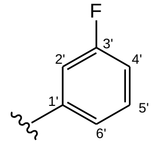 | 10.63 (s, 1H, 1), 8.55-8.53 (dd, 1H, 15, $J$ = 1.6, 4.2), 8.52 (t, 1H, 6, $J$ = 5.4), 8.09-8.06 (dd, 1H, 17, $J$ = 1.5, 8.3), 7.69 (d, 2H, 6', $J$ = 11.7), 7.45-7.41 (m, 1H, 16), 7.39-7.36 (m, 2H, 2', 4'), 7.06-7.96 (m, 2H, 3, 4), 6.96-6.92 (m, 1H, 5′), 6.47 (d, 1H, 21, $J$ = 2.4), 6.28 (d, 1H, 19, $J$ = 2.4), 6.15 (d, 1H, 12, $J$ = 8.8), 3.82 (s, 3H, 22), 3.70-3.61 (m, 1H, 10), 3.24-3.18 (m, 2H, 7), 1.74-1.64, 1.63-1.52 (2m, 4H, 8, 9), 1.22 (d, 3H, 11, $J$ = 6.3) | 163.25 (5), 162.62 (2), 162.09 (3', $J$ = 242.0), 159.00 (20), 144.63 (13), 144.23 (15), 140.50 (1', d, $J$ = 11.5), 134.79 (17), 134.52 (14), 134.40 (3), 132.28 (4), 130.50 (5', d, $J$ = 9.5), 129.57 (18), 122.09 (16), 115.14 (6'), 110.28 (2', d, $J$ = 18.8), 106.13 (4', d, $J$ = 27.2), 96.14 (21), 91.62 (19), 54.96 (22), 46.97 (10), 38.95 (7), 33.45 (9), 25.77 (8), 20.21 (11) |
| **4b** | 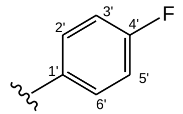 | 10.49 (s, 1H, 1), 8.55-8.53 (dd, 1H, 15, $J$ = 1.6, 4.2), 8.52 (t, 1H, 6, $J$ = 5.4), 8.09-8.06 (dd, 1H, 17, $J$ = 1.5, 8.3), 7.72-7.68 (dd, 2H, 2', 6', $J$ = 5.0, $J$ = 8.9), 7.45-7.41 (m, 1H, 16), 7.18 (t, 2H, 3', 5', $J$ = 8.8), 7.05-6.93 (m, 2H, 3, 4), 6.47 (d, 1H, 21, $J$ = 2.1), 6.28 (d, 1H, 19, $J$ = 2.1), 6.15 (d, 1H, 12, $J$ = 8.8), 3.82 (s, 3H, 22), 3.69-3.61 (m, 1H, 10), 3.24-3.18 (m, 2H, 7), 1.74-1.64, 1.63-1.52 (2m, 4H, 8, 9), 1.22 (d, 3H, 11, $J$ = 6.3) | 163.34 (5), 162.21 (2), 159.00 (20), 158.26 (4', d, $J$ = 239.99), 144.63 (13), 144.23 (15), 135.22 (1'), 134.79 (17), 134.53 (14), 133.98 (3), 132.52 (4), 129.58 (18), 122.09 (16), 121.05 (2', 6', d, $J$ = 7.6), 115.44 (3', 5', d, $J$ = 21.89), 96.14 (21), 91.62 (19), 54.96 (22), 46.97 (10), 38.95 (7), 33.45 (9), 25.79 (8), 20.21 (11) |
| **4c** | 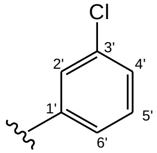 | 10.59 (s, 1H, 1), 8.54-8.53 (dd, 1H, 15, $J$ = 1.5, 4.1), 8.50 (t, 1H, 6, $J$ = 5.5), 8.08-8.06 (dd, 1H, 17, $J$ = 1.4, 8.2), 7.90 (s, 1H, 2'), 7.51 (d, 1H, 6', $J$ = 8.1), 7.43-7.41 (m, 1H, 16), 7.37 (t, H, 5', $J$ = 8.1), 7.16-7.14 (dd, 1H, 4′, $J$ = 1.2, $J$ = 8.0), 7.00 (q, 2H, 3, 4, $J$ = 15.1), 6.47 (d, 1H, 21, $J$ = 2.4), 6.28 (d, 1H, 19, $J$ = 2.3), 6.14 (d, 1H, 12, $J$ = 8.8), 3.82 (s, 3H, 22), 3.67-3.63 (m, 1H, 10), 3.23-3.20 (m, 2H, 7), 1.73-1.67, 1.63-1.53 (2m, 4H, 8, 9), 1.22 (d, 3H, 11, $J$ = 6.3) | 163.21 (5), 162.60 (2), 158.97 (20), 144.60 (13), 144.19 (15), 140.19 (1'), 134.74 (17), 134.50 (14), 134.41 (3), 133.10 (3'), 132.19 (4), 130.48 (5'), 129.54 (18), 123.45 (6'), 122.03 (16), 118.74 (2'), 117.72 (4'), 96.11 (21), 91.63 (19), 54.93 (22), 46.98 (10), 39.23 (7), 33.44 (9), 25.72 (8), 20.18 (11) |
| **4d** | 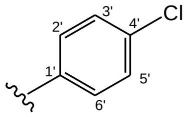 | 10.56 (s, 1H, 1), 8.55-8.53 (dd, 1H, 15, $J$ = 1.7, 4.2), 8.50 (t, 1H, 6, $J$ = 5.6), 8.09-8.06 (dd, 1H, 17, $J$ = 1.6, 8.3), 7.72-7.68 (m, 2H, 2', 6'), 7.45-7.37 (m, 3H, 16, 3', 5'), 7.06-6.94 (m, 2H, 3, 4), 6.47 (d, 1H, 21, $J$ = 2.5), 6.27 (d, 1H, 19, $J$ = 2.4), 6.14 (d, 1H, 12, $J$ = 8.8), 3.82 (s, 3H, 22), 3.69-3.60 (m, 1H, 10), 3.24-3.18 (m, 2H, 7), 1.74-1.64, 1.63-1.51 (2m, 4H, 8, 9), 1.22 (d, 3H, 11, $J$ = 6.3) | 163.29 (5), 162.43 (2), 159.00 (20), 144.63 (13), 144.23 (15), 137.76 (1'), 134.79 (17), 134.52 (14), 134.21 (3), 132.38 (4), 129.57 (18), 128.75 (3', 5'), 127.36 (4'), 122.09 (16), 120.84 (2', 6'), 96.14 (21), 91.61 (19), 54.96 (22), 46.97 (10), 38.82 (7), 33.44 (9), 25.77 (8), 20.21 (11) |

| | | | |
|---|---|---|---|
| **4e** | 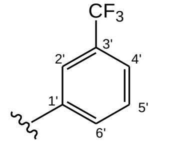 | 10.75 (s, 1H, 1), 8.55-8.52 (m, 2H, 6, 15), 8.18 (s, 1H, 2'), 8.08-8.06 (dd, 1H, 17, *J* = 1.1, 8.2), 7.83 (d, 1H, 6'), 7.59 (t, 1H, 5', *J* = 7.9), 7.45-7.42 (m, 2H, 16, 4′), 7.00-6.98 (q, 2H, 3, 4, *J* = 15.1), 6.47 (d, 1H, 21, *J* = 2.4), 6.28 (d, 1H, 19, *J* = 2.0), 6.15 (d, 1H, 12, *J* = 8.7), 3.82 (s, 3H, 22), 3.67-3.63 (m, 1H, 10), 3.23-3.20 (m, 2H, 7), 1.73-1.67, 1.63-1.53 (2m, 4H, 8, 9), 1.22 (d, 3H, 11, *J* = 6.3) | 163.19 (5), 162.81 (2), 158.99 (20), 144.62 (13), 144.21 (15), 139.54 (1'), 134.77 (17), 134.55 (14), 134.52 (3), 132.15 (4), 130.09 (5'), 129.56 (18), 129.82-129.19 (3', q, *J* = 31.7), 126.73-121.32 (7', q, *J* = 273.1), 122.88 (6'), 122.07 (16), 120.09 (4'), 115.33 (2'), 96.13 (21), 91.62 (19), 54.95 (22), 46.19 (10), 39.23 (7), 33.45 (9), 25.76 (8), 20.20 (11) |
| **4f** | 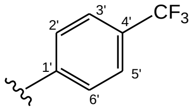 | 10.75 (s, 1H, 1), 8.54-8.53 (m, 1H, 15), 8.51 (t, 1H, 6, *J* = 5.3), 8.07 (d, 1H, 17, *J* = 8.2), 7.88 (d, 2H, 3', 5', *J* = 8.5), 7.71 (d, 2H, 2', 6', *J* = 8.5), 7.43-7.41 (m, 1H, 16), 7.07-6.99 (q, 2H, 3, 4, *J* = 15.1), 6.47 (d, 1H, 21, *J* = 2.2), 6.28 (d, 1H, 19, *J* = 2.2), 6.14 (d, 1H, 12, *J* = 8.7), 3.82 (s, 3H, 22), 3.67-3.63 (m, 1H, 10), 3.23-3.20 (dd, 2H, 7, *J* = 6.1, 12.1,), 2.59 (t, 2H, 3, *J* = 7.0), 1.73-1.67, 1.61-1.53 (2m, 4H, 8, 9), 1.22 (d, 3H, 1, *J* = 6.3) | 161.17 (5), 162.83 (2), 158.97 (20), 144.60 (13), 144.17 (15), 142.28 (1'), 134.72 (17), 134.66 (3), 134.50 (14), 132.11 (4), 129.53 (18), 126.95-121.55 (7', q, *J* = 271.6), 126.11-126.04 (3', 5', q, *J* = 3.0), 124.02-123.39 (4', q, *J* = 31.7), 122.02 (16), 119.26 (2', 6'), 96.10 (21), 91.63 (19), 54.92 (22), 46.96 (10), 38.80 (7), 33.43 (9), 25.70 (8), 20.17 (11) |
| **5a** | 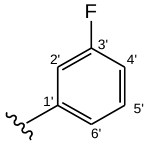 | 10.13 (s, 1H, 1), 8.54-8.53 (dd, 1H, 15, *J* = 1.7, 4.2), 8.08-8.06 (dd, 1H, 17, *J* = 1.6, 8.3), 7.86 (t, 1H, 6, *J* = 5.6), 7.60-7.58 (m, 1H, 6'), 7.43-7.41 (m, 1H, 16), 7.31-7.27 (m, 2H, 2', 5'), 6.85-6.81 (m, 1H, 4′), 6.47 (d, 1H, 21, *J* = 2.5), 6.26 (d, 1H, 19, *J* = 2.5), 6.11 (d, 1H, 12, *J* = 8.7), 3.82 (s, 3H, 22), 3.64-3.59 (m, 1H, 10), 3.10-3.03 (m, 2H, 7), 2.55 (t, 2H, 3, *J* = 7.2), 2.37 (t, 2H, 4, *J* = 7.2), 1.67-1.63, 1.55-1.45 (2m, 4H, 8, 9), 1.19 (d, 3H, 11, *J* = 6.3) | 170.86 (5, 2), 162.10 (3', d, *J* = 240.06), 158.98 (20), 144.61 (13), 144.19 (15), 141.03 (1', d, *J* = 13.1), 134.75 (17), 134.51 (14), 130.19 (5', d, *J* = 9.0), 129.55 (18), 122.05 (16), 114.55 (6'), 109.22 (2', d, *J* = 20.1), 105.61 (4', d, *J* = 23.5), 96.07 (21), 91.59 (19), 54.94 (22), 46.99 (10), 38.46 (7), 33.38 (9), 31.75 (3), 30.16 (4), 25.95 (8), 20.15 (11) |
| **5b** | 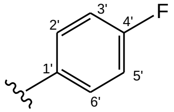 | 9.98 (s, 1H, 1), 8.55-8.53 (dd, 1H, 15, *J* = 1.6, 4.2), 8.09-8.06 (dd, 1H, 17, *J* = 1.5, 8.3), 7.87 (t, 1H, 6, *J* = 5.5), 7.62-7.57 (m, 2H, 2', 6'), 7.45-7.40 (m, 1H, 16), 7.14-7.08 (m, 2H, 3', 5'), 6.47 (d, 1H, 21, *J* = 2.4), 6.26 (d, 1H, 19, *J* = 2.4), 6.12 (d, 1H, 12, *J* = 8.8), 3.82 (s, 3H, 22), 3.65-3.57 (m, 1H, 10), 3.10-3.04 (m, 2H, 7), 2.54 (t, 2H, 3, *J* = 7.1), 2.37 (t, 2H, 4, *J* = 7.0), 1.69-1.59, 1.58-1.43 (2m, 4H, 8, 9), 1.19 (d, 3H, 11, *J* = 6.3) | 170.96 (5), 170.35 (2), 159.00 (20), 157.72 (4', d, *J* = 246.3), 144.63 (13), 144.22 (15), 135.76 (1'), 134.79 (17), 134.52 (14), 129.57 (18), 122.09 (16), 120.53 (2', 6', d, *J* = 7.8), 115.14 (3', 5', d, *J* = 22.1), 96.09 (21), 91.58 (19), 54.97 (22), 47.00 (10), 38.48 (7), 33.39 (9), 31.66 (3), 30.34 (4), 25.99 (8), 20.17 (11) |
| **5c** | 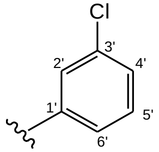 | 10.11 (s, 1H, 1), 8.54-8.53 (dd, 1H, 15, *J* = 1.6, 4.2), 8.08-8.06 (dd, 1H, 17, *J* = 1.6, 8.3), 7.86 (t, 1H, 6, *J* = 5.5), 7.81 (t, 1H, 2', *J* = 2.0), 7.43-7.41 (m, 2H, 16, 6′), 7.31-7.28 (m, 1H, 5'), 7.07-7.05 (m, 1H, 4'), 6.47 (d, 1H, 21, *J* = 2.5), 6.25 (d, 1H, 19, *J* = 2.5), 6.11 (d, 1H, 12, *J* = 8.2), 3.82 (s, 3H, 22), 3.63-3.59 (m, 1H, 10), 3.10-3.03 (m, 2H, 7), 2.55 (t, 2H, 3, *J* = 7.1), 2.37 (t, 2H, 4, *J* = 7.2), 1.67-1.63, 1.56-1.44 (2m, 4H, 8, 9), 1.19 (d, 3H, 11, *J* = 6.3) | 170.89 (5), 170.85 (2), 158.98 (20), 144.60 (13), 144.19 (15), 140.73 (1'), 134.77 (17), 134.49 (14), 132.97 (3'), 130.29 (5'), 129.55 (18), 122.51 (4'), 122.06 (16), 118.31 (6'), 117.19 (2'), 96.08 (21), 91.59 (19), 54.95 (22), 46.99 (10), 38.46 (7), 33.38 (9), 31.74 (3), 30.16 (4), 25.96 (8), 20.16 (11) |
| **5d** | 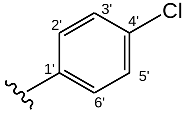 | 10.07 (s, 1H, 1), 8.55-8.53 (dd, 1H, 15, *J* = 1.5, 4.2), 8.10-8.07 (dd, 1H, 17, *J* = 1.4, 8.3), 7.88 (t, 1H, 6, *J* = 5.4), 7.61 (d, 2H, 2', 6', *J* = 8.9), 7.45-7.41 (m, 1H, 16), 7.32 (d, 2H, 3', 5', *J* = 8.9), 6.48 (d, 1H, 21, *J* = 2.3), 6.26 (d, 1H, 19, *J* = 2.3), 6.13 (bs, 1H, 12), 3.81 (s, 3H, 22), 3.63-3.57 (m, 1H, 10), 3.09-3.03 (m, 2H, 7), 2.55 (t, 2H, 3, *J* = 7.4), 2.39 (t, 2H, 4, *J* = 7.0), 1.70-1.59, 1.58-1.43 (2m, 4H, 8, 9), 1.19 (d, 3H, 11, *J* = 6.3) | 170.92 (5), 170.64 (2), 159.01 (20), 144.55 (13), 144.18 (15), 138.28 (1'), 134.89 (17), 134.42 (14), 129.59 (18), 128.51 (3', 5'), 126.33 (4'), 122.09 (16), 120.37 (2', 6'), 96.19 (21), 91.63 (19), 54.98 (22), 47.02 (10), 38.47 (7), 33.37 (9), 31.73 (3), 30.23 (4), 25.98 (8), 20.15 (11) |

| | | | |
|---|---|---|---|
| **5e** | 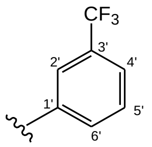 | 10.26 (s, 1H, 1), 8.54-8.53 (dd, 1H, 15, *J* = 1.6, 4.2), 8.11 (s, 1H, 2'), 8.08-8.06 (dd, 1H, 17, *J* = 1.6, 8.3), 7.88 (t, 1H, 6, *J* = 5.5), 7.74 (d, 1H, 6', *J* = 8.1), 7.52 (t, 1H, 4', *J* = 8.0), 7.43-7.41 (m, 1H, 16), 7.36 (d, 1H, 5′, *J* = 7.8), 6.47 (d, 1H, 21, *J* = 2.5), 6.25 (d, 1H, 19, *J* = 2.5), 6.11 (d, 1H, 12, *J* = 8.7), 3.82 (s, 3H, 22), 3.63-3.58 (m, 1H, 10), 3.10-3.04 (m, 2H, 7), 2.57 (t, 2H, 3, *J* = 7.2), 2.41 (t, 2H, 4, *J* = 7.2), 1.67-1.63, 1.56-1.44 (2m, 4H, 8, 9), 1.19 (d, 3H, 11, *J* = 6.3) | 171.11 (5), 170.84 (2), 158.98 (20), 144.61 (13), 144.19 (15), 140.03 (1'), 134.75 (17), 134.50 (14), 129.83 (5'), 129.55 (18), 129.67-129.04 (3', q, *J* = 31.9), 126.81-121.40 (7', q, *J* = 269.8), 122.33 (6'), 122.05 (16), 119.14 (4'), 114.88 (2'), 96.06 (21), 91.58 (19), 54.94 (22), 46.98 (10), 38.46 (7), 33.38 (9), 31.72 (3), 30.11 (4), 25.96 (8), 20.14 (11) |
| **5f** | 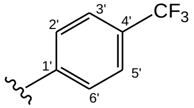 | 10.31 (s, 1H, 1), 8.54-8.52 (dd, 1H, 15, *J* = 1.5, 4.2), 8.09-8.06 (dd, 1H, 17, *J* = 1.5, 8.3), 7.89 (t, 1H, 6, *J* = 5.3), 7.79 (d, 2H, 2', 6', *J* = 8.5), 7.63 (d, 2H, 3', 5', *J* = 8.7), 7.44-7.40 (m, 1H, 16), 6.47 (d, 1H, 21, *J* = 2.4), 6.25 (d, 1H, 19, *J* = 2.4), 6.11 (d, 1H, 12, *J* = 8.7), 3.82 (s, 3H, 22), 3.65-3.57 (m, 1H, 10), 3.09-3.04 (m, 2H, 7), 2.59 (t, 2H, 3, *J* = 7.0), 2.40 (t, 2H, 4, *J* = 7.0), 1.71-1.58, 1.57-1.43 (2m, 4H, 8, 9), 1.19 (d, 3H, 11, *J* = 6.2) | 171.18 (5), 170.86 (2), 158.99 (20), 144.61 (13), 144.20 (15), 142.83 (1'), 134.77 (17), 134.51 (14), 129.55 (18), 125.95 (3', 5'), 127.05-121.68 (7', q, *J* = 274.2), 123.15-122.52 (4', q, *J* = 28.2), 122.06 (16), 118.69 (2', 6'), 96.08 (21), 91.59 (19), 54.95 (22), 46.99 (10), 38.47 (7), 33.38 (9), 31.79 (3), 30.10 (4), 25.96 (8), 20.14 (11) |

**Table S4.** Interaction of fumardiamide **4b** with GSH

| **Time (h)** | **Reaction** | | | **Control** | | |
|---|---|---|---|---|---|---|
| | **4b** | **standard** | **4b/standard** | **4b** | **standard** | **4b/standard** |
| 0 | 1.09E+09 | 1.49E+09 | 0.732 | 6.05E+08 | 1.26E+09 | 0.479 |
| 5 | 8.64E+08 | 1.21E+09 | 0.715 | 5.51E+08 | 1.07E+09 | 0.516 |
| 26 | 7.65E+08 | 1.09E+09 | 0.704 | 5.27E+08 | 1.03E+09 | 0.509 |
| 52 | 5.80E+08 | 8.71E+08 | 0.665 | 5.06E+08 | 1.02E+09 | 0.498 |
| 124 | 5.96E+08 | 9.29E+08 | 0.642 | 5.64E+08 | 1.16E+09 | 0.488 |
| 216 | 6.64E+08 | 1.11E+09 | 0.598 | 6.06E+08 | 1.21E+09 | 0.500 |

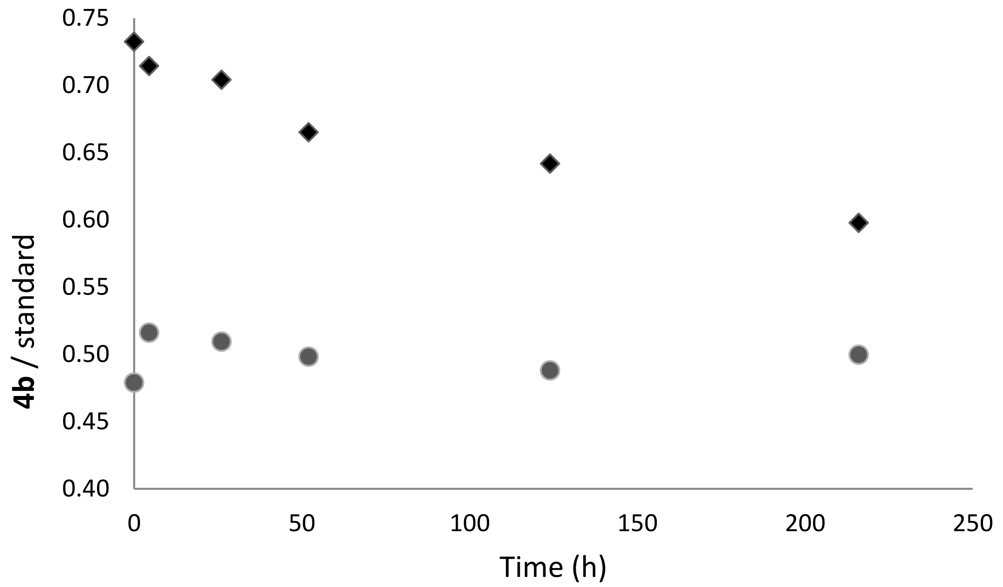


**Figure S1.** Interaction of fumardiamide **4b** with GSH (♦). Control: (*N*-(benzyloxy)-*N*'-{4-[(6-methoxyquinolin-8-yl)amino]pentyl}butanediamide) (•).

Spectra:

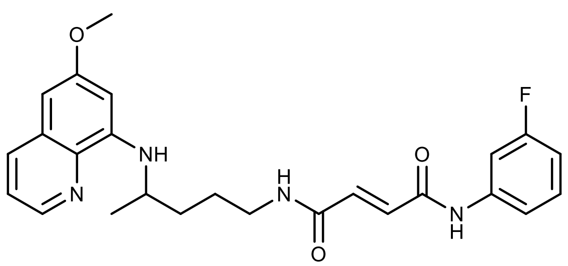

O
NH
N
H
N
O
O
N
H
F


**4a**

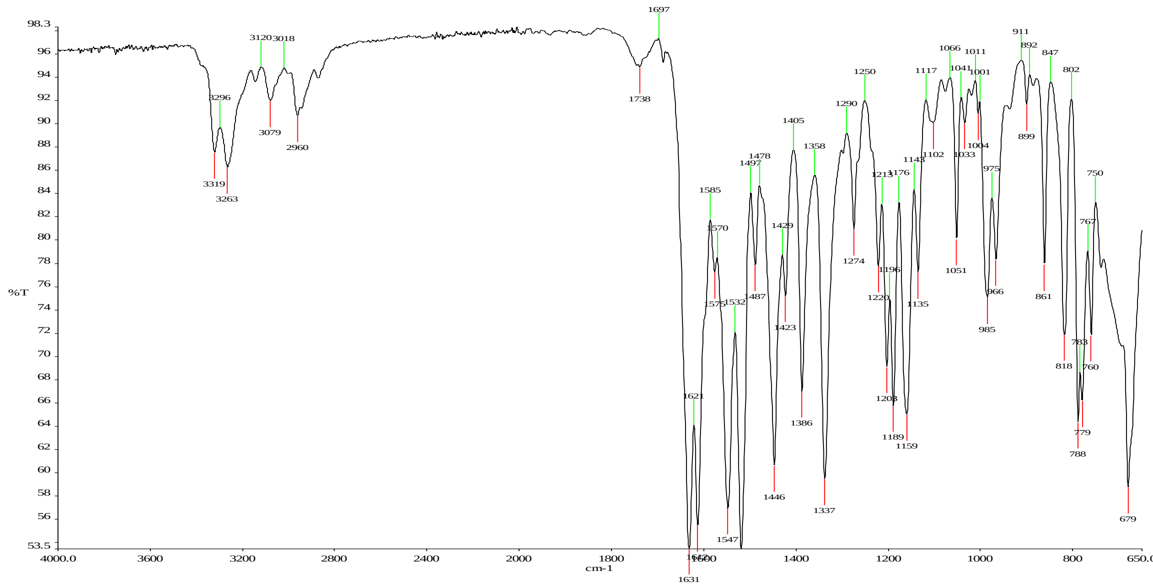

%T
cm-1
98.3
53.5
4000.0
3600
3200
2800
2400
2000
1800
1600
1400
1200
1000
800
650.0

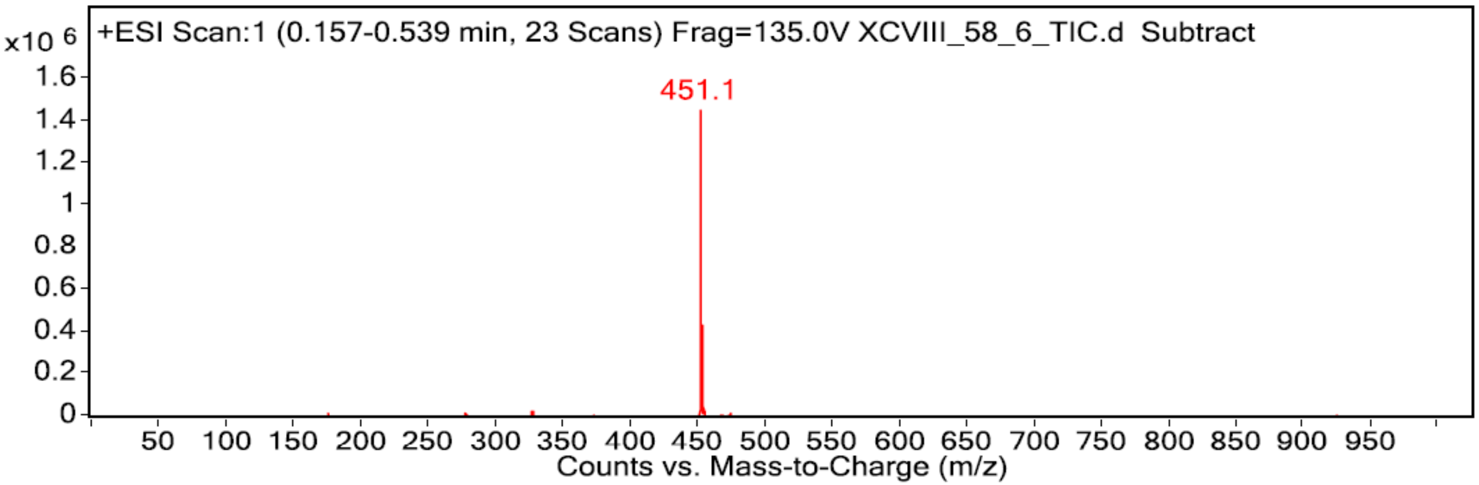
+ESI Scan:1 (0.157-0.539 min, 23 Scans) Frag=135.0V XCVIII_58_6_TIC.d Subtract
x10 6
451.1
Counts vs. Mass-to-Charge (m/z)

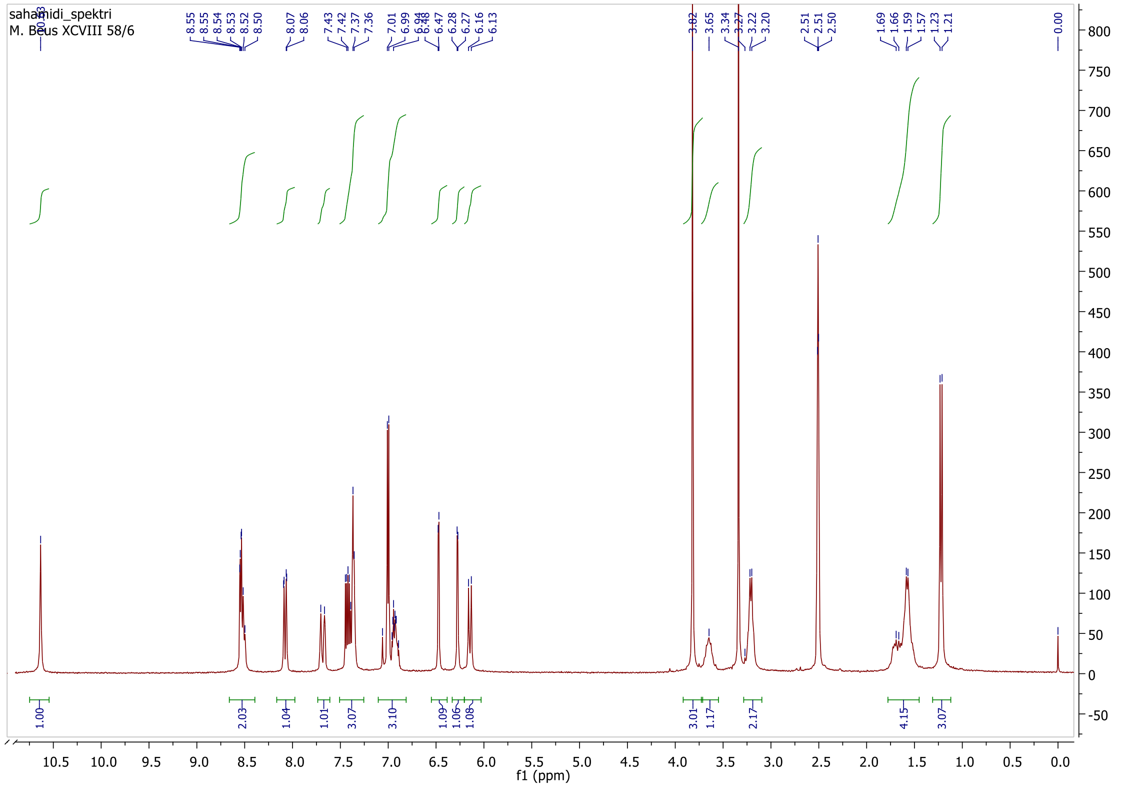
sahamidi_spektri
M. Beus XCVIII 58/6
f1 (ppm)

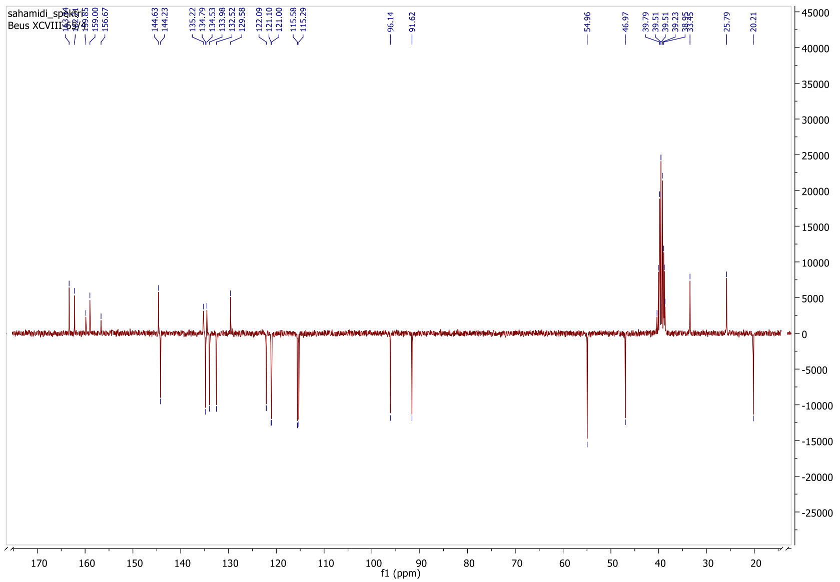

sahamidi_spektri
Beus XCVIII-65/4
159.85
159.00
156.67
144.63
144.23
135.22
134.79
134.53
133.98
132.52
129.58
122.09
121.10
121.00
115.58
115.29
96.14
91.62
54.96
46.97
39.79
39.51
39.51
39.23
38.95
33.45
25.79
20.21
f1 (ppm)

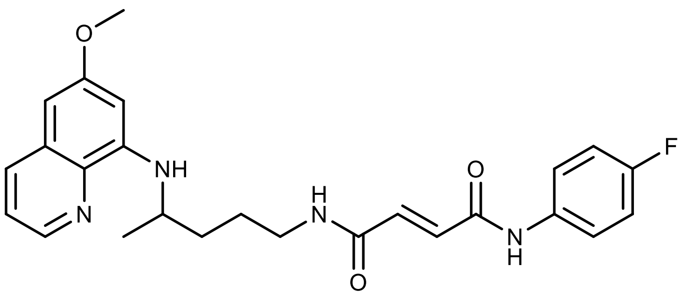

**4b**

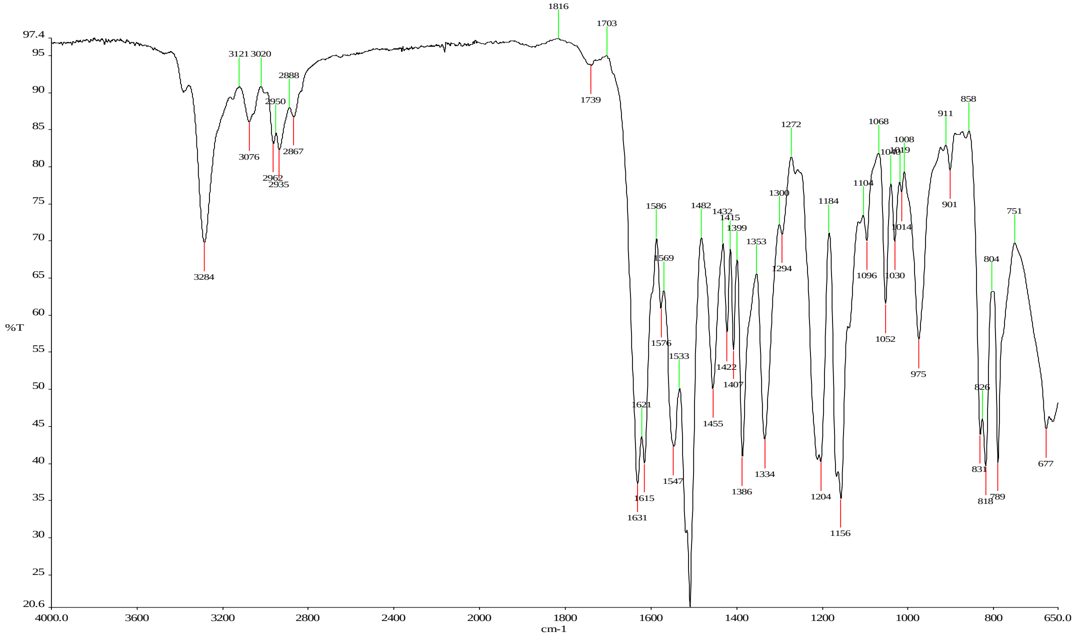

| Spectrum Source | Fragmentor Voltage | Collision Energy | Ionization Mode |
|---|---|---|---|
| Peak (1) in "+/- TIC Scan" | 135 | 0 | ESI |

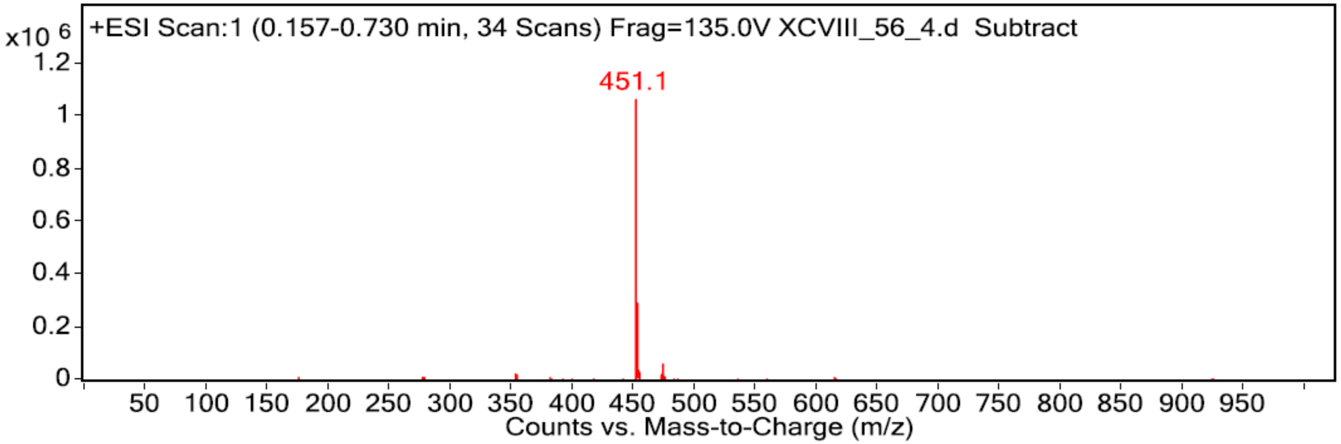


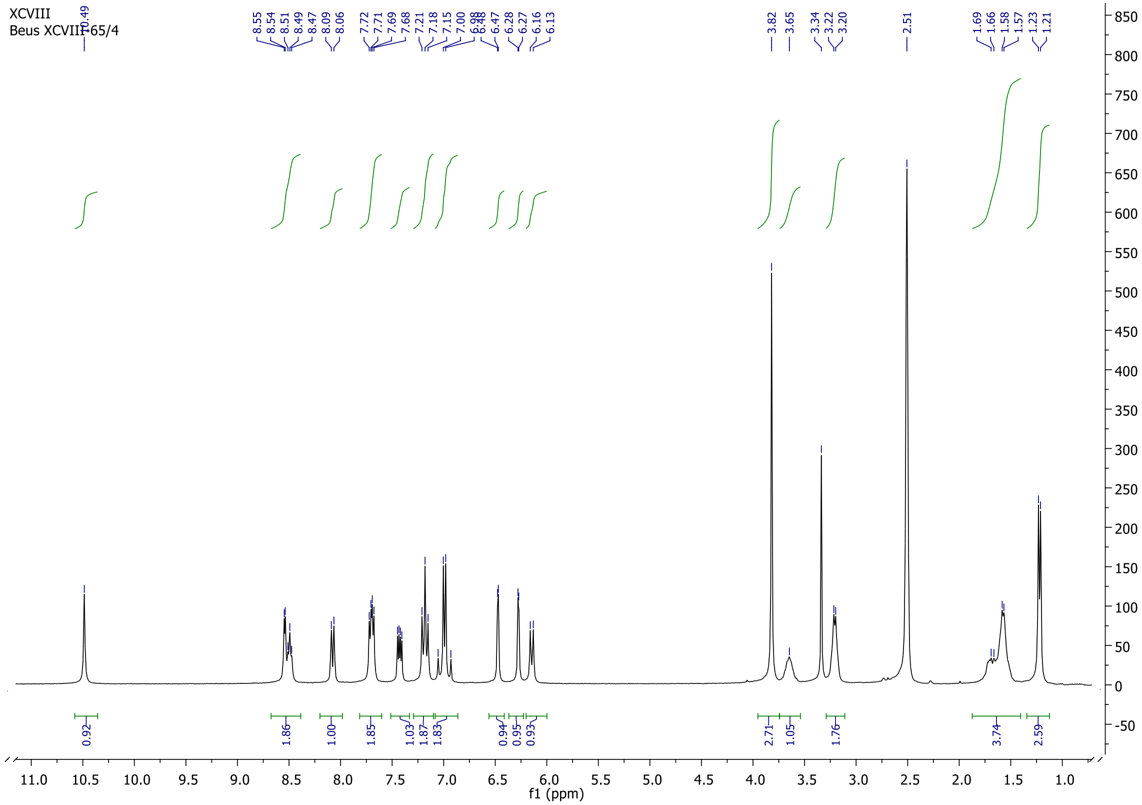

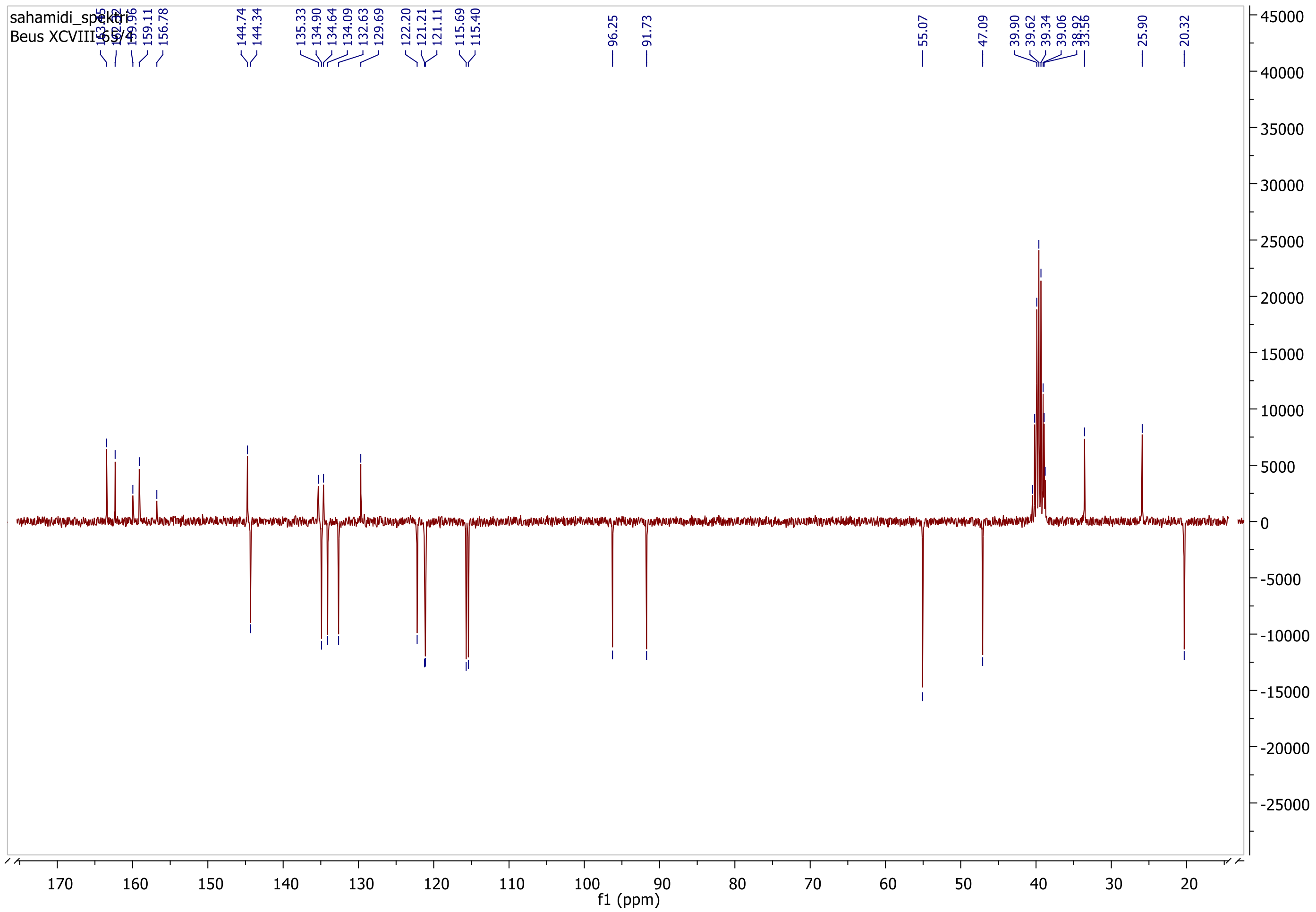
sahamidi_spektri
Beus XCVIII-65/4
159.11
156.78
144.74
144.34
135.33
134.90
134.64
134.09
132.63
129.69
122.20
121.21
121.11
115.69
115.40
96.25
91.73
55.07
47.09
39.90
39.62
39.34
39.06
38.92
33.56
25.90
20.32
45000
40000
35000
30000
25000
20000
15000
10000
5000
0
-5000
-10000
-15000
-20000
-25000
170
160
150
140
130
120
110
100
90
80
70
60
50
40
30
20
f1 (ppm)

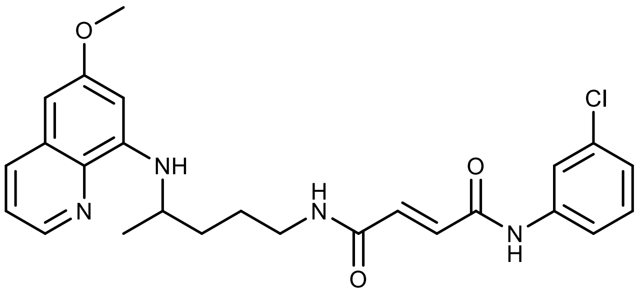
O
NH
N
H
N
O
O
N
H
Cl

**4c**

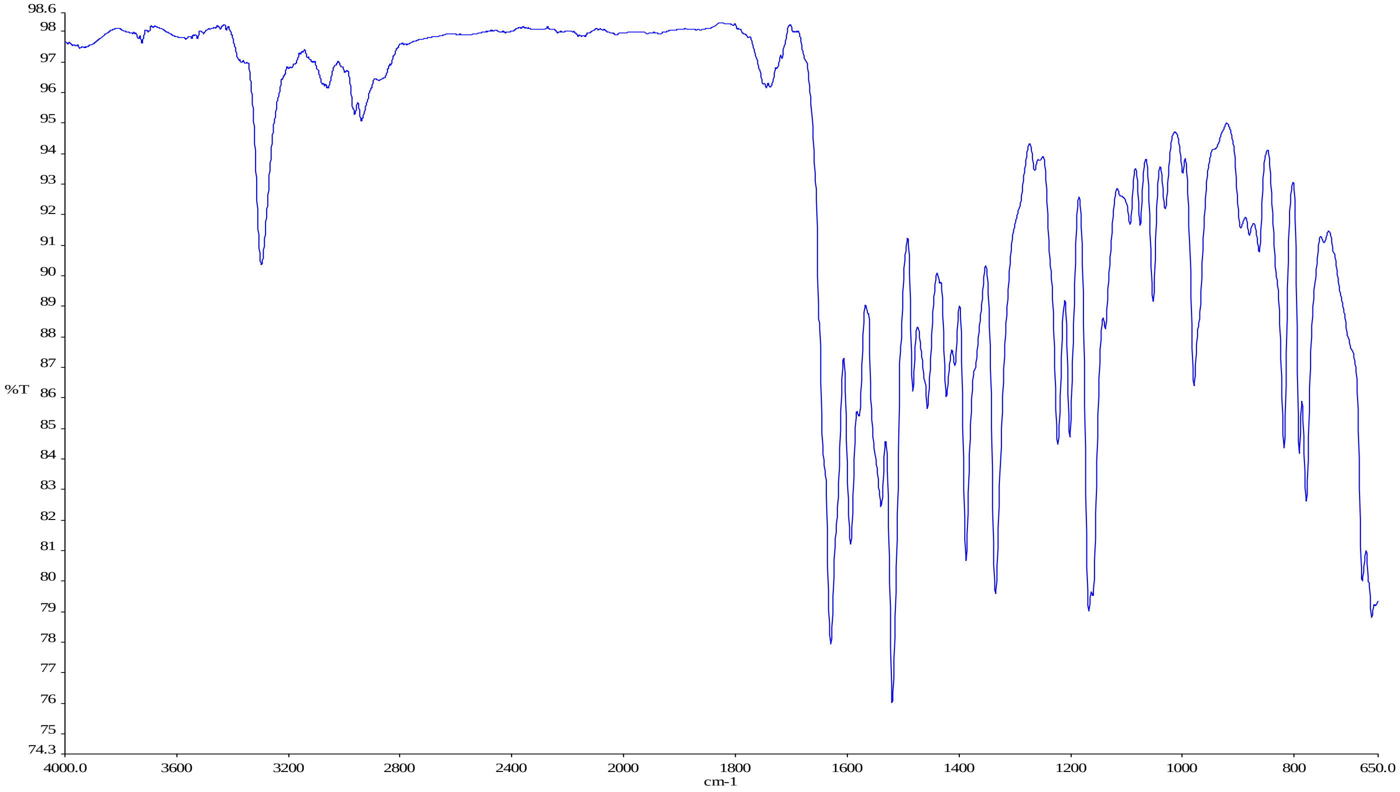
98.6
98
97
96
95
94
93
92
91
90
89
88
87
%T
86
85
84
83
82
81
80
79
78
77
76
75
74.3
4000.0
3600
3200
2800
2400
2000
1800
1600
1400
1200
1000
800
650.0
cm-1

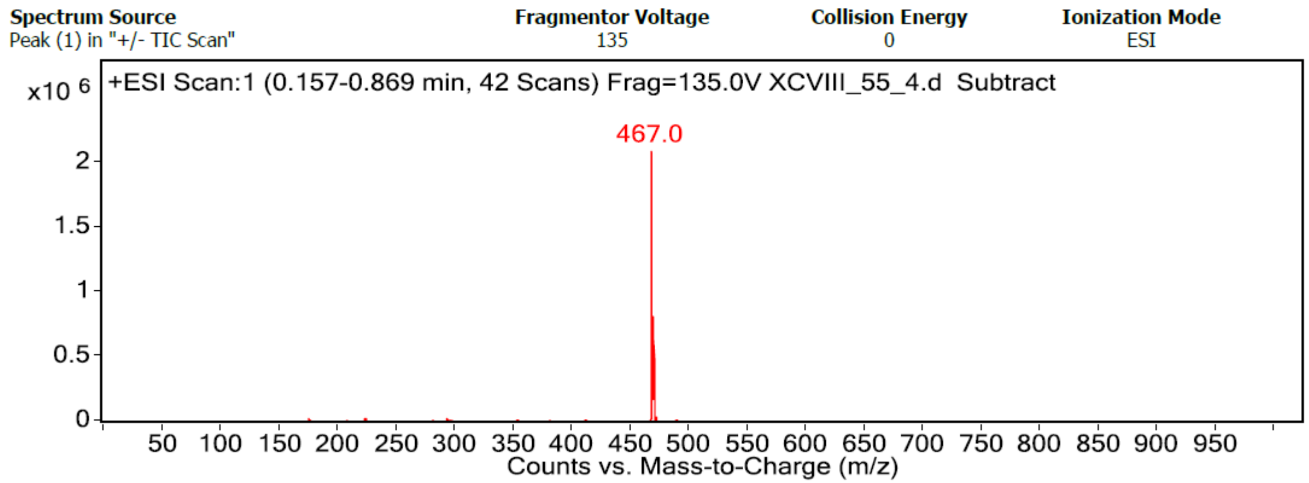
Spectrum Source
Peak (1) in "+/- TIC Scan"
Fragmentor Voltage
135
Collision Energy
0
Ionization Mode
ESI
x10 6
+ESI Scan:1 (0.157-0.869 min, 42 Scans) Frag=135.0V XCVIII_55_4.d Subtract
467.0
Counts vs. Mass-to-Charge (m/z)

S

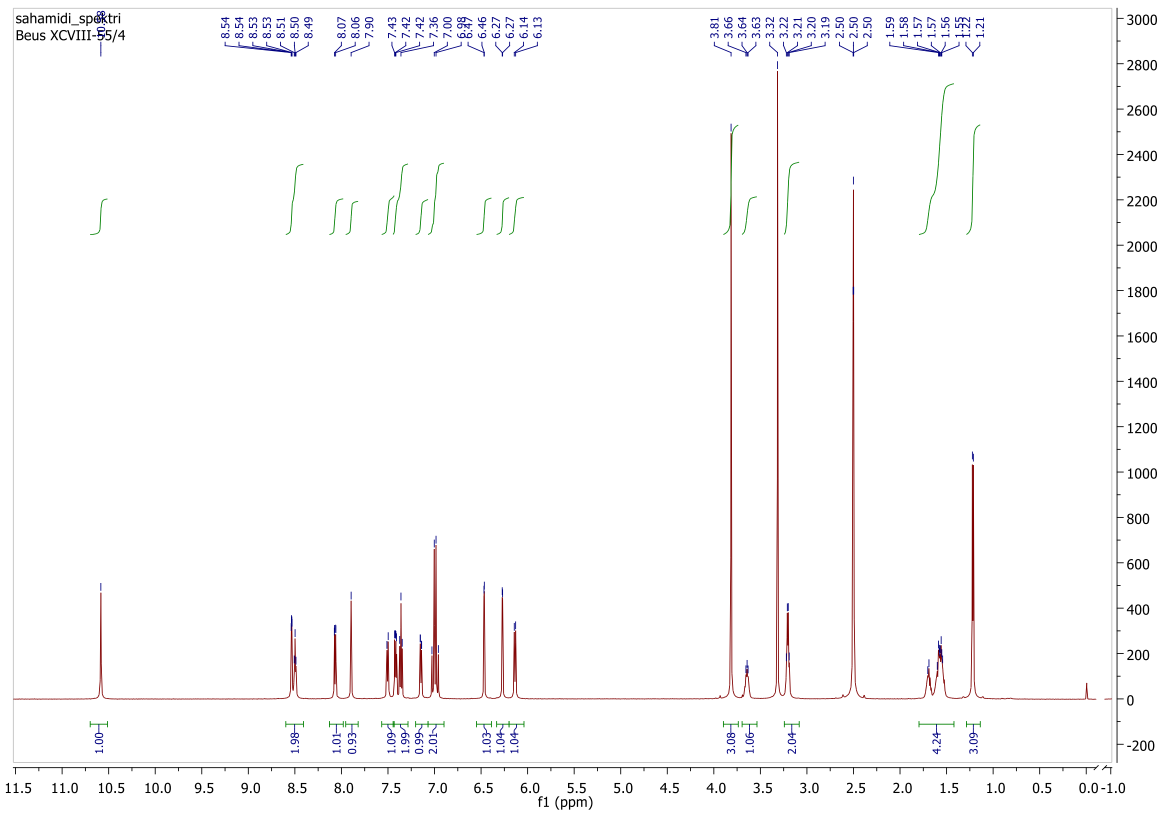
sahamidi_spektri
Beus XCVIII-55/4
f1 (ppm)

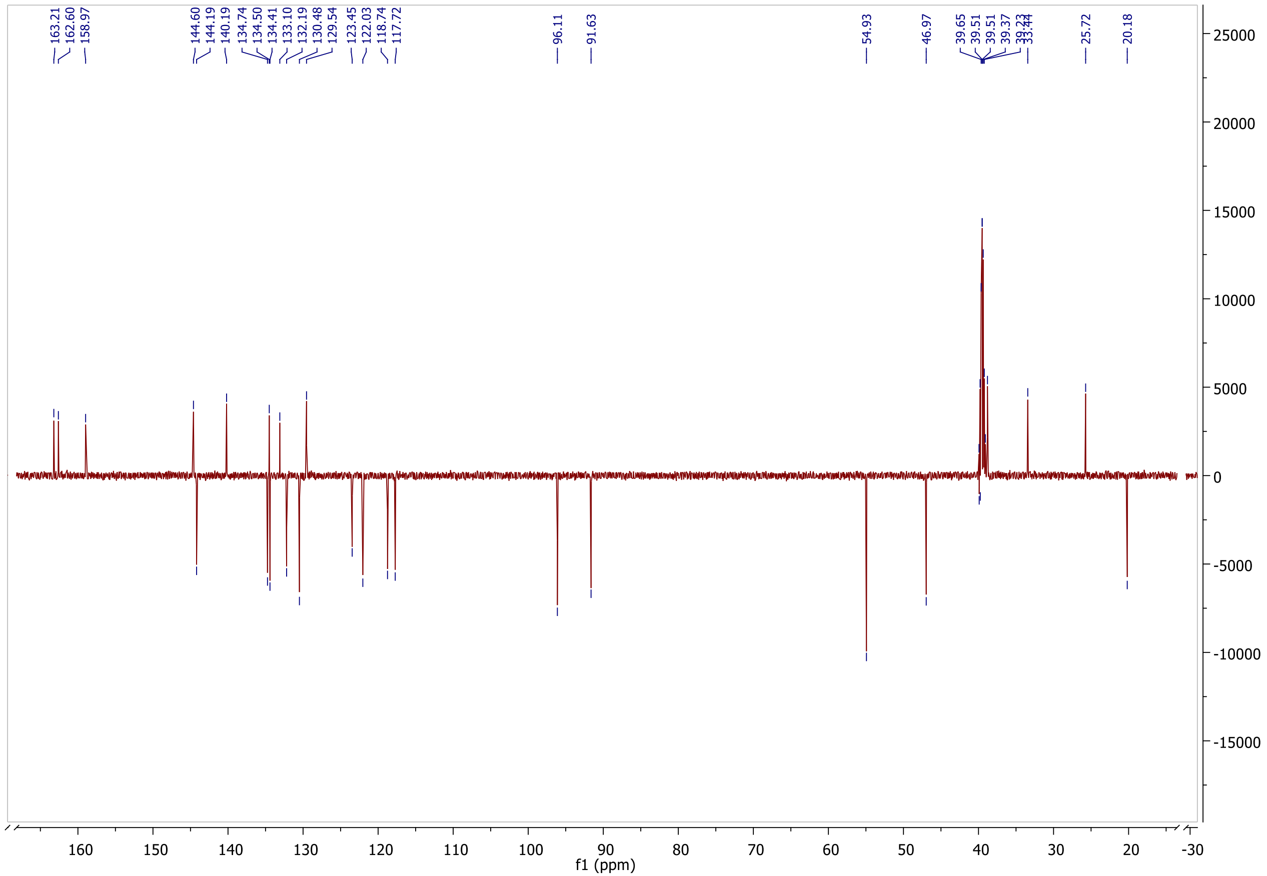
163.21
162.60
158.97
144.60
144.19
140.19
134.74
134.50
134.41
133.10
132.19
130.48
129.54
123.45
122.03
118.74
117.72
96.11
91.63
54.93
46.97
39.65
39.51
39.51
39.37
33.44
25.72
20.18
f1 (ppm)

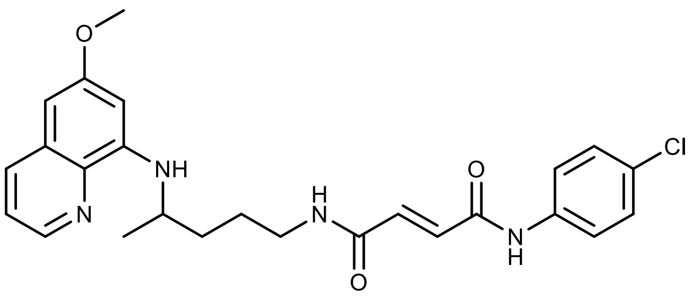
O
NH
N
H
N
O
O
N
H
Cl

**4d**

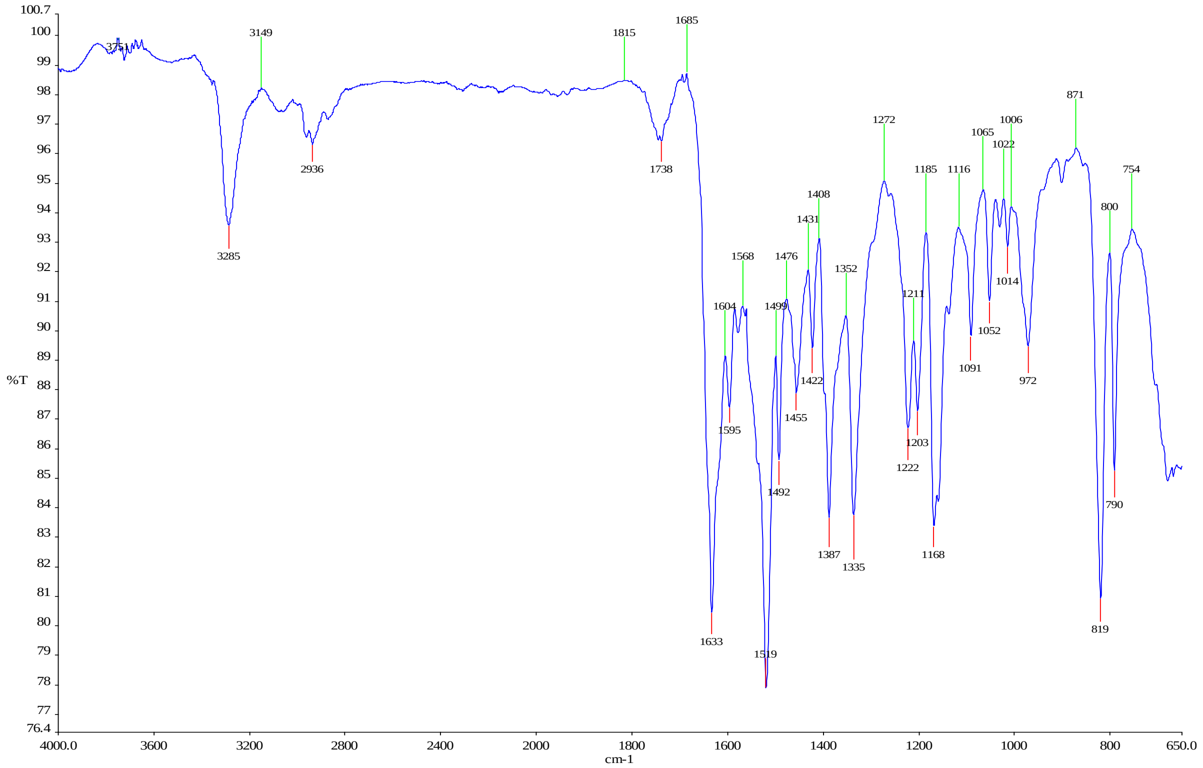
100.7
100
99
98
97
96
95
94
93
92
91
90
89
88
87
86
85
84
83
82
81
80
79
78
77
76.4
%T
4000.0
3600
3200
2800
2400
2000
1800
1600
1400
1200
1000
800
650.0
cm-1
3751
3149
3285
2936
1815
1685
1738
1633
1604
1595
1568
1519
1499
1492
1476
1455
1431
1422
1408
1387
1352
1335
1272
1222
1211
1203
1185
1168
1116
1091
1065
1052
1022
1014
1006
972
871
819
800
790
754

| Spectrum Source | Fragmentor Voltage | Collision Energy | Ionization Mode |
|---|---|---|---|
| Peak (1) in "+/- TIC Scan" | 135 | 0 | ESI |

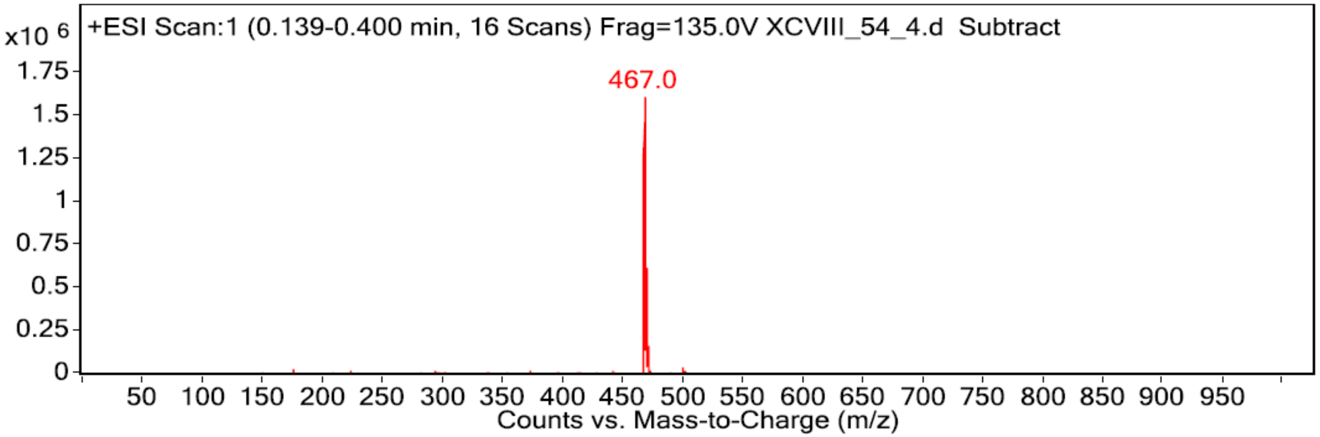


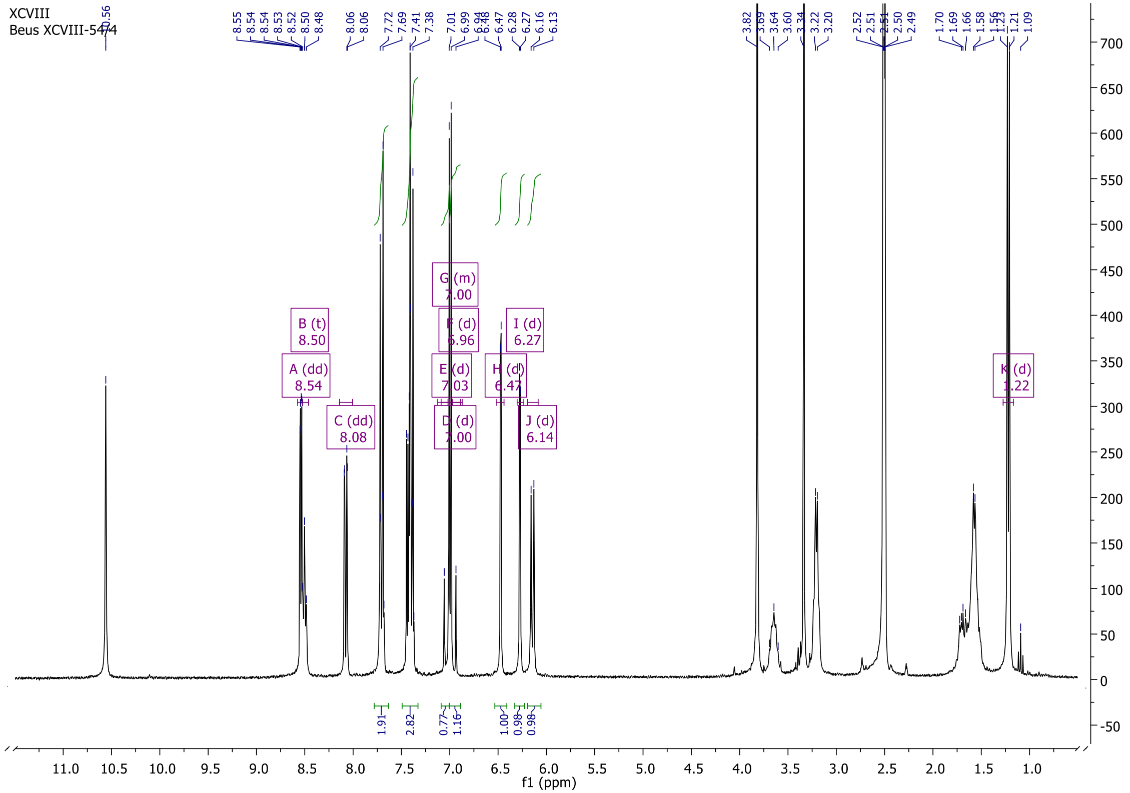

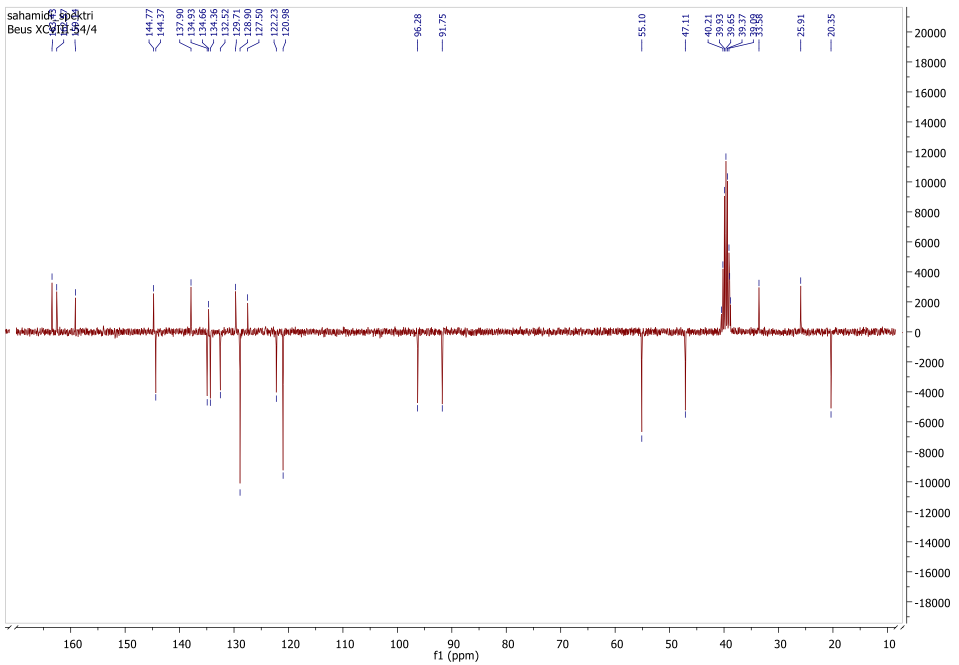
sahamidi_spektri
Beus XCVIII-54/4
144.77
144.37
137.90
134.93
134.66
134.36
132.52
129.71
128.90
127.50
122.23
120.98
96.28
91.75
55.10
47.11
40.21
39.93
39.65
39.37
39.09
33.58
25.91
20.35
f1 (ppm)

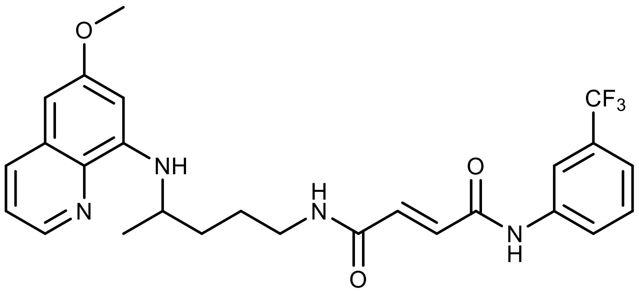


**4e**

| Spectrum Source | Fragmentor Voltage | Collision Energy | Ionization Mode |
|---|---|---|---|
| Peak (1) in "+/- TIC Scan" | 135 | 0 | ESI |

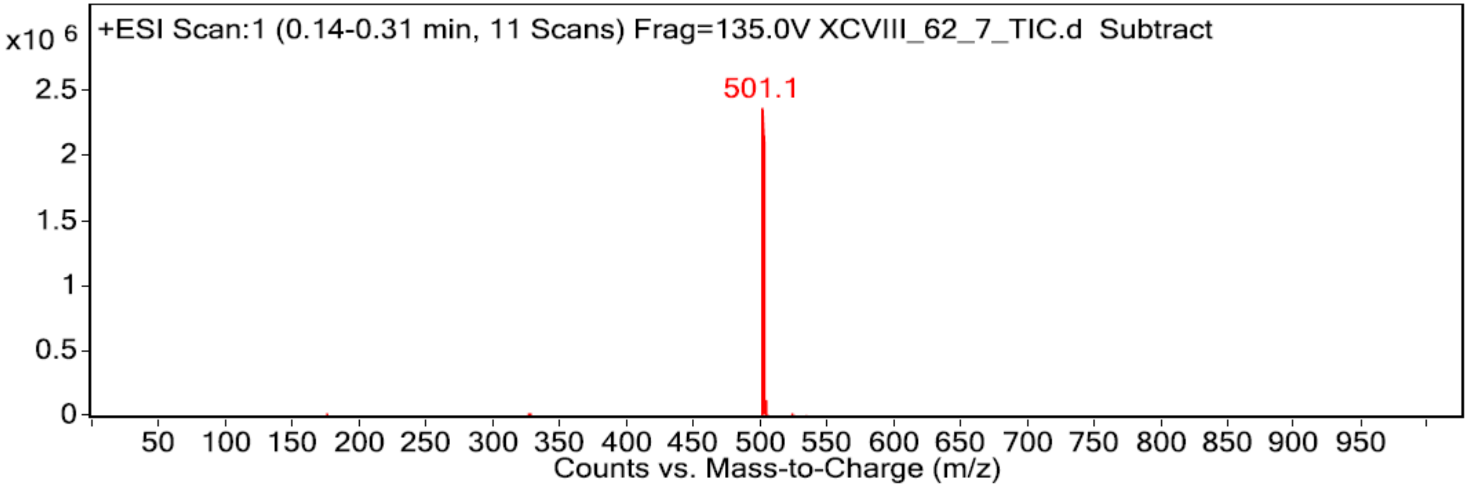

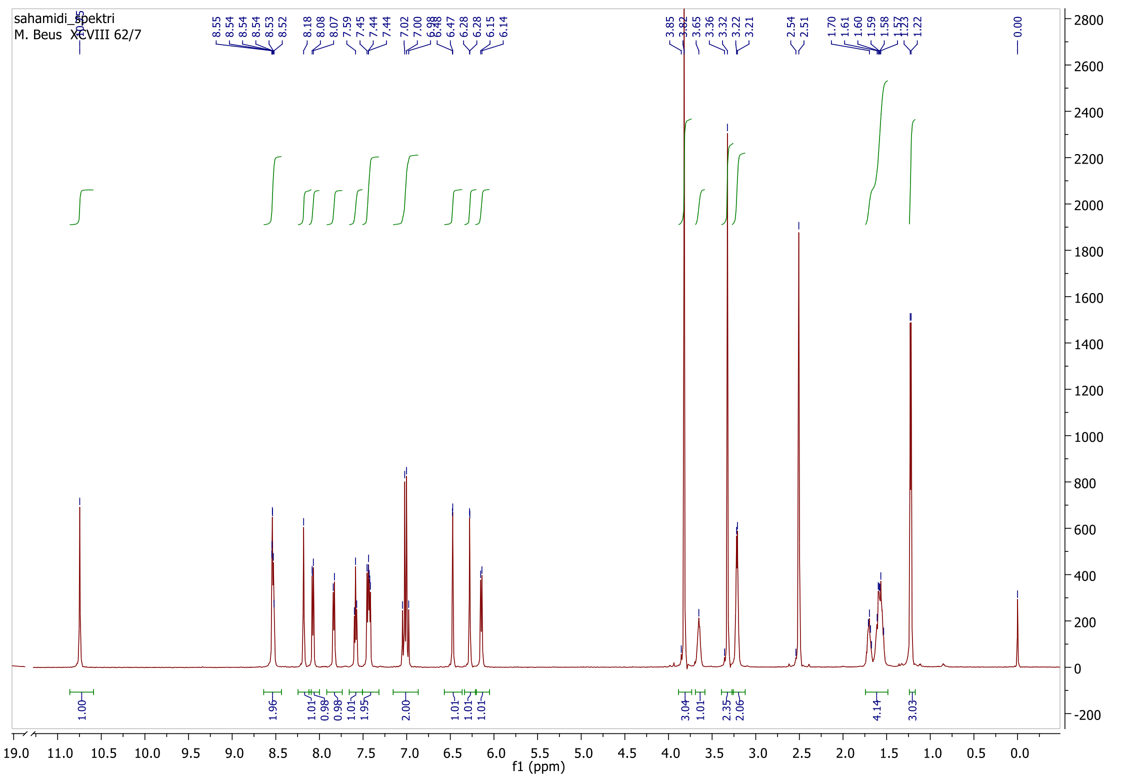

sahamidi_spektri
M. Beus XCVIII 62/7
8.55 8.54 8.54 8.54 8.53 8.52 8.18 8.08 8.07 7.59 7.45 7.44 7.44 7.02 7.00 6.98 6.98 6.47 6.28 6.28 6.15 6.14
3.85 3.82 3.65 3.36 3.32 3.22 3.21 2.54 2.51 1.70 1.61 1.60 1.59 1.58 1.57 1.23 1.22 0.00
1.00 1.96 1.01 0.98 0.98 1.01 1.95 2.00 1.01 1.01 1.01 3.04 1.01 2.35 2.06 4.14 3.03
f1 (ppm)

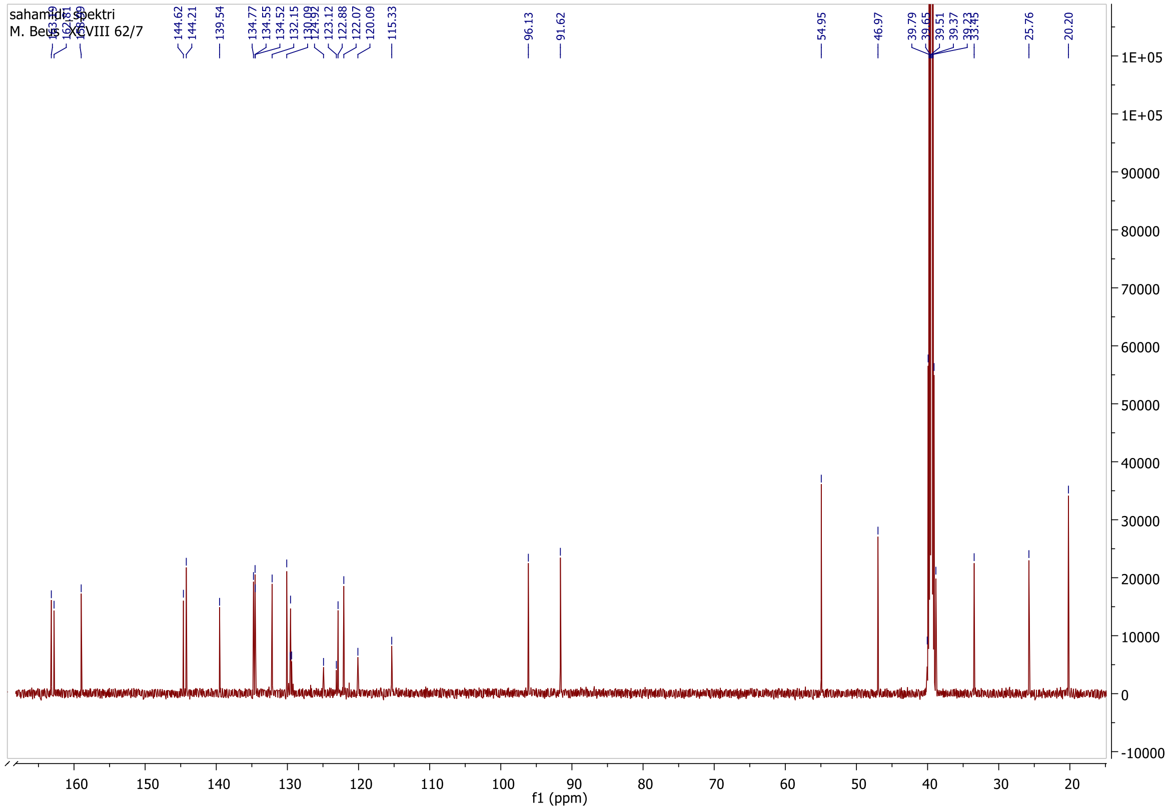

sahamidi_spektri
M. Beus XCVIII 62/7
162.81
144.62
144.21
139.54
134.77
134.55
134.52
132.15
130.09
124.92
123.12
122.88
122.07
120.09
115.33
96.13
91.62
54.95
46.97
39.79
39.65
39.51
39.37
39.23
33.45
25.76
20.20
f1 (ppm)

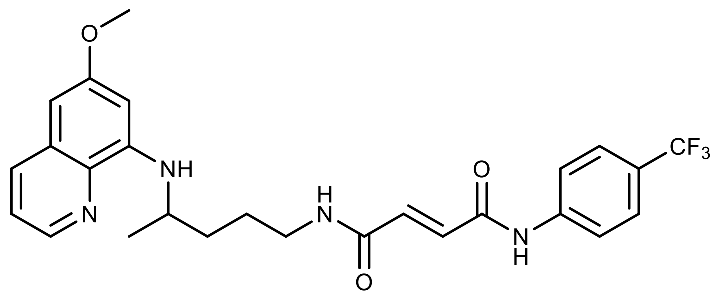

**4f**

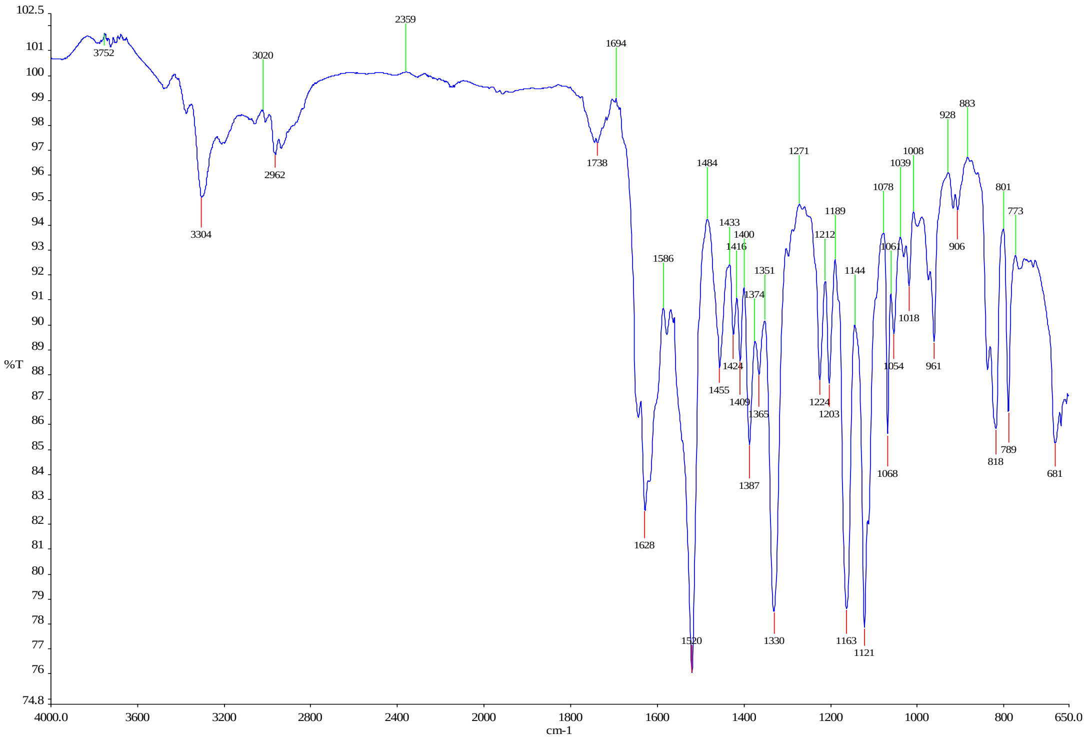

%T
cm-1
3752
3304
3020
2962
2359
1738
1694
1628
1586
1520
1484
1455
1433
1424
1416
1409
1400
1387
1374
1365
1351
1330
1271
1224
1212
1203
1189
1163
1144
1121
1078
1068
1061
1054
1039
1018
1008
961
928
906
883
818
801
789
773
681

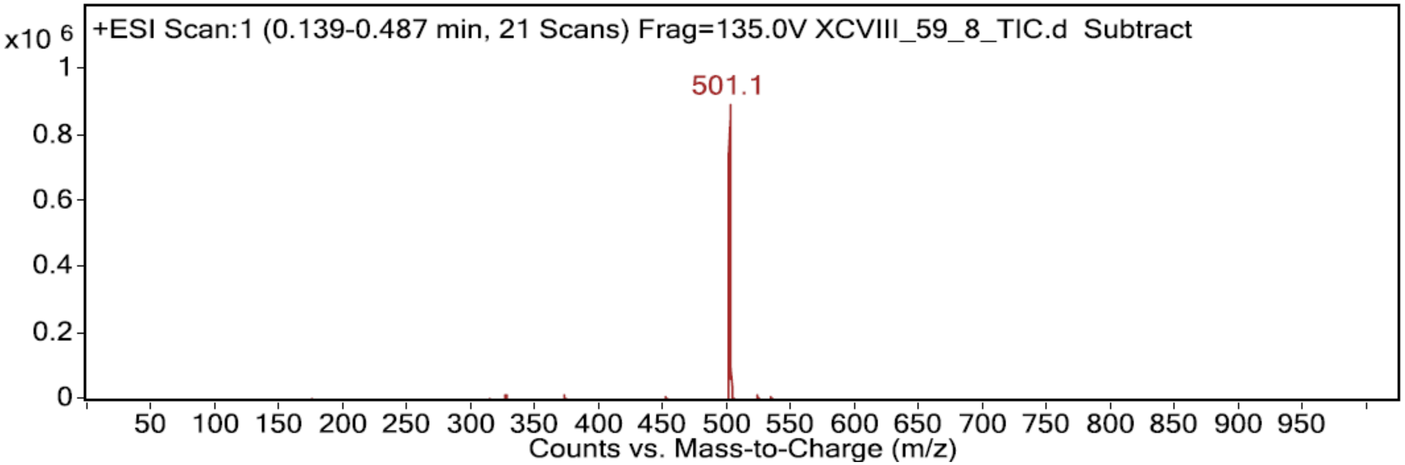
x10 6
+ESI Scan:1 (0.139-0.487 min, 21 Scans) Frag=135.0V XCVIII_59_8_TIC.d Subtract
501.1
Counts vs. Mass-to-Charge (m/z)

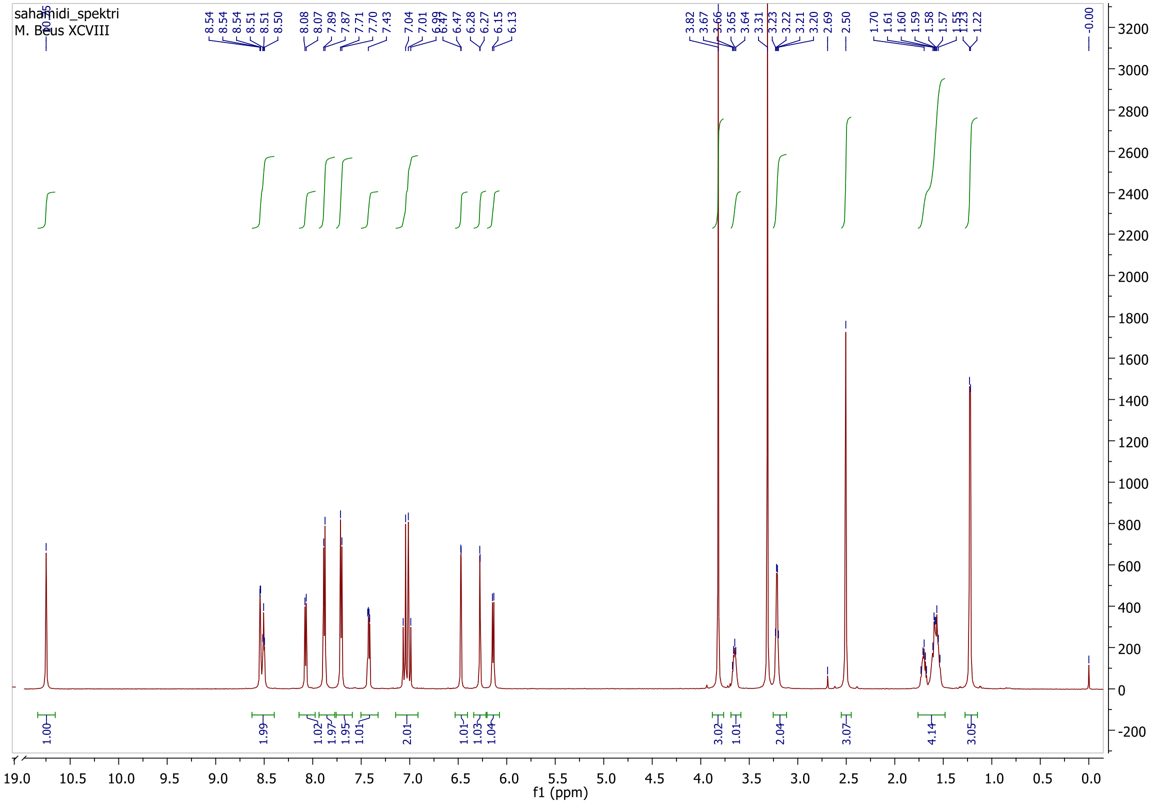
sahamidi_spektri
M. Beus XCVIII
f1 (ppm)

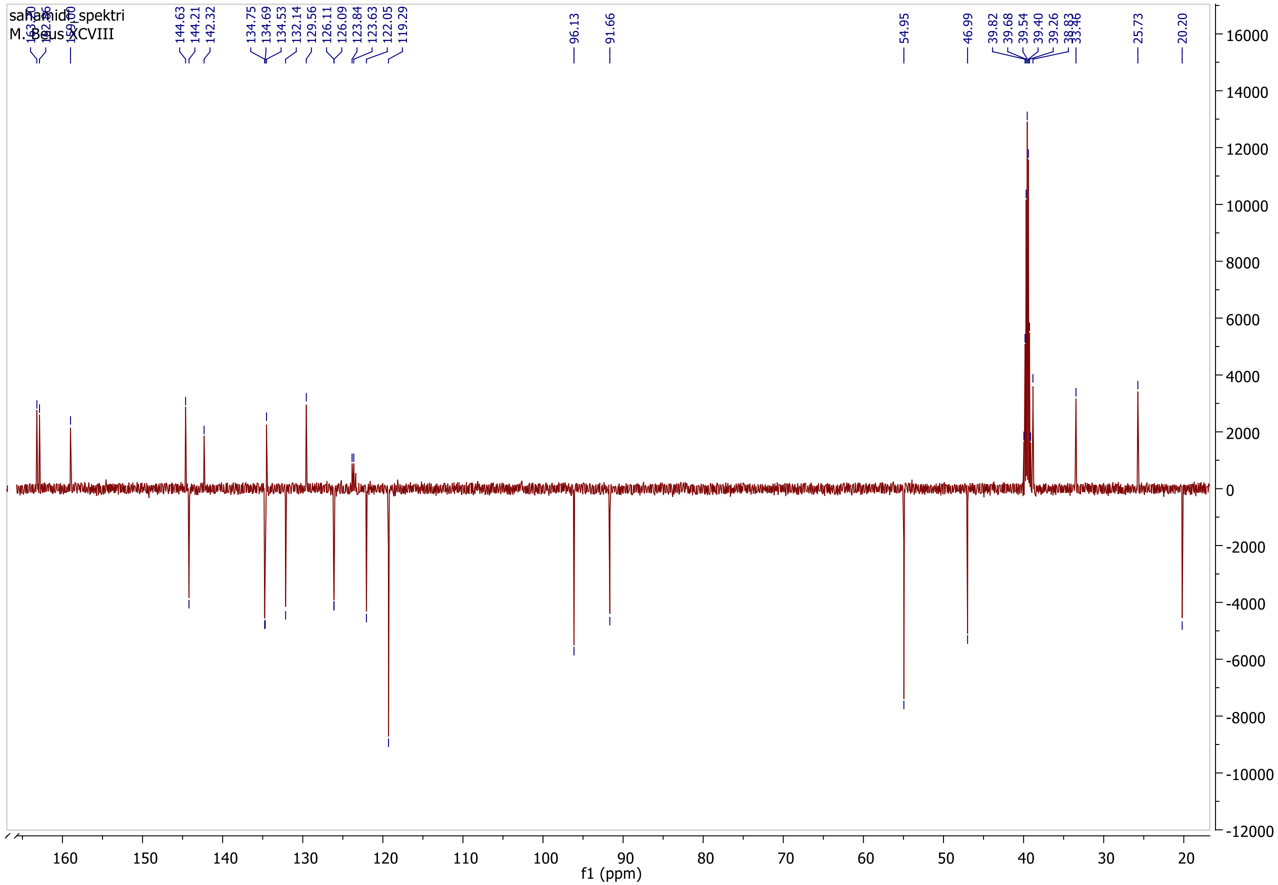

sahamidi_spektri
M. Beus XCVIII
163.20
144.63
144.21
142.32
134.75
134.69
134.53
132.14
129.56
126.11
126.09
123.84
123.63
122.05
119.29
96.13
91.66
54.95
46.99
39.82
39.68
39.54
39.40
39.26
33.46
25.73
20.20
16000
14000
12000
10000
8000
6000
4000
2000
0
-2000
-4000
-6000
-8000
-10000
-12000
160
150
140
130
120
110
100
90
80
70
60
50
40
30
20
f1 (ppm)

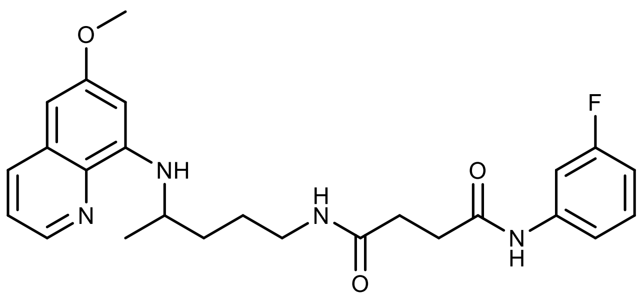


**5a**

Peak (1) in "+/- TIC Scan" 135 0 ESI

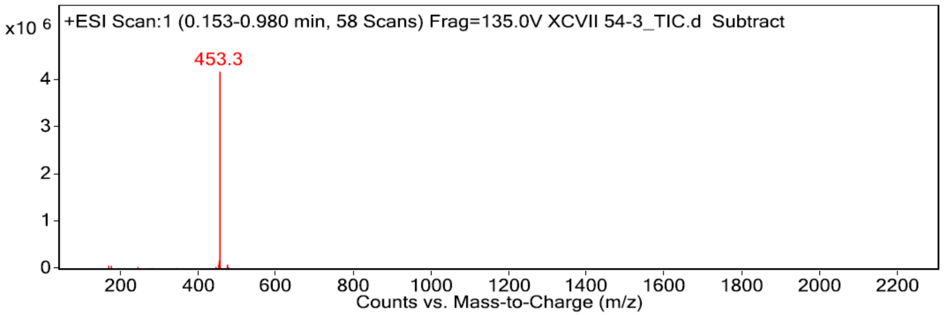


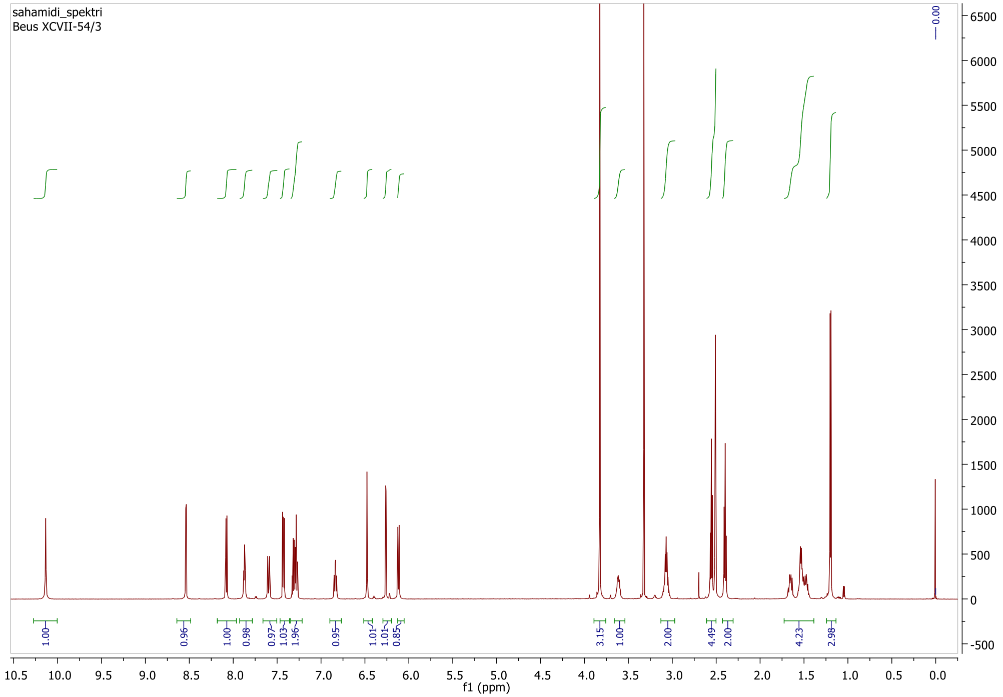

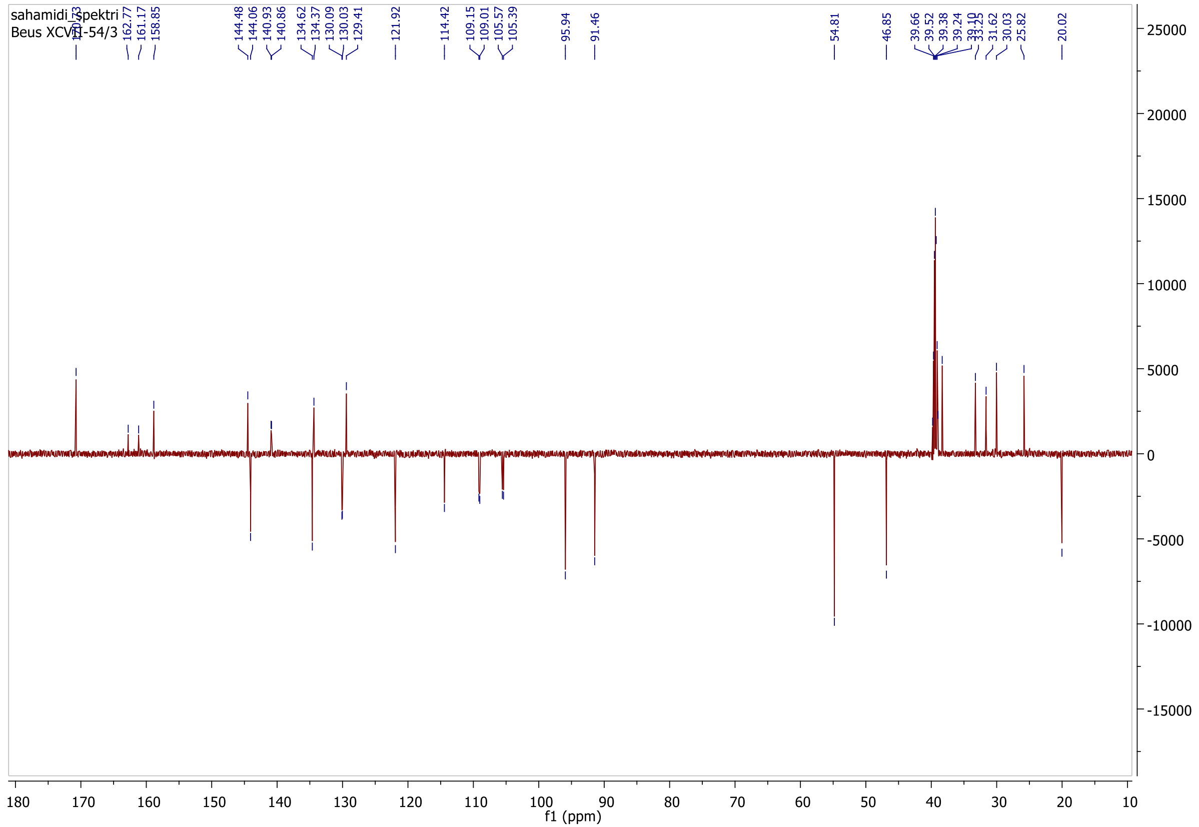

sahamidi spektri
Beus XCVII-54/3
f1 (ppm)

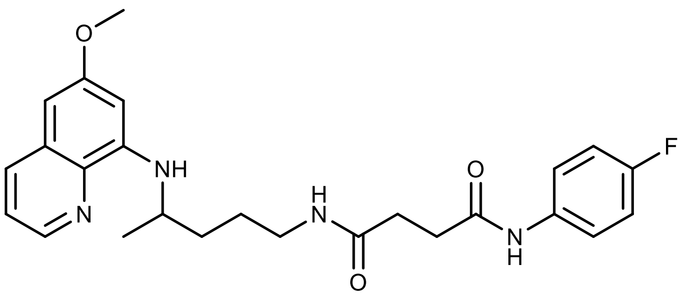
O
NH
N
H
N
O
O
N
H
F

**5b**

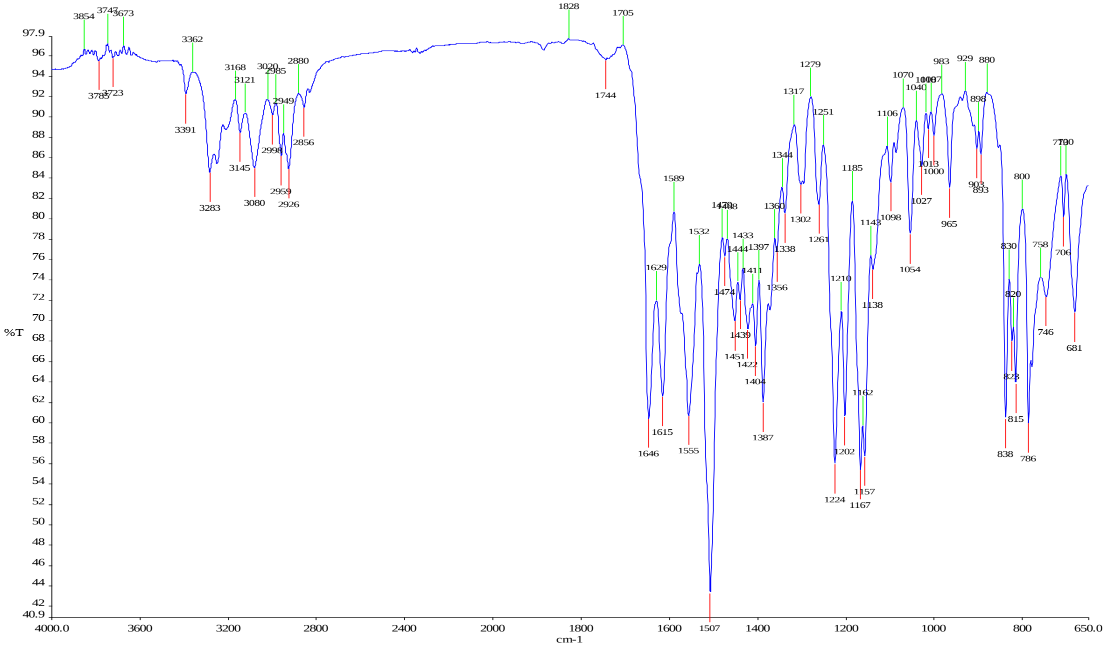
%T
cm-1
4000.0
3600
3200
2800
2400
2000
1800
1600
1400
1200
1000
800
650.0
97.9
40.9
3854
3747
3673
3785
3723
3362
3391
3283
3168
3121
3145
3080
3020
2985
2949
2998
2959
2926
2880
2856
1828
1705
1744
1629
1646
1615
1589
1532
1555
1507
1317
1279
1251
1344
1302
1261
1338
1360
1356
1433
1444
1411
1397
1474
1439
1451
1422
1404
1387
1185
1210
1224
1202
1143
1138
1162
1157
1167
1106
1098
1070
1040
1054
1027
1013
1000
983
965
929
898
880
903
893
800
830
820
758
746
828
815
838
786
706
681

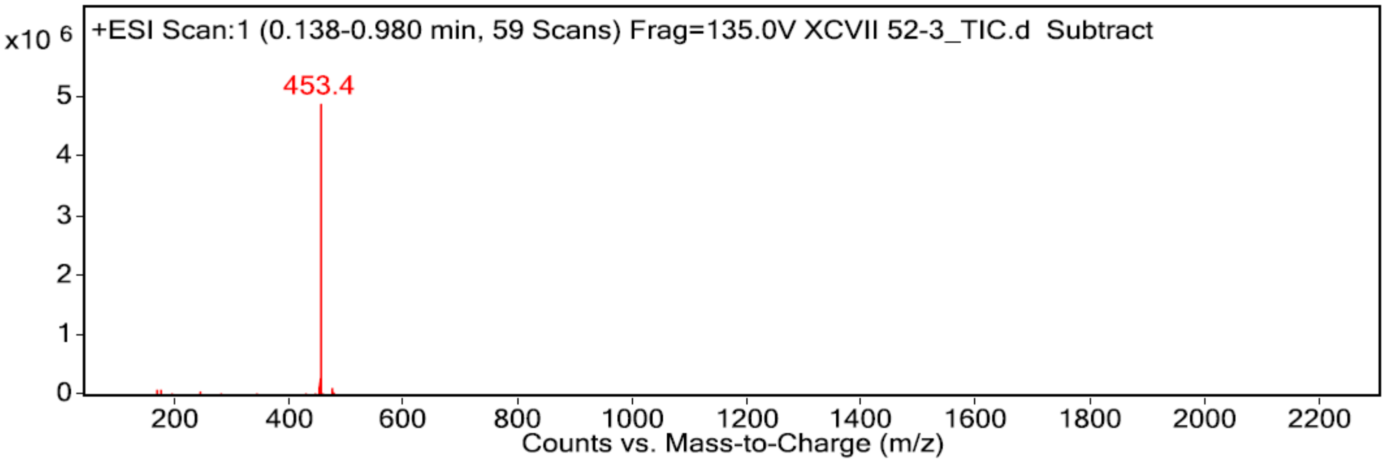
+ESI Scan:1 (0.138-0.980 min, 59 Scans) Frag=135.0V XCVII 52-3_TIC.d Subtract
x10 6
453.4
Counts vs. Mass-to-Charge (m/z)

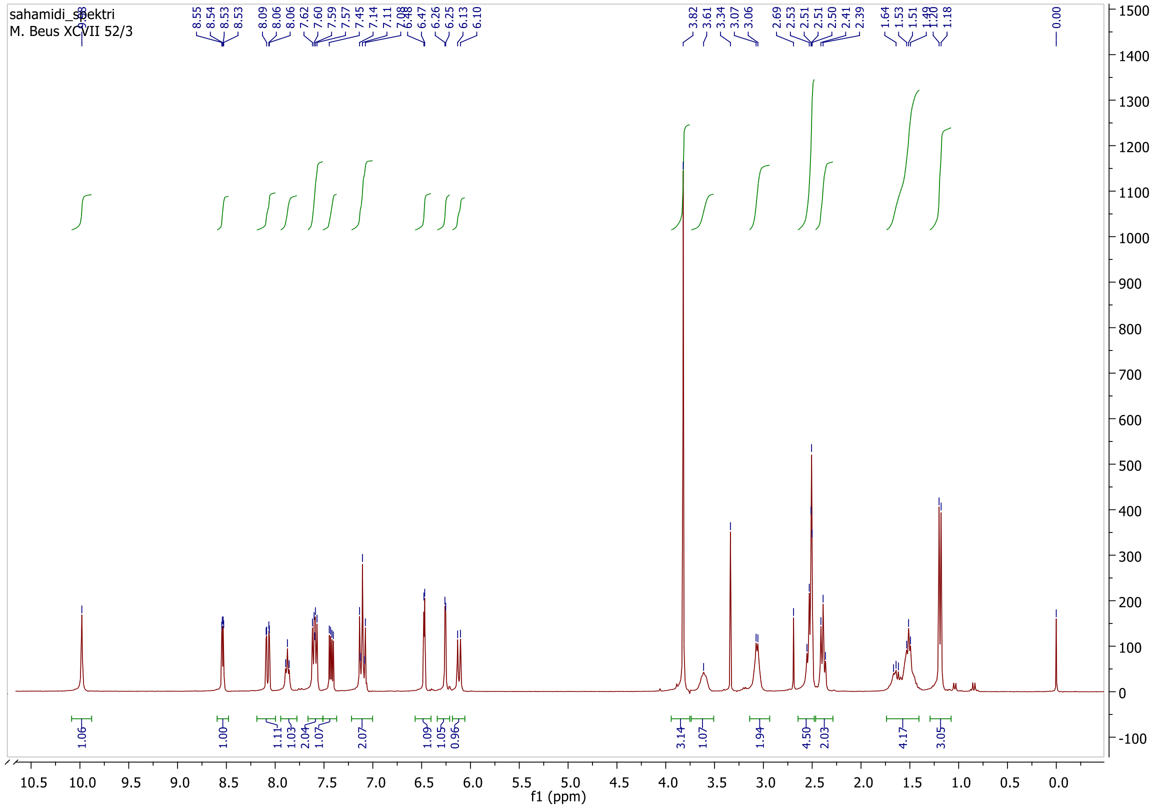
sahamidi_spektri
M. Beus XCVII 52/3
8.55 8.54 8.53 8.53 8.09 8.06 8.06 7.62 7.60 7.59 7.57 7.45 7.14 7.11 7.08 6.48 6.47 6.26 6.25 6.13 6.10
3.82 3.61 3.34 3.07 3.06 2.69 2.53 2.51 2.51 2.50 2.41 2.39 1.64 1.53 1.51 1.49 1.20 1.18 0.00
1.06 1.00 1.11 1.03 2.04 1.07 2.07 1.09 1.05 0.96 3.14 1.07 1.94 4.50 2.03 4.17 3.05
f1 (ppm)

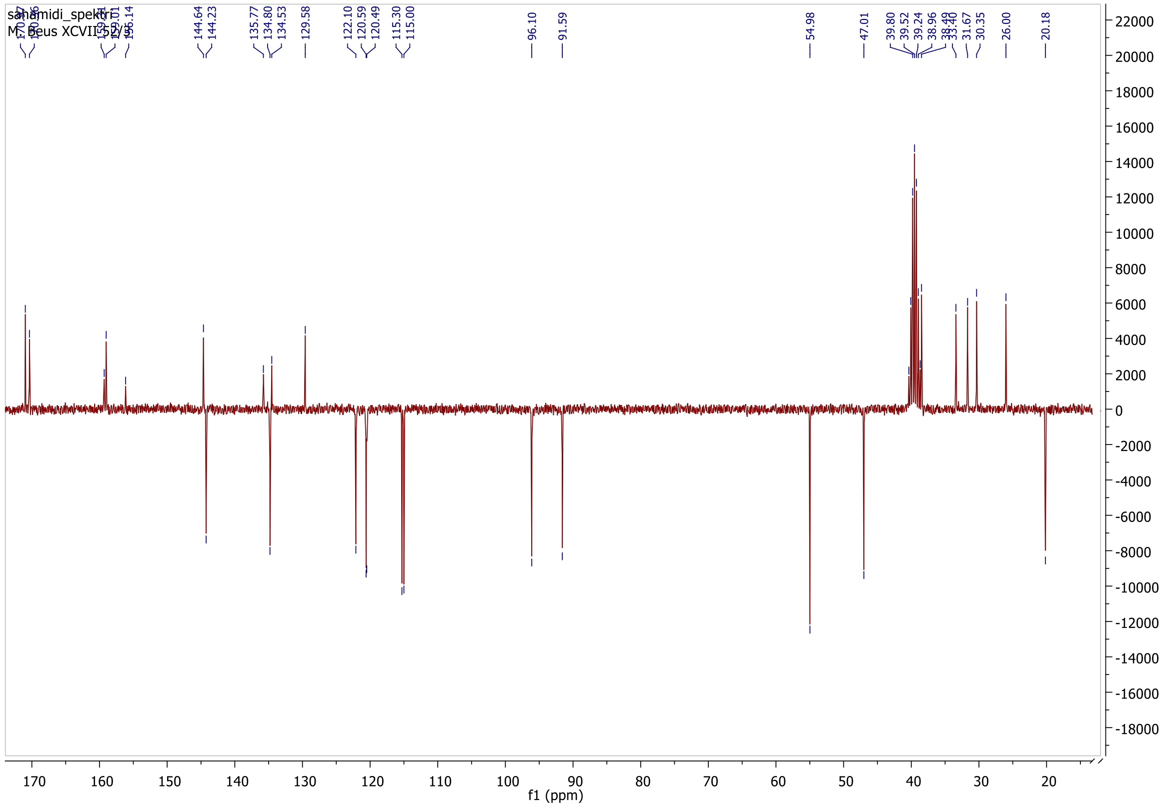

sahamidi_spektri
M. Beus XCVII 52/3
170.97
170.36
159.31
159.01
156.14
144.64
144.23
135.77
134.80
134.53
129.58
122.10
120.59
120.49
115.30
115.00
96.10
91.59
54.98
47.01
39.80
39.52
39.24
38.96
38.49
33.40
31.67
30.35
26.00
20.18
22000
20000
18000
16000
14000
12000
10000
8000
6000
4000
2000
0
-2000
-4000
-6000
-8000
-10000
-12000
-14000
-16000
-18000
170
160
150
140
130
120
110
100
90
80
70
60
50
40
30
20
f1 (ppm)

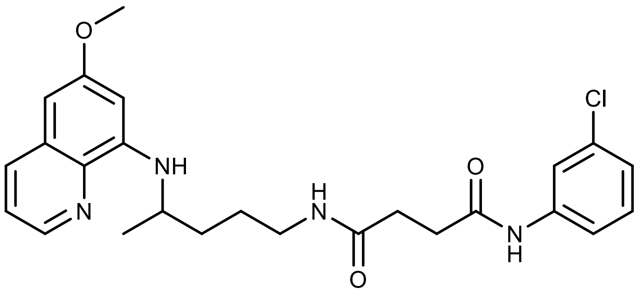
O
NH
N
H
N
O
O
N
H
Cl

**5c**

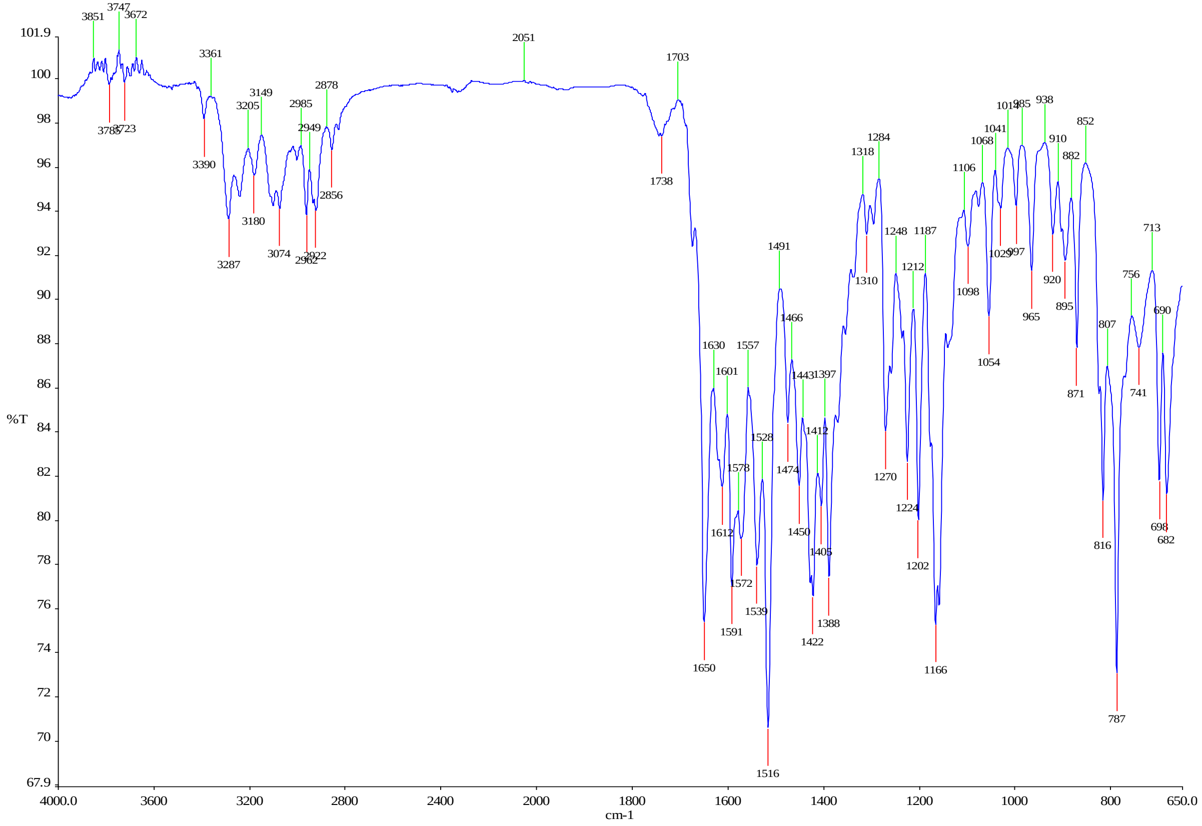
%T
cm-1
101.9
100
98
96
94
92
90
88
86
84
82
80
78
76
74
72
70
67.9
4000.0
3600
3200
2800
2400
2000
1800
1600
1400
1200
1000
800
650.0
3851
3747
3672
3785
3723
3361
3390
3205
3149
3180
3287
3074
2985
2949
2962
2922
2878
2856
2051
1703
1738
1630
1601
1612
1557
1578
1572
1591
1528
1539
1650
1516
1491
1466
1474
1443
1397
1412
1450
1405
1422
1388
1318
1284
1310
1270
1248
1224
1212
1187
1202
1166
1106
1068
1041
1098
1054
997
938
910
920
895
965
882
852
871
807
816
787
756
741
713
690
698
682

| Spectrum Source | Fragmentor Voltage | Collision Energy | Ionization Mode |
|---|---|---|---|
| Peak (1) in "+/- TIC Scan" | 135 | 0 | ESI |

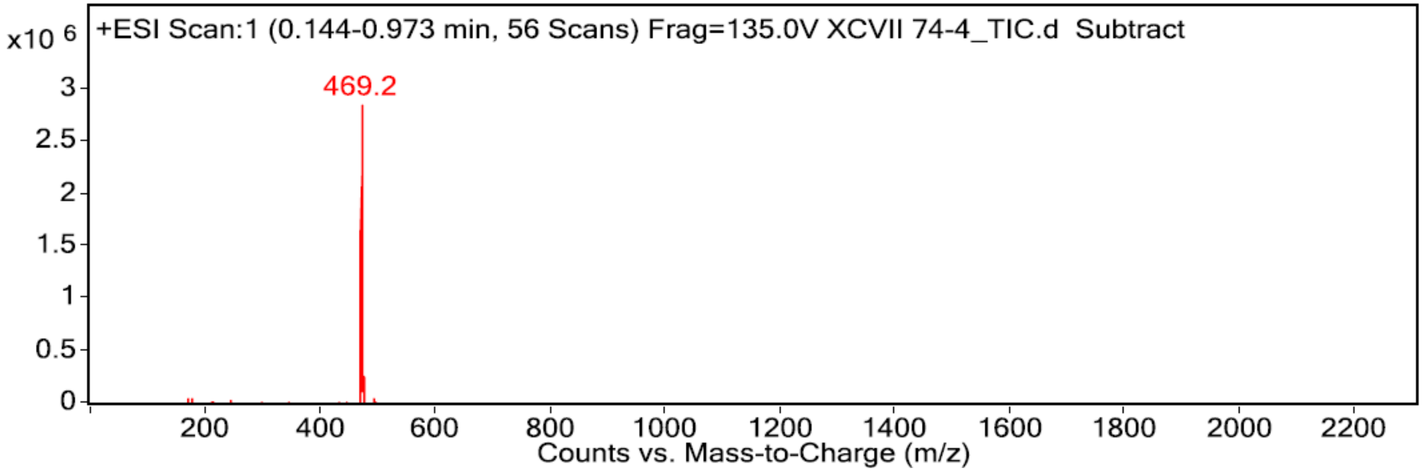


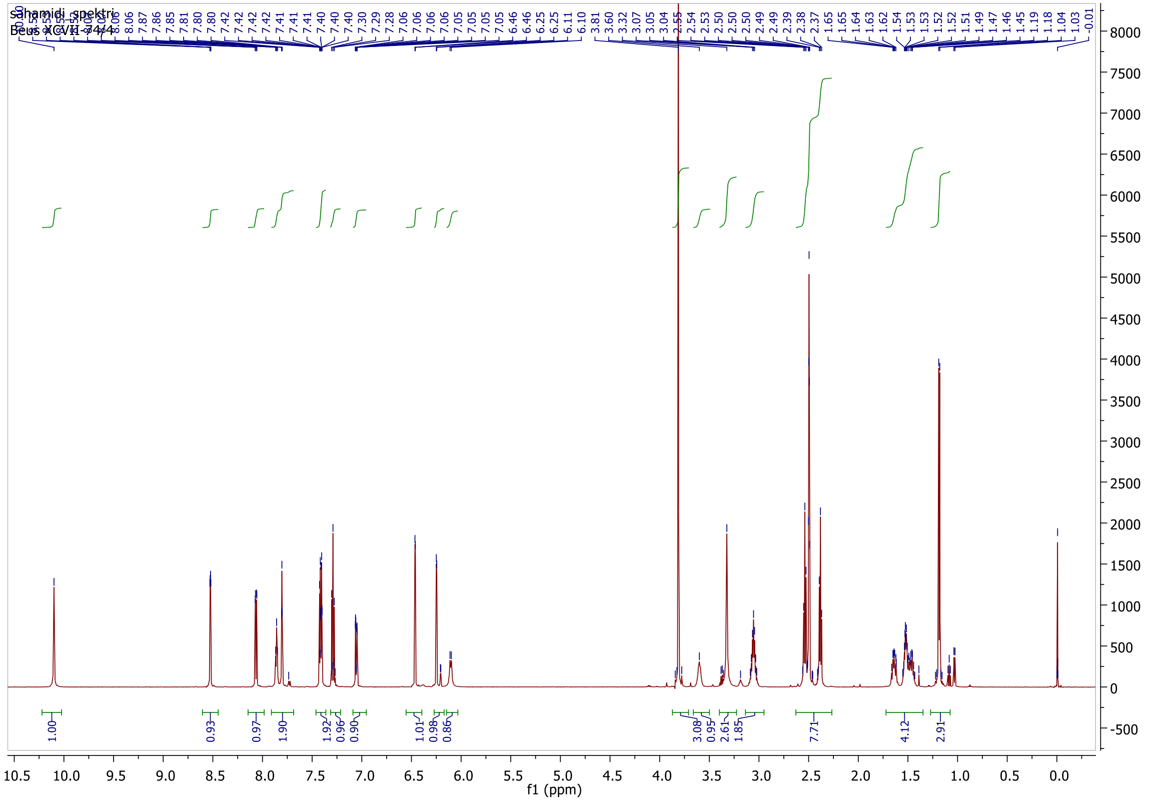

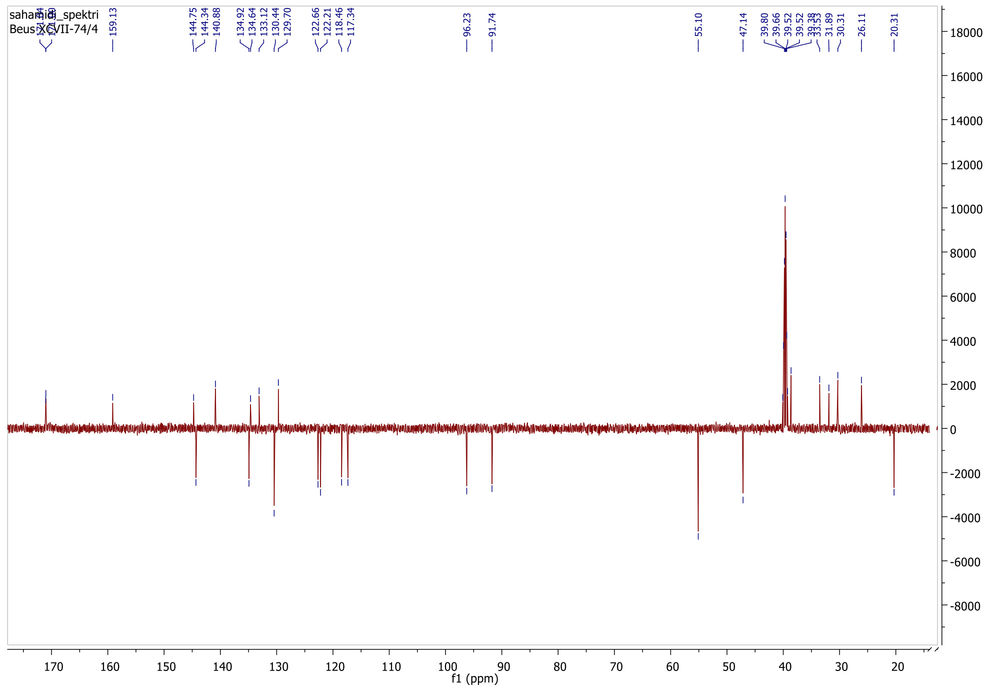

sahamidi_spektri
Beus XCVII-74/4
159.13
144.75
144.34
140.88
134.92
134.64
133.12
130.44
129.70
122.66
122.21
118.46
117.34
96.23
91.74
55.10
47.14
39.80
39.66
39.52
39.52
33.53
31.89
30.31
26.11
20.31
f1 (ppm)

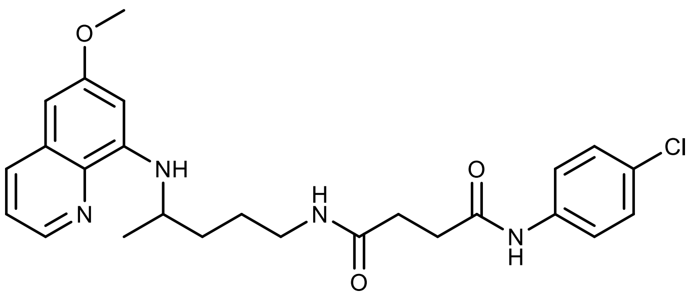

**5d**

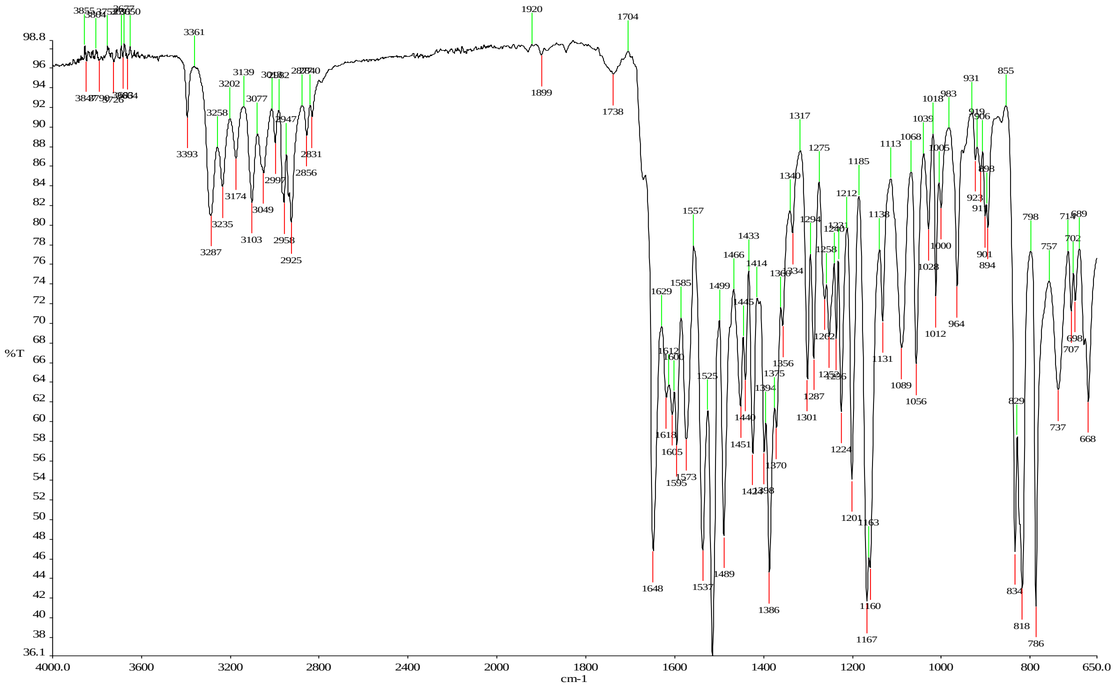

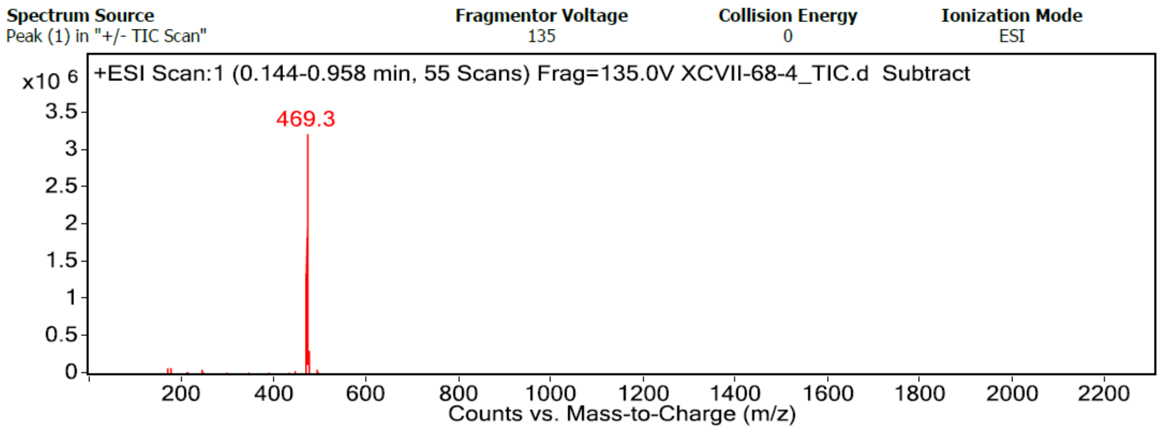

Spectrum Source
Peak (1) in "+/- TIC Scan"
Fragmentor Voltage
135
Collision Energy
0
Ionization Mode
ESI
+ESI Scan:1 (0.144-0.958 min, 55 Scans) Frag=135.0V XCVII-68-4_TIC.d Subtract
x10 6
469.3
Counts vs. Mass-to-Charge (m/z)


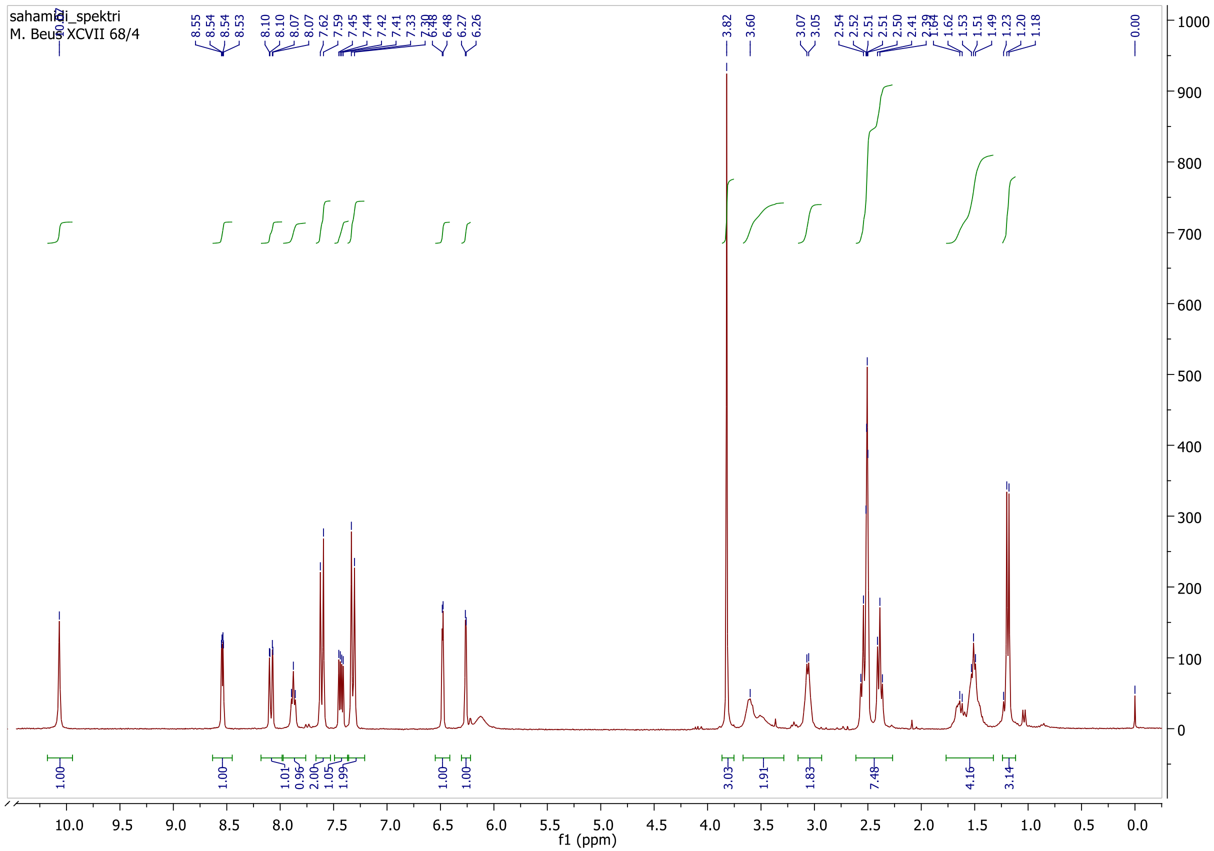

sahamidi_spektri
M. Beus XCVII 68/4
f1 (ppm)

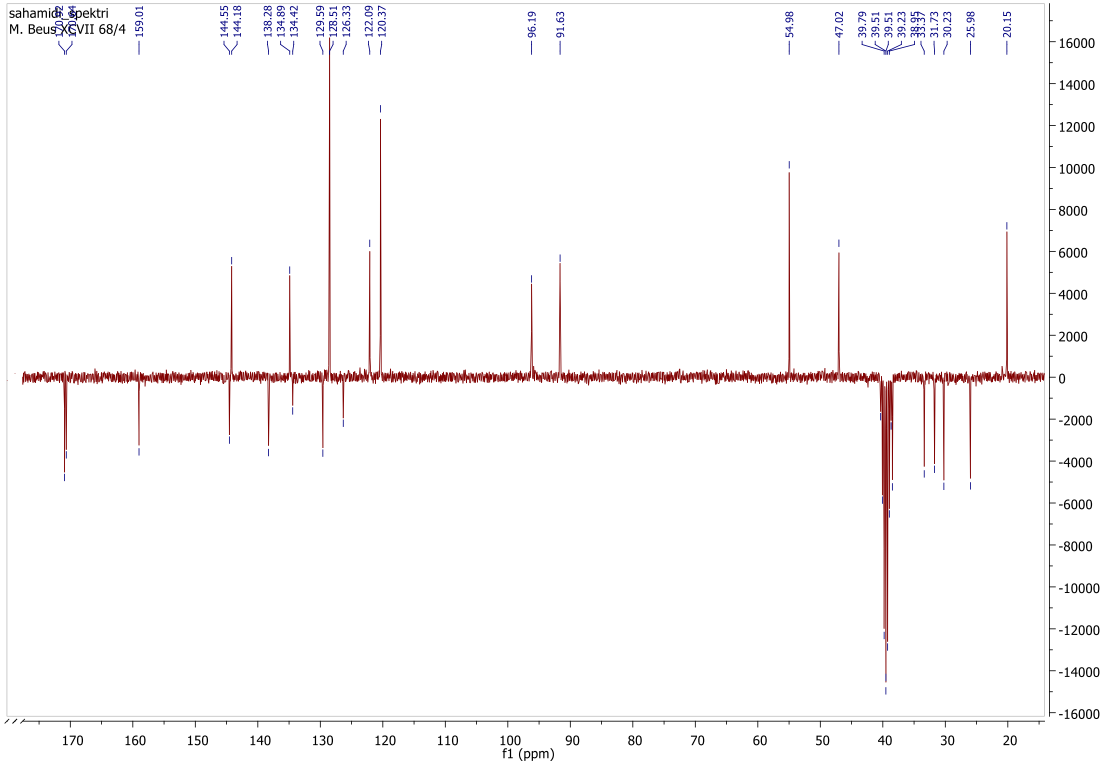
sahamidi_spektri
M. Beus XCVII 68/4
159.01
144.55
144.18
138.28
134.89
134.42
129.59
128.51
126.33
122.09
120.37
96.19
91.63
54.98
47.02
39.79
39.51
39.51
39.23
33.37
31.73
30.23
25.98
20.15
16000
14000
12000
10000
8000
6000
4000
2000
0
-2000
-4000
-6000
-8000
-10000
-12000
-14000
-16000
170
160
150
140
130
120
110
100
90
80
70
60
50
40
30
20
f1 (ppm)

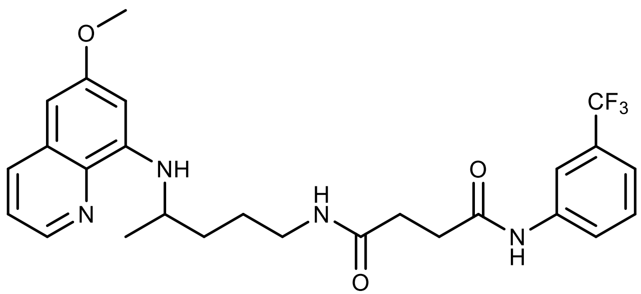
O
NH
N
H
N
O
O
CF3
N
H

**5e**

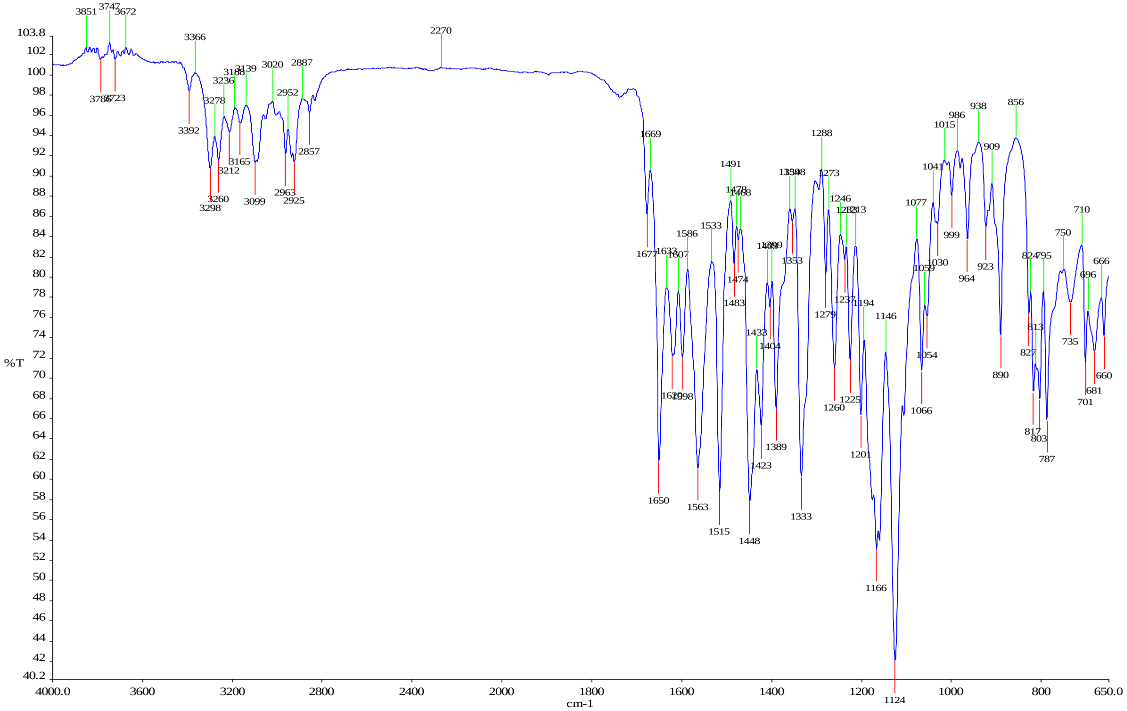
%T
cm-1
103.8
40.2
4000.0
3600
3200
2800
2400
2000
1800
1600
1400
1200
1000
800
650.0
3392
3298
3260
3099
2963
2925
2857
2270
1669
1650
1563
1515
1448
1423
1389
1333
1288
1260
1201
1166
1124
1066
999
964
923
890
856
817
803
787
701
681
660

Peak (1) in "+/- TIC Scan" 135 0 ESI

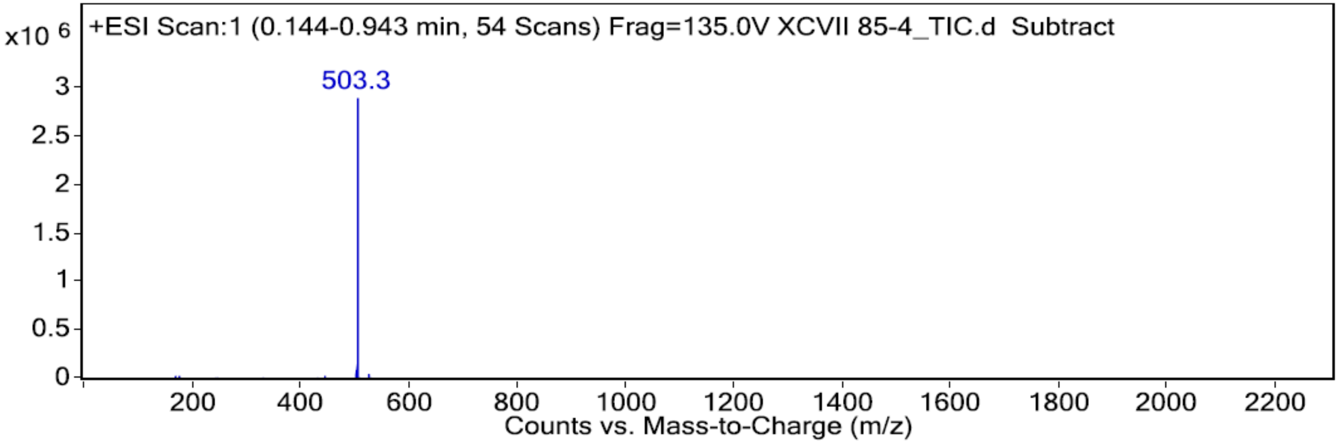


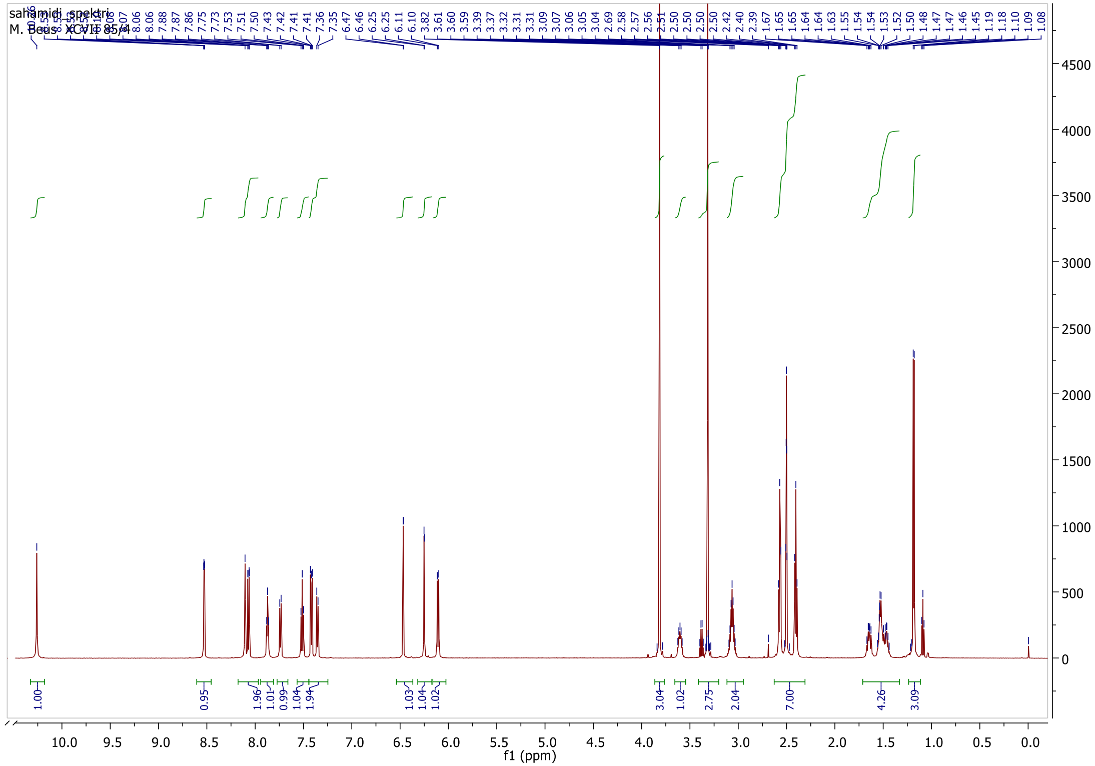

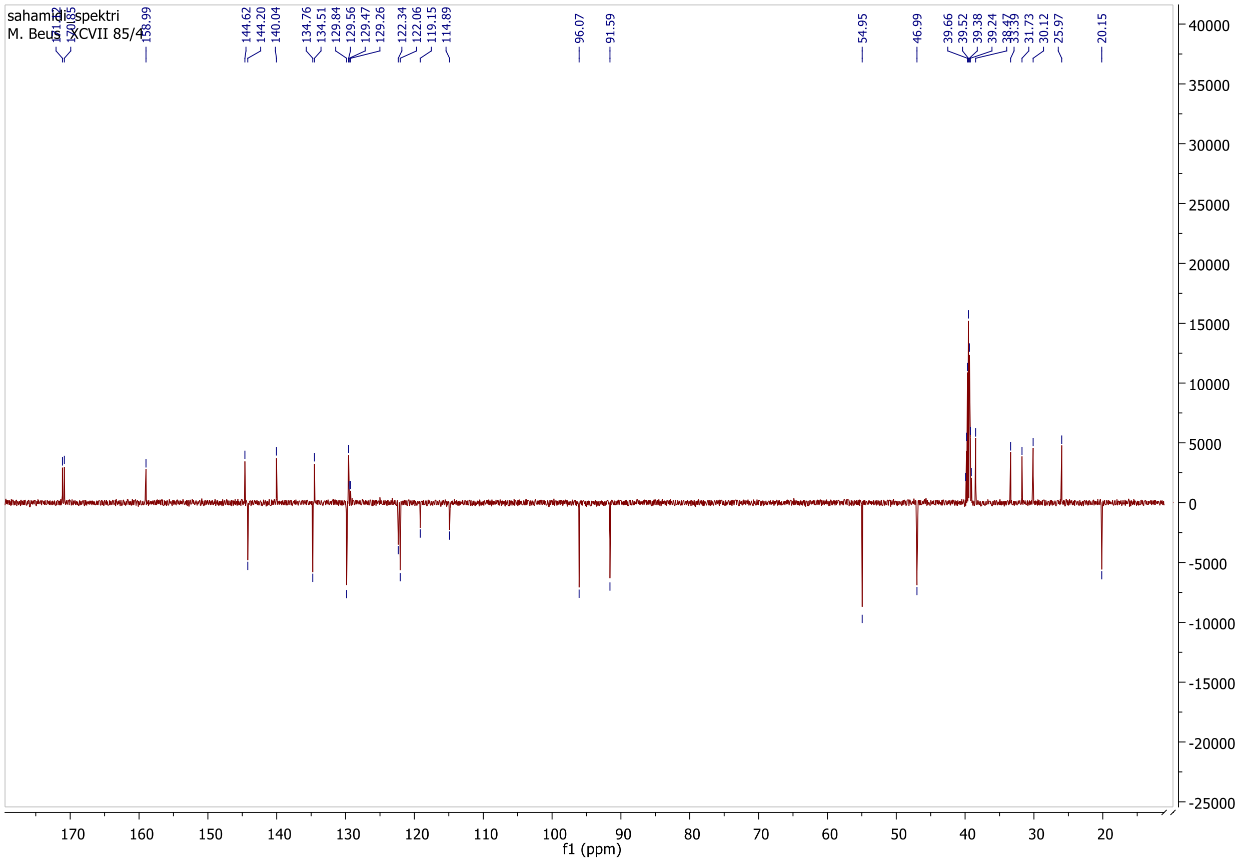
sahamidi spektri
M. Beus XCVII 85/4
158.99
144.62
144.20
140.04
134.76
134.51
129.84
129.56
129.47
129.26
122.34
122.06
119.15
114.89
96.07
91.59
54.95
46.99
39.66
39.52
39.38
39.24
31.73
30.12
25.97
20.15
f1 (ppm)

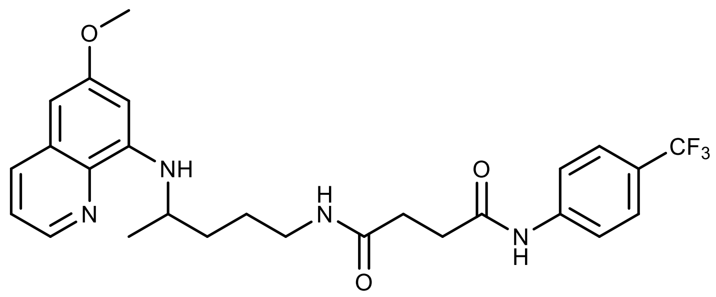

O
NH
N
H
N
O
O
N
H
CF3


**5f**

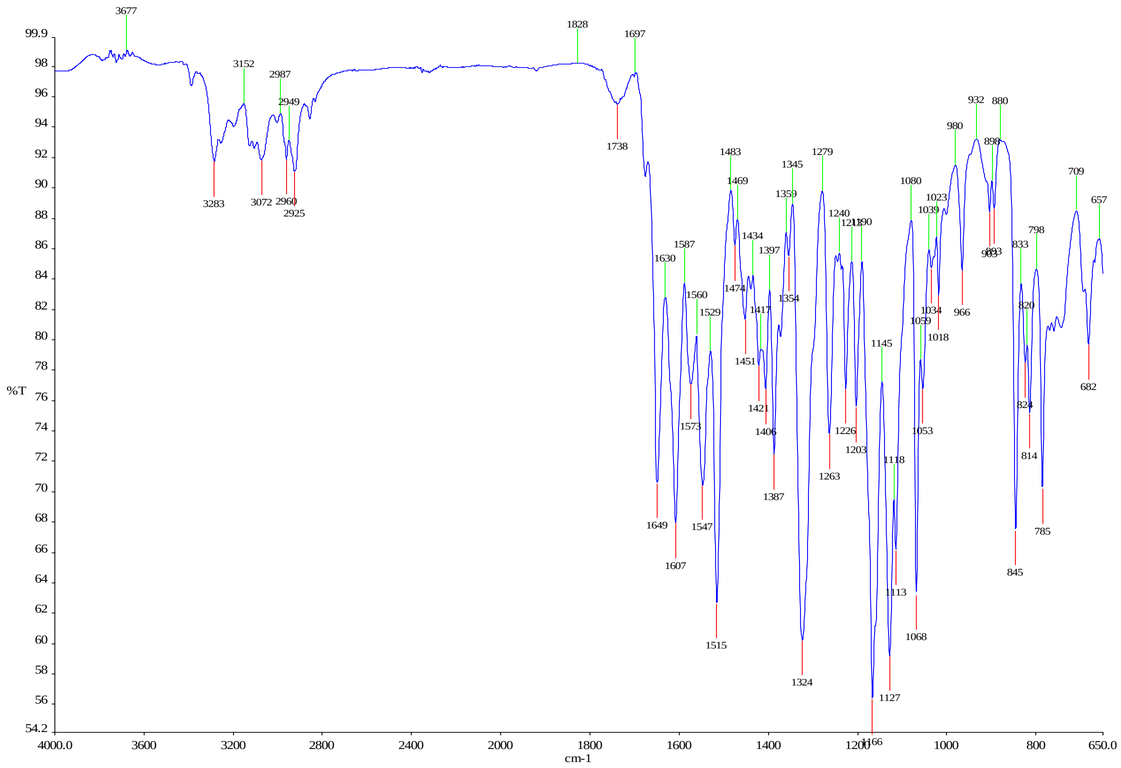

%T
cm-1
99.9
98
96
94
92
90
88
86
84
82
80
78
76
74
72
70
68
66
64
62
60
58
56
54.2
4000.0
3600
3200
2800
2400
2000
1800
1600
1400
1200
1000
800
650.0
3677
3152
2987
2949
3283
3072
2960
2925
1828
1697
1738
1483
1469
1345
1359
1279
1240
121190
1080
1039
1023
980
932
880
898
903
893
966
1630
1587
1560
1529
1434
1397
1417
1474
1354
1451
1421
1406
1573
1387
1263
1226
1203
1649
1607
1547
1515
1324
1145
1118
1113
1127
1166
1034
1059
1018
1053
1068
833
820
824
814
798
845
785
709
657
682

| Spectrum Source | Fragmentor Voltage | Collision Energy | Ionization Mode |
|---|---|---|---|
| Peak (1) in "+/- TIC Scan" | 135 | 0 | ESI |

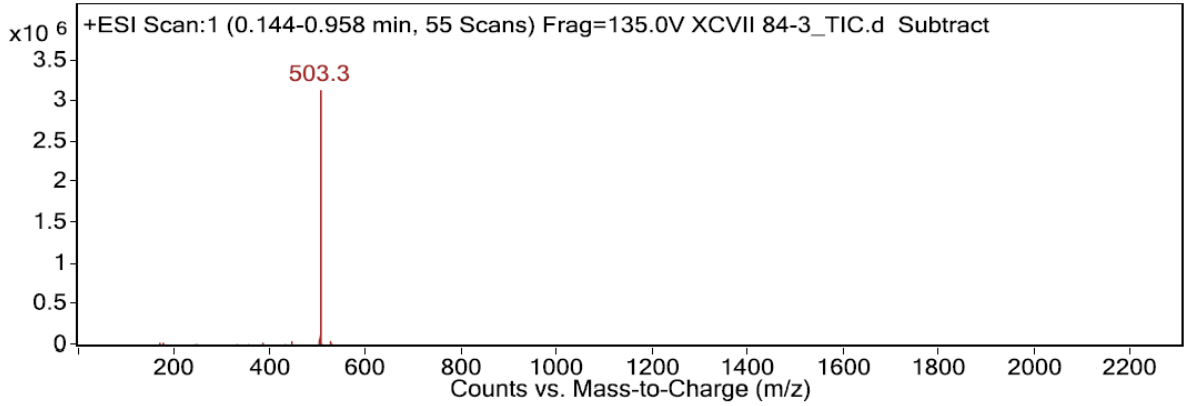


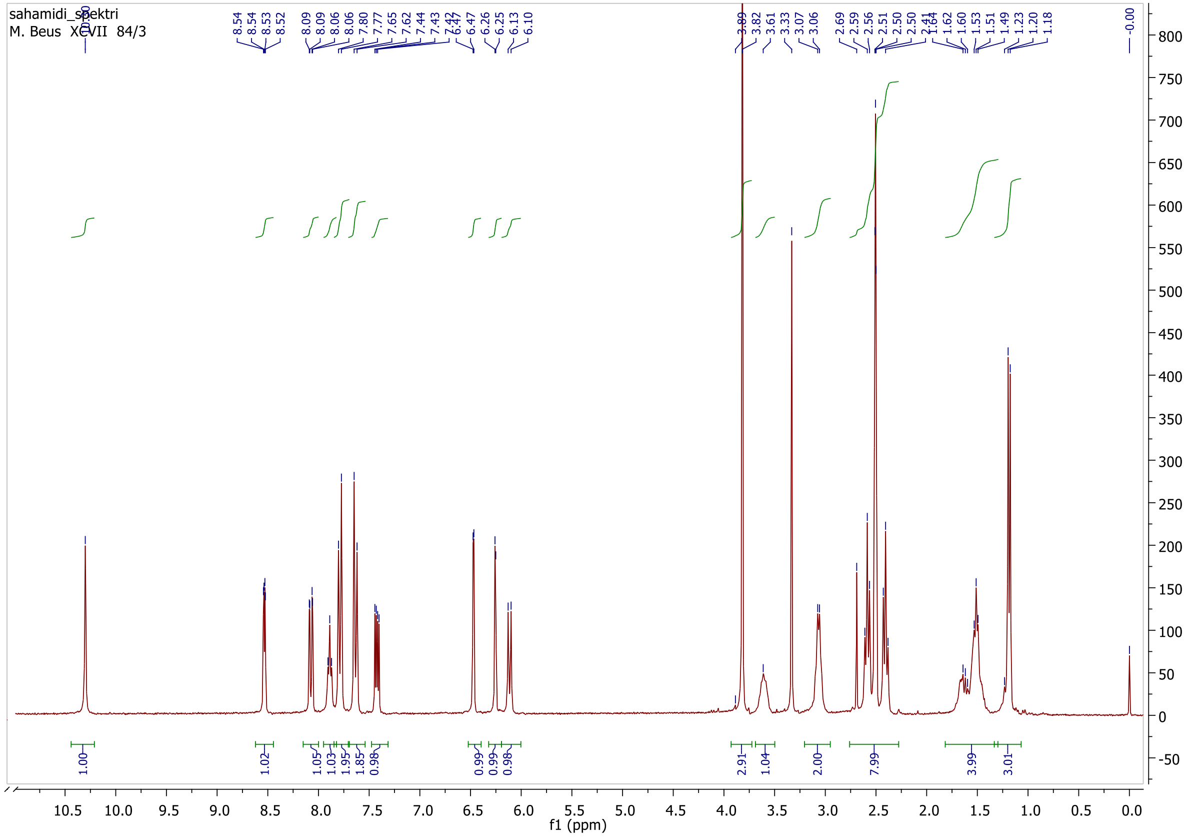

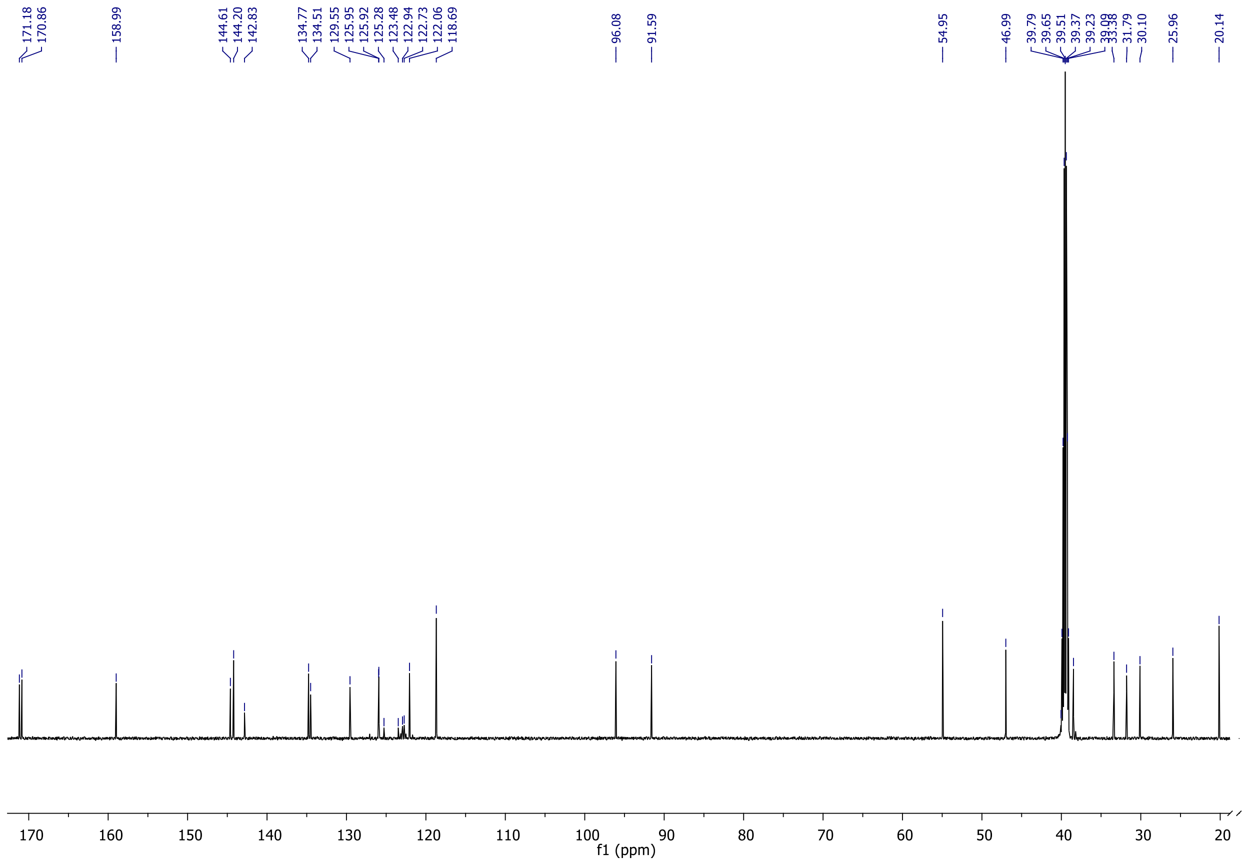
171.18
170.86
158.99
144.61
144.20
142.83
134.77
134.51
129.55
125.95
125.92
125.28
123.48
122.94
122.73
122.06
118.69
96.08
91.59
54.95
46.99
39.79
39.65
39.51
39.37
39.23
31.79
30.10
25.96
20.14
170
160
150
140
130
120
110
100
90
80
70
60
50
40
30
20
f1 (ppm)
